# Inference regarding multiple structural changes in linear models with endogenous regressors^☆^

**DOI:** 10.1016/j.jeconom.2012.05.006

**Published:** 2012-10

**Authors:** Alastair R. Hall, Sanggohn Han, Otilia Boldea

**Affiliations:** a Department of Economics, University of Manchester, UK; b SAS Institute, United States; c Department of Econometrics and Operations Research, Tilburg University, Netherlands

**Keywords:** Structural change, Multiple break points, Instrumental variables estimation

## Abstract

This paper considers the linear model with endogenous regressors and multiple changes in the parameters at unknown times. It is shown that minimization of a Generalized Method of Moments criterion yields inconsistent estimators of the break fractions, but minimization of the Two Stage Least Squares (2SLS) criterion yields consistent estimators of these parameters. We develop a methodology for estimation and inference of the parameters of the model based on 2SLS. The analysis covers the cases where the reduced form is either stable or unstable. The methodology is illustrated via an application to the New Keynesian Phillips Curve for the US.

## 1 Introduction

While it is routine to assume in estimation that the parameters of econometric models are constant over time, there are reasons why this assumption may be questionable. In particular, it can be argued that policy changes and/or exogenous shifts may cause realignments in the relationship between economic variables which are reflected in changes in the parameters. Therefore, it is important to develop methods for detecting parameter instability and also for building models that incorporate this behaviour.

Considerable attention has focused on developing tests for structural instability within the IV or more generally within the Generalized Method of Moments (GMM) framework.^1^ The majority of this literature has focused on the design of tests against the alternative of one structural break. Although these tests are also shown to have non-trivial power against other alternatives, it is clearly desirable to develop procedures that can discriminate between various forms of instability, including multiple unknown breaks. An important step in this direction is taken by Bai and Perron (1998).^2^ Their analysis is in the context of linear regression models estimated via Ordinary Least Squares (OLS). Within their framework, the break points are estimated simultaneously with the regression parameters via minimization of the residual sum of squares. Bai and Perron (1998) establish the consistency and the limiting distribution of the resulting break point fractions. They also propose a sequential procedure for selecting the number of break points in the sample based on various F-statistics for parameter constancy.

While not the only possible form for structural instability, the model with discrete shifts at multiple unknown break points has some appeal in macroeconometric applications because it captures the case where relationships change due to changes in the policy regime or exogenous shifts. However, since Bai and Perron’s (1998) analysis is predicated on the assumption that all explanatory variables are exogenous, their methods cannot be applied to macroeconometric models where the regressors are correlated with the errors.^3^

In this paper, we consider the extension of Bai and Perron’s (1998) framework to linear models with endogenous regressors estimated via IV. There are two common approaches to IV estimation in econometrics: GMM and Two Stage Least Squares (2SLS). We begin by exploring the properties of break points and parameter estimators obtained by minimizing a GMM criterion. In the context of a one break model, we show that the GMM estimator of the break fraction (that indexes the break point) is inconsistent in general and provide a set of conditions under which it has a non-degenerate limiting distribution. Inspection of the proofs indicates that this behaviour stems from construction of the minimand as the square of sums. This structure allows the opportunity for the effects of the misspecification associated with the selection of the wrong break point to offset in the minimand and confound the estimation. In contrast to GMM, the 2SLS minimand is a sum of squares and thus of a more promising construction. This intuition is also implicit in the endogenous regressor model of Caner and Hansen (2004), where the threshold parameter is estimated via 2SLS rather than GMM.

We therefore focus on 2SLS and consider the case in which the break points are estimated simultaneously with the regression parameters via minimization of the residual sum of squares on the second step of the 2SLS estimation. To employ this strategy, it is necessary in the first stage regression to estimate the reduced form for the endogenous regressors in the structural equation of interest and this, of course, requires an assumption about the constancy or lack thereof of these reduced form parameters. In this paper, we consider two scenarios of interest, namely: (i) the parameters in the first stage regression are constant; (ii) the parameters in the first stage regression are subject to discrete shifts within the sample period and the locations of these shifts are estimated *a priori* via a data-based method that satisfies certain conditions. The latter conditions allow the case in which the location of the instability is estimated via an application of Bai and Perron’s (1998) methods to each reduced form equation. Under both scenarios for the reduced form, we establish the consistency of the resulting break fraction estimators and both the consistency and asymptotic normality of the parameter estimators of the equation of interest. However, it turns out that the behaviour of the reduced form impacts on the limiting behaviour of test statistics for parameter change. In the case where the reduced form is stable, we show that the various F-statistics and Wald statistics for testing parameter constancy based on the 2SLS estimator have the same limiting distribution as the analogous statistics for OLS considered by Bai and Perron (1998). However, the corresponding results do not hold if the reduced form is unstable. This failure stems from the limiting behaviour of certain sample moments and is similar to that highlighted by Hansen (2000) in his analysis of the sup-F test of Andrews (1993) when there are changes in the marginal distribution of the regressors. Nevertheless, we are able to propose a simple methodology for estimating the number of breaks in both scenarios described above.

To illustrate our methods, we consider the stability of the New Keynesian Phillips Curve (NKPC) estimated using quarterly data for the US over the period 1968.3–2001.4. The NKPC is of considerable theoretical importance in monetary policy analysis as it is used to identify the forward-looking components of inflation, as well as the trade-off between inflation and unemployment over the cycle. Zhang et al. (2008) observe that empirical studies of the NKPC often reach conflicting conclusions about the importance of key variables in the determination of inflation, and argue this may be due to neglected parameter variation. Zhang et al. (2008) argue that changes in monetary policy regimes may cause changes in the parameters of the NKPC; if true, this would mean that the parameters of the NKPC would exhibit discrete shifts at potentially multiple points in the sample. Zhang et al. (2008) investigate this issue using a methodology based on uncovering break points in the sample via the maximization of Wald statistics for parameter change associated with 2SLS estimation. However, while their methodology has an intuitive appeal, there is no theoretical justification for their methods. In contrast, our methods can be applied to this model under plausible assumptions about the data. Our analysis indicates that there are shifts in the parameters of the appropriate reduced forms and also in the NKPC itself.

In a recent paper, Perron and Yamamoto (2009) have also considered the problem of testing for multiple breaks for linear models with endogenous regressors. Their approach is based on OLS estimation of the structural equation of interest, in essence ignoring the endogeneity of the regressors for the purposes of inference about the breaks. We believe that our 2SLS approach has a number of advantages over an OLS-based approach and highlights just two here for brevity. First, a 2SLS approach naturally involves separate treatment of the structural and reduced form equations and so, using our methodology allows a researcher to determine the breaks in each; whereas an OLS approach, by ignoring the endogeneity, allows breaks in the reduced form potentially to contaminate inferences about breaks in the structural equation. Second, the 2SLS approach yields consistent estimators of the parameters of the structural equation in each regime, whereas the analogous OLS estimates are inconsistent due to the neglected endogeneity.

The outline of the paper is as follows. Section 2 considers estimation based on a GMM minimand. Section 3 lays out the basic structure of the 2SLS estimation of the break point and parameter estimators. Section 4 establishes the properties of the estimators and various tests of parameter change when the reduced form is stable, describes an algorithm for estimation of the number of breaks and also validates our procedures in finite samples via simulations. Section 5 establishes the properties of the estimators when the reduced form is unstable, and proposes a methodology for estimating the number of breaks, partly exploiting the results for the stable reduced form. The finite sample performance of these methods is also evaluated using a small simulation study. Section 6 illustrates our methodology in the context of NKPC estimation for the US. Section 7 concludes. The Mathematical appendix contains sketch proofs of the results in the paper; more detailed proofs are relegated to a supplemental appendix that is available from the authors upon request.

## 2 Inference based on the GMM minimand

Consider the following linear model with one break

(1) $$y_{t}=x_{t}^{\prime }\theta_{0}^{\left(i\right)}+u_{t}\text{,}\;t=1,2,\ldots ,T$$

where $\theta_{0}^{\left(i\right)}=\theta_{0}^{\left(1\right)}$ for $t/T\leq \lambda^{0}$ and $\theta_{0}^{\left(i\right)}=\theta_{0}^{\left(2\right)}$ for $t/T>\lambda^{0},\lambda^{0}\in \left(0,1\right)$ and $\theta_{0}^{\left(1\right)}\neq \theta_{0}^{\left(2\right)}$. Let $x_{t}$ and $\theta_{0}^{\left(i\right)}$ be p×1. We assume that there exists a q×1 vector of variables, $z_{t}$, that are used as instruments for $x_{t}$, where q>p. Define $v_{t}={\left(x_{t}^{\prime },u_{t},z_{t}^{\prime }\right)}^{\prime }$.

For ease of presentation in this section, we assume that $\left\{v_{t}\right\}$ is an independent sequence but in line with the model in (1), we allow the data generation process for $v_{t}$ to change (potentially) at $\left[T\lambda^{0}\right]$. These restrictions are embodied in the following assumption.

Assumption 1 (i) $v_{t}$ is independently distributed; (ii) $E\left[z_{t}x_{t}^{\prime }\right]=M_{1},\;t/T\leq \lambda^{0},E\left[z_{t}x_{t}^{\prime }\right]=M_{2},\;t/T>\lambda^{0}$, $\mathrm{rank}\;M_{i}=p,\;i=1,2$ (iii) $E\left[z_{t}u_{t}\right]=0$, (iv) $\sup_{t}E{\left\|v_{t}\right\|}^{4}<\infty$.

For convenience of notation, we define the matrices:

$$N_{1}\left(\lambda \right)=\min\left(\lambda ,\;\lambda_{0}\right)M_{1}+\max\left(\lambda -\lambda_{0},\;0\right)M_{2}$$

$$N_{2}\left(\lambda \right)=\max\left(\lambda_{0}-\lambda ,\;0\right)M_{1}+\min\left(1-\lambda ,\;1-\lambda_{0}\right)M_{2}\text{.}$$

If the researcher knows there is a break but is unaware of its location, a natural approach is to estimate the location by minimizing the GMM criterion over all candidate partitions. Following Andrews (1993), GMM estimation of θ(λ) for each candidate break fraction, λ, is based on $E\left[f\left(v_{t},\theta \left(\lambda \right);\lambda \right)\right]=0$ where

(2) $$f\left(v_{t},\theta \left(\lambda \right);\lambda \right)=\left[\;\begin{array}{c} z_{t}\left\{y_{t}-x_{t}^{\prime }\theta_{1}\left(\lambda \right)\right\}I_{t,T}\left(\lambda \right)\\ z_{t}\left\{y_{t}-x_{t}^{\prime }\theta_{2}\left(\lambda \right)\right\}\left\{1-I_{t,T}\left(\lambda \right)\right\} \end{array}\;\right]$$

where $\theta \left(\lambda \right)={\left(\theta_{1}{\left(\lambda \right)}^{\prime },\theta_{2}{\left(\lambda \right)}^{\prime }\right)}^{\prime },\theta_{i}\left(\lambda \right)\in \Theta \subset {ℜ}^{p}$ and $I_{t,T}\left(\lambda \right)$ is an indicator variable that takes the value one if t/T≤λ and the value zero otherwise. The partial-sum GMM estimators of ${\left[\theta_{1}{\left(\lambda \right)}^{\prime },\theta_{2}{\left(\lambda \right)}^{\prime }\right]}^{\prime }$ are defined as follows:

(3) $${\overset{ˆ}{\theta }}_{T}\left(\lambda \right)=\underset{\theta \left(\lambda \right)\in \Theta \times \Theta }{\mathrm{argmin}}Q_{T}\left(\theta \left(\lambda \right);\lambda \right)$$

where ${\overset{ˆ}{\theta }}_{T}\left(\lambda \right)=\mathrm{vec}\left[{\overset{ˆ}{\theta }}_{1,T}\left(\lambda \right),{\overset{ˆ}{\theta }}_{2,T}\left(\lambda \right)\right],Q_{T}\left(\theta \left(\lambda \right);\lambda \right)=g_{T}{\left(\theta \left(\lambda \right);\lambda \right)}^{\prime }W_{T}\left(\lambda \right)g_{T}\left(\theta \left(\lambda \right);\lambda \right),g_{T}\left(\theta \left(\lambda \right);\lambda \right)=T^{-1}\sum_{t=1}^{T}f\left(v_{t},\theta \left(\lambda \right);\lambda \right),W_{T}\left(\lambda \right)=\mathrm{diag}\left\{W_{1,T}\left(\lambda \right),W_{2,T}\left(\lambda \right)\right\}$ and $W_{i,T}\left(\lambda \right)$ is a q×q deterministic matrix. We assume $W_{i,T}\left(\lambda \right)$ does not depend on θ(λ) but may depend on T. Thus we are considering a “first-step” GMM estimation in which the weighting matrix is a matrix of constants. The advantage of this restriction is that it considerably simplifies the analysis.^4^

Given a set of GMM estimations over λ∈Λ⊂(0,1), the break point estimator is

(4) $${\overset{ˆ}{\lambda }}_{T}=\underset{\lambda \in \Lambda }{\mathrm{argmin}}\underset{\theta \left(\lambda \right)\in \Theta \times \Theta }{\mathrm{argmin}}Q_{T}\left(\theta \left(\lambda \right);\lambda \right)\text{.}$$

This section shows that ${\overset{ˆ}{\lambda }}_{T}$ is not consistent for $\lambda_{0}$ under reasonable conditions. To establish this result, we introduce the following assumptions.

Assumption 2 $E\left[f\left(v_{t},\theta \left(\lambda^{0}\right);\lambda^{0}\right)\right]=0$ for $\theta_{0}\left(\lambda^{0}\right)={\left({\theta_{0}^{\left(1\right)}}^{\prime },{\theta_{0}^{\left(2\right)}}^{\prime }\right)}^{\prime }$.

Assumption 3 Set $b_{t}=\mathrm{vec}\left[z_{t}u_{t},\mathrm{vec}\left\{z_{t}x_{t}^{\prime }-\overset{¯}{M}\left(\lambda \right)\right\}\right]$ for $\overset{¯}{M}\left(\lambda \right)=I_{t,T}\left(\lambda \right)M_{1}+\left(1-I_{t,T}\left(\lambda \right)\right)M_{2}$. Define $T^{-1/2}\sum_{t=1}^{\left[Tr\right]}b_{t}\Rightarrow \Omega^{1/2}B_{m}\left(r\right)$ where $B_{m}\left(r\right)$ is an m×1 vector of standard Brownian motions, m=(p+1)q and $\Omega =\Omega^{1/2}{\Omega^{1/2}}^{\prime }$ is a positive definite (pd) finite matrix.

Assumption 4 The minimum eigenvalues of $N_{i}{\left(\lambda \right)}^{\prime }N_{i}\left(\lambda \right),\;i=1,2$, are bounded away from zero uniformly in λ∈Λ.

Assumption 5 $W_{i,T}\left(\lambda \right)$ is a deterministic, positive semi-definite matrix that converges to $W_{i}\left(\lambda \right)$, a pd matrix, for all λ and i=1,2.

Assumption 2 states that the population moment condition is valid at the true parameter values and at the true break. Assumption 3 states the convergence results needed to underpin the analysis. Assumption 4 ensures that the partial sum GMM estimators defined below are identified; notice that it implies $M_{1}$ and $M_{2}$ are full rank.

Our first result involves the population analog to the GMM minimand. Define ${\overset{˜}{Q}}_{T}\left(\theta \left(\lambda \right);\lambda \right)=E\left[Q_{T}\left(\theta \left(\lambda \right);\lambda \right)\right]$ and its limit as $\lim_{T\to \infty }{\overset{˜}{Q}}_{T}\left(\theta \left(\lambda \right);\lambda \right)=\overset{˜}{Q}\left(\theta \left(\lambda \right);\lambda \right)$.

Proposition 1 *If Eq.* (1) *and* Assumptions 1, 2, 4 and 5 *hold then:* $\overset{˜}{Q}\left(\theta_{*}\left(\lambda \right);\lambda \right)=0$ *in the following cases*

(i) $\lambda =\lambda^{0}:\theta_{*}\left(\lambda \right)=\theta \left(\lambda^{0}\right)={\left({\theta_{0}^{\left(1\right)}}^{\prime },{\theta_{0}^{\left(2\right)}}^{\prime }\right)}^{\prime }$ *;*
(ii) $\lambda <\lambda^{0}:\theta_{0}^{\left(1\right)}-\theta_{0}^{\left(2\right)}\in N\left(M_{1}-M_{2}\right),\theta_{*}\left(\lambda \right)={\left[{\theta_{0}^{\left(1\right)}}^{\prime },{\theta_{*}^{\left(2\right)}\left(\lambda \right)}^{\prime }\right]}^{\prime }$ *where*
  $$\theta_{*}^{\left(2\right)}\left(\lambda \right)=\frac{\left(\lambda^{0}-\lambda \right)\theta_{0}^{\left(1\right)}+\left(1-\lambda^{0}\right)\theta_{0}^{\left(2\right)}}{1-\lambda }$$
  *and* N(A) *denotes the nullspace of a matrix* A *;*
(iii) $\lambda >\lambda^{0}:\theta_{0}^{\left(1\right)}-\theta_{0}^{\left(2\right)}\in N\left(M_{1}-M_{2}\right),\theta_{*}\left(\lambda \right)={\left[{\theta_{*}^{\left(1\right)}\left(\lambda \right)}^{\prime },{\theta_{0}^{\left(2\right)}}^{\prime }\right]}^{\prime }$ *where*
  $$\theta_{*}^{\left(1\right)}\left(\lambda \right)=\frac{\lambda^{0}\theta_{0}^{\left(1\right)}+\left(\lambda -\lambda^{0}\right)\theta_{0}^{\left(2\right)}}{\lambda }\text{.}$$

Remark 1 Proposition 1 indicates that under the condition $\theta_{0}^{\left(1\right)}-\theta_{0}^{\left(2\right)}\in N\left(M_{1}-M_{2}\right)$ there is a value of the parameters that sets the population analog to the GMM minimand equal to zero for every choice of λ. Notice that this value of θ depends on λ. Thus, the population analog of the GMM minimand does not have a unique minimum in θ(λ) for λ∈(0,1).

Remark 2 One case in which the condition $\theta_{0}^{\left(1\right)}-\theta_{0}^{\left(2\right)}\in N\left(M_{1}-M_{2}\right)$ is trivially satisfied is where $M_{1}=M_{2}$, and thus $E\left[x_{t}z_{t}^{\prime }\right]$ remains constant throughout the sample. Notice however, that this moment constancy is sufficient but not necessary for the condition to hold.

Given Proposition 1, we have the following result.

Proposition 2 *If* Assumptions 1–5 *hold and* $\theta_{0}^{\left(1\right)}-\theta_{0}^{\left(2\right)}\in N\left(M_{1}-M_{2}\right)$*then* ${\overset{ˆ}{\theta }}_{T}\left(\lambda \right)\overset{p}{\to }\theta_{*}\left(\lambda \right)$ *uniformly in* λ *where* $\theta_{*}\left(\lambda \right)$ *is defined in* Proposition *.*

The next proposition presents the limiting properties of the break fraction estimator under the conditions on the true parameters in Proposition 2.

Proposition 3 *If* Assumptions 1–5 *hold and* $\theta_{0}^{\left(1\right)}-\theta_{0}^{\left(2\right)}\in N\left(M_{1}-M_{2}\right)$

$${\overset{ˆ}{\lambda }}_{T}\Rightarrow \underset{\lambda \in \Lambda }{\mathrm{argmin}}\left\{Q_{1}\left(\lambda ;\;\lambda_{0}\right)+Q_{2}\left(\lambda ;\;\lambda_{0}\right)\right\}$$

*where* $Q_{i}\left(\lambda ,\lambda^{0}\right)=\xi_{i}{\left(\lambda \right)}^{\prime }\Xi_{i}\left(\lambda \right)\xi_{i}\left(\lambda \right),\Xi_{i}\left(\lambda \right)={\left[I_{q}-N_{i}\left(\lambda \right)H_{i}\left(\lambda \right)\right]}^{\prime }W_{i}\left(\lambda \right)\left[I_{q}-N_{i}\left(\lambda \right)H_{i}\left(\lambda \right)\right],H_{i}\left(\lambda \right)={\left[N_{i}{\left(\lambda \right)}^{\prime }W_{i}\left(\lambda \right)N_{i}\left(\lambda \right)\right]}^{-1}N_{i}{\left(\lambda \right)}^{\prime }W_{i}\left(\lambda \right)$*,*

$$\xi_{1}\left(\lambda \right)=V_{zu}\left(\lambda \right)+\left\{1-I_{\lambda }\left(\lambda^{0}\right)\right\}\left\{\left[{\left(\theta_{0}^{\left(1\right)}-\theta_{0}^{\left(2\right)}\right)}^{\prime }⊗I_{q}\right]\left[\frac{\left(\lambda -\lambda^{0}\right)}{\lambda }V_{\mu }\left(\lambda^{0}\right)-\frac{\lambda^{0}}{\lambda }\left[V_{\mu }\left(\lambda \right)-V_{\mu }\left(\lambda^{0}\right)\right]\;\right]\right\}\text{,}$$

$$\xi_{2}\left(\lambda \right)=V_{zu}\left(1\right)-V_{zu}\left(\lambda \right)+\left\{I_{\lambda }\left(\lambda^{0}\right)\right\}\left\{\left[{\left(\theta_{0}^{\left(1\right)}-\theta_{0}^{\left(2\right)}\right)}^{\prime }⊗I_{q}\right]\left[\frac{\left(1-\lambda^{0}\right)}{\left(1-\lambda \right)}\left[V_{\mu }\left(\lambda^{0}\right)-V_{\mu }\left(\lambda \right)\right]-\frac{\left(\lambda^{0}-\lambda \right)}{\left(1-\lambda \right)}\left[V_{\mu }\left(1\right)-V_{\mu }\left(\lambda^{0}\right)\right]\;\right]\right\}\text{,}$$

$I_{\lambda }\left(\lambda^{0}\right)$ *is an indicator variable that takes the value one if* $\lambda \leq \lambda^{0}$ *and zero otherwise, and* ${\left[V_{zu}{\left(\lambda \right)}^{\prime },V_{\mu }{\left(\lambda \right)}^{\prime }\right]}^{\prime }=\Omega^{1/2}B_{m}\left(\lambda \right)$ *with* $V_{zu}\left(\lambda \right)$ *of dimension* q×1*.*

Remark 3 Proposition 3 indicates that ${\overset{ˆ}{\lambda }}_{T}$ converges to a non-degenerate random variable and is thus not consistent for $\lambda^{0}$ under the conditions of the proposition.

Remark 4 While we focus on the one break model, the inconsistency result generalizes to the multiple break model under certain conditions. For example, if two adjacent regimes satisfy the conditions of our one break model.

To illustrate the nature of the limiting distribution in Proposition 3, we simulate the behaviour of ${\overset{ˆ}{\lambda }}_{T}$ in the following model.

*One break model*: The data generating process for the structural equation is

(5) $$y_{t}={\left[1,\;x_{t}\right]}^{\prime }\beta_{1}^{0}+u_{t}\text{,}\;\text{for }t=1,\ldots ,\left[T/2\right]={\left[1,\;x_{t}\right]}^{\prime }\beta_{2}^{0}+u_{t}\text{,}\;\text{for }t=\left[T/2\right]+1,\ldots ,T\text{.}$$

The reduced form equation for the scalar variable $x_{t}$ is

(6) $$x_{t}=\left[1,z_{t}^{\prime }\right]\delta +v_{t}\text{,}\;\text{for }t=1,\ldots ,T$$

where δ is the (q+1)×1 vector. The errors are generated as follows: ${\left(u_{t},v_{t}\right)}^{\prime }\;∼IN\left(0_{2\times 1},\Omega \right)$ where the diagonal elements of Ω are equal to one and the off-diagonal elements are equal to 0.5. The instrumental variables, $z_{t}$, are generated via $z_{t}∼\mathrm{i.i.d}\;N\left(0_{q\times 1},I_{q}\right)$. The specific parameter values are as follows: (i) T=480; (ii) $\left(\beta_{1}^{0},\beta_{2}^{0}\right)=\left({\left[1,0.1\right]}^{\prime },{\left[-1,-0.1\right]}^{\prime }\;\right)$; (iii) q=4; (iv) $\delta ={\left[1,d^{\prime }\right]}^{\prime }$ where the elements of d are identical and chosen to yield the population $R^{2}=0.5$ for the regression in (6).^5^ 1000 simulations are performed.

**Fig. 1:**
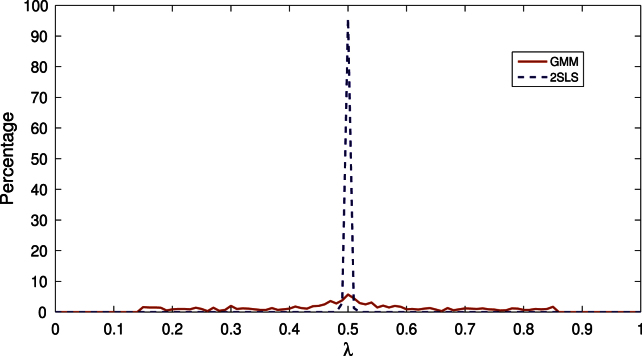
Distribution of estimated break fractions in the one break model.

Fig. 1 contains a plot of the empirical distribution of ${\overset{ˆ}{\lambda }}_{T}$ when Λ=[0.15,0.85]. The distribution has mode around the true break fraction, $\lambda_{0}=0.5$, but is also relatively diffuse over Λ.^6^

**Fig. 2:**
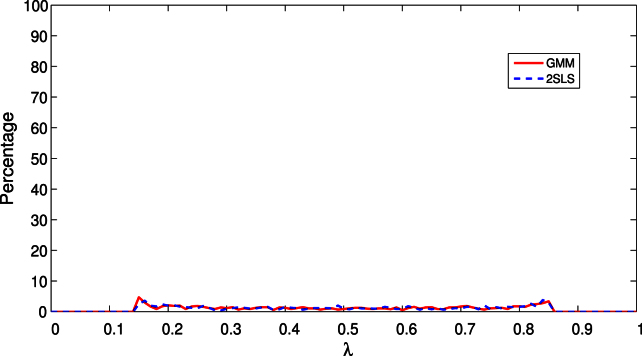
Distribution of estimated break fractions in the no break model.

For purposes of comparison, we also simulated the behaviour of ${\overset{ˆ}{\lambda }}_{T}$ in a model with no breaks and $\left(\beta_{1}^{0},\beta_{2}^{0}\right)={\left[1,0.1\right]}^{\prime }$, that is when it is assumed that there is one break but in fact there are none; all other aspects of the design are the same as the one-break model above. As can be seen from Fig. 2, the peak at λ=0.5 is absent but the distribution of the break fraction estimators is similarly diffuse in the no-break and one-break models.

Propositions 1–3 indicate that a break-point estimation strategy based on the GMM minimand, while intuitively appealing at first sight, is flawed. This leaves us searching for an alternative approach for making valid inference in the multiple-break linear model with endogenous regressors. A way forward is suggested by inspection of the proof of Proposition 1. The source of the inconsistency lies in the structure of the minimand in (4). The minimand is a quadratic form in the sample moments, that is the square of sums. This structure affords the opportunity for the effects of misspecification to offset within the minimand. Such an opportunity is not afforded if the minimand is a sum of squares. Estimation based on a 2SLS minimand has exactly this structure, and in the remainder of this paper we demonstrate that this approach is simple to implement, yields consistent estimators of both the break-fractions and structural parameters and is also a convenient framework for inference within the multiple-break linear model with endogenous regressors.

## 3 Estimation based on 2SLS

Consider the case in which the equation of interest is a linear regression model with m breaks, that is

(7) $$y_{t}=x_{t}^{\prime }\beta_{x,i}^{0}+z_{1,t}^{\prime }\beta_{z_{1},i}^{0}+u_{t}\text{,}\;i=1,\ldots ,m+1,\;t=T_{i-1}^{0}+1,\ldots ,T_{i}^{0}$$

where $T_{0}^{0}=0$ and $T_{m+1}^{0}=T$. In this model, $y_{t}$ is the dependent variable, $x_{t}$ is a $p_{1}\times 1$ vector of explanatory variables, $z_{1,t}$ is a $p_{2}\times 1$ vector of exogenous variables including the intercept, and $u_{t}$ is a mean zero error. We define $p=p_{1}+p_{2}$. Given that some regressors are endogenous, it is plausible that (7) belongs to a system of structural equations and thus, for simplicity, we refer to (7) as the “structural equation”.

As usual in the literature, we require the break points to be asymptotically distinct.

Assumption 6 $T_{i}^{0}=\left[T\lambda_{i}^{0}\right]$, where $0<\lambda_{1}^{0}<\cdots <\lambda_{m}^{0}<1$.^7^

To implement 2SLS, it is necessary to specify the reduced form for $x_{t}$. As noted in the introduction, we consider scenarios in which the reduced form for $x_{t}$ is either stable or unstable. In this section, we consider the case in which the reduced form is stable,

(8) $$x_{t}^{\prime }=z_{t}^{\prime }\Delta_{0}+v_{t}^{\prime }$$

where $z_{t}={\left(z_{t,1},z_{t,2},\ldots ,z_{t,q}\right)}^{\prime }$ is a q×1 vector of instruments that is uncorrelated with both $u_{t}$ and $v_{t}$, $\Delta_{0}=\left(\delta_{1,0},\delta_{2,0},\ldots ,\delta_{p_{1},0}\right)$ with dimension $q\times p_{1}$ and each $\delta_{j,0}$ for $j=1,\ldots ,p_{1}$ has dimension q×1. We assume that $z_{t}$ contains $z_{1,t}$. Under the assumption that $E\left[{u_{t}}^{2}|z_{t}\right]=\sigma^{2}$, the optimal IV estimator is the 2SLS estimator.^8^ Our analysis is confined to the 2SLS estimator, although note that the aforementioned conditional homoscedasticity restriction is only imposed in certain parts of the analysis.

We propose the following estimation method. On the first stage, the reduced form for $x_{t}$ is estimated via OLS using (8) and let ${\overset{ˆ}{x}}_{t}$ denote the resulting predicted value for $x_{t}$, that is

(9) $${\overset{ˆ}{x}}_{t}^{\prime }={z_{t}}^{\prime }{\overset{ˆ}{\Delta }}_{T}={z_{t}}^{\prime }{\left(\overset{T}{\underset{t=1}{\sum }}z_{t}{z_{t}}^{\prime }\right)}^{-1}\overset{T}{\underset{t=1}{\sum }}z_{t}{x_{t}}^{\prime }\text{.}$$

In the second stage, we first estimate

(10) $$y_{t}={\overset{ˆ}{x}}_{t}^{\prime }\beta_{x,i}^{*}+z_{1,t}^{\prime }\beta_{z_{1},i}^{*}+{\overset{˜}{u}}_{t}\text{,}\;i=1,\ldots ,m+1;\;t=T_{i-1}+1,\ldots ,T_{i}$$

via OLS for each possible m-partition of the sample, denoted by ${\left\{T_{j}\right\}}_{j=1}^{m}$. We assume the following.

Assumption 7 Eq. (10) is estimated over all partitions $\left(T_{1},\ldots ,T_{m}\right)$ such that $T_{i}-T_{i-1}>\max\left\{q-1,\epsilon T\right\}$ for some ϵ>0 and $\epsilon <\inf_{i}\left(\lambda_{i+1}^{0}-\lambda_{i}^{0}\right)$.

Assumption 7 requires that each segment considered in the minimization contains a positive fraction of the sample asymptotically; in practice ϵ is chosen to be small in the hope that the last part of the assumption is valid. Letting ${\beta_{i}^{*}}^{\prime }={\left({\beta_{x,i}^{*}}^{\prime },{\beta_{z_{1},i}^{*}}^{\prime }\right)}^{\prime }$, for a given m-partition, the estimates of $\beta^{*}={\left({\beta_{1}^{*}}^{\prime },{\beta_{2}^{*}}^{\prime },\ldots ,{\beta_{m+1}^{*}}^{\prime }\right)}^{\prime }$ are obtained by minimizing the sum of squared residuals

(11) $$S_{T}\left(T_{1},\ldots ,T_{m};\;\beta \right)=\overset{m+1}{\underset{i=1}{\sum }}\overset{T_{i}}{\underset{t=T_{i-1}+1}{\sum }}{\left(y_{t}-{\overset{ˆ}{x}}_{t}^{\prime }\beta_{x,i}-z_{1,t}^{\prime }\beta_{z_{1},i}\right)}^{2}$$

with respect to $\beta ={\left({\beta_{1}}^{\prime },{\beta_{2}}^{\prime },\ldots ,{\beta_{m+1}}^{\prime }\right)}^{\prime }$. We denote these estimators by $\overset{ˆ}{\beta }\left({\left\{T_{i}\right\}}_{i=1}^{m}\right)$. The estimates of the break points, $\left({\overset{ˆ}{T}}_{1},\ldots ,{\overset{ˆ}{T}}_{m}\right)$, are defined as

(12) $$\left({\overset{ˆ}{T}}_{1},\ldots ,{\overset{ˆ}{T}}_{m}\right)=\arg\underset{T_{1},\ldots ,T_{m}}{\min}S_{T}\left(T_{1},\ldots ,T_{m};\;\overset{ˆ}{\beta }\left(\overset{m}{\underset{i=1}{\left\{T_{i}\right\}}}\right)\right)$$

where the minimization is taken over all possible partitions, $\left(T_{1},\ldots ,T_{m}\right)$. The 2SLS estimates of the regression parameters, $\overset{ˆ}{\beta }\left({\left\{{\overset{ˆ}{T}}_{i}\right\}}_{i=1}^{m}\right)={\left({{\overset{ˆ}{\beta }}_{1}}^{\prime },{{\overset{ˆ}{\beta }}_{2}}^{\prime },\ldots ,{{\overset{ˆ}{\beta }}_{m+1}}^{\prime }\right)}^{\prime }$, are the regression parameter estimates associated with the estimated partition, ${\left\{\overset{ˆ}{T_{i}}\right\}}_{i=1}^{m}$.

## 4 2SLS based inference when the reduced form is stable

This section is divided into four parts. In part (i), we consider the limiting behaviour of both the break point fraction estimators $\left\{{\overset{ˆ}{\lambda }}_{i}={\overset{ˆ}{T}}_{i}/T\right\}$ and the estimators of the structural parameters, $\overset{ˆ}{\beta }\left({\left\{{\overset{ˆ}{T}}_{i}\right\}}_{i=1}^{m}\right)$. In part (ii), we propose a number of statistics for testing various hypotheses that naturally arise in models with multiple change points. Part (iii) describes how these test statistics can be used to estimate the number of break points.^9^

(i) *Limiting behaviour of the estimators*.

To facilitate the analysis, we impose the following conditions.

Assumption 8 (i) $h_{t}={\left(u_{t},v_{t}^{\prime }\right)}^{\prime }⊗z_{t}$ is an array of real valued n×1 random vectors (where $n=\left(p_{1}+1\right)q$) defined on the probability space (Ω,F,P), $V_{T}=\mathrm{V ar}\left[\sum_{t=1}^{T}h_{t}\right]$ is such that $\mathrm{diag}\left[\xi_{T,1}^{-1},\ldots ,\xi_{T,n}^{-1}\right]=\Xi_{T}^{-1}$ is $O\left(T^{-1}\right)$ where $\Xi_{T}$ is the n×n diagonal matrix with the eigenvalues $\left(\xi_{T,1},\ldots ,\xi_{T,n}\right)$ of $V_{T}$ along the diagonal; (ii) $E\left[h_{t,i}\right]=0$ and, for some $d>2,{\left\|h_{t,i}\right\|}_{d}<\Gamma <\infty$for t=1,2,… and i=1,2,…,n where $h_{t,i}$ is the ith element of $h_{t}$; (iii) $\left\{h_{t,i}\right\}$ is near epoch dependent with respect to $\left\{g_{t}\right\}$ such that ${\left\|h_{t}-E\left[h_{t}|G_{t-m}^{t+m}\right]\right\|}_{2}\leq \nu_{m}$ with $\nu_{m}=O\left(m^{-1/2}\right)$ where $G_{t-m}^{t+m}$ is a sigma-algebra based on $\left(g_{t-m},\ldots ,g_{t+m}\right)$; (iv) $\left\{g_{t}\right\}$ is either ϕ-mixing of size $m^{-d/\left(2\left(d-1\right)\right)}$ or α-mixing of size $m^{-d/\left(d-2\right)}$.

Assumption 9 $\mathrm{rank}\left\{\;\left[\Delta_{0},\;\Pi \right]\;\right\}=p$ where $\Pi^{\prime }=\left[I_{p_{2}},\;0_{p_{2}\times \left(q-p_{2}\right)}\right],I_{a}$ denotes the a×a identity matrix and $0_{a\times b}$ is the a×b null matrix.

Assumption 10 There exists a $0<l_{0}<\min\left\{T_{i}^{0},T-T_{i}^{0}\right\}$ such that for all $l=\left[\xi T\right]>l_{0}$, with $l\leq \min\left\{T_{i}^{0},T-T_{i}^{0}\right\}$, the minimum eigenvalues of $A_{il}=\left(1/l\right)\sum_{t=T_{i}^{0}+1}^{T_{i}^{0}+l}z_{t}{z_{t}}^{\prime }$ and of $A_{il}^{*}=\left(1/l\right)\sum_{t=T_{i}^{0}-l}^{T_{i}^{0}}z_{t}{z_{t}}^{\prime }$ are bounded away from zero in probability for all i=1,…,m+1.

Assumption 11 $T^{-1}\sum_{t=1}^{\left[Tr\right]}z_{t}z_{t}^{\prime }\overset{p}{\to }Q_{ZZ}\left(r\right)$ uniformly in r∈[0,1] where $Q_{ZZ}\left(r\right)$ is pd for any r>0 and strictly increasing in r.

Assumption 8 allows substantial dependence and heterogeneity in $h_{t}$ but at the same time imposes sufficient restrictions to deduce a Central Limit Theorem for $T^{-1/2}\sum_{t=1}^{\left[Tr\right]}h_{t}$; see Wooldridge and White (1988).^10^ This assumption also contains the restrictions that the implicit population moment condition in 2SLS is valid–that is $E\left[z_{t}u_{t}\right]=0$–and the conditional mean of the reduced form is correctly specified. Assumption 9 implies the standard rank condition for identification in IV estimation in the linear regression model^11^ because Assumptions 8(ii), 9 and 11 together imply that

$$T^{-1}\overset{\left[Tr\right]}{\underset{t=1}{\sum }}z_{t}\left[x_{t}^{\prime },z_{1,t}^{\prime }\right]\overset{p}{\to }Q_{ZZ}\left(r\right)\left[\Delta_{0},\;\Pi \right]=Q_{Z,\left[X,Z_{1}\right]}\left(r\right)\;\text{uniformly in }r\in \left[0,1\right]$$

where $Q_{Z,\left[X,Z_{1}\right]}\left(r\right)$ has rank equal to p for any r>0. Assumption 10 requires that there are enough observations near the true break points so that they can be identified and is analogous to the extension proposed by Bai and Perron’s (1998) to their Assumption A2.

We first establish the consistency of the break fraction estimators via a similar argument to Bai and Perron (1998). The proof builds from the following two properties of the error sum of squares on the second stage of the 2SLS estimation: first, since the 2SLS estimators minimize the error sum of squares in (11), it follows that

(13) $$\left(1/T\right)\overset{T}{\underset{t=1}{\sum }}{\overset{ˆ}{u}}_{t}^{2}\leq \left(1/T\right)\overset{T}{\underset{t=1}{\sum }}{\overset{˜}{u}}_{t}^{2}$$

where ${\overset{ˆ}{u}}_{t}=y_{t}-{\overset{ˆ}{x}}_{t}^{\prime }{\overset{ˆ}{\beta }}_{x,j}-z_{1,t}^{\prime }{\overset{ˆ}{\beta }}_{z_{1},j}$ denotes the estimated residuals for $t\in \left[{\overset{ˆ}{T}}_{j-1}+1,{\overset{ˆ}{T}}_{j}\right]$ in the second stage regression of the 2SLS estimation procedure and ${\overset{˜}{u}}_{t}=y_{t}-{\overset{ˆ}{x}}_{t}^{\prime }\beta_{x,i}^{0}-z_{1,t}^{\prime }\beta_{z_{1},i}^{0}$ denotes the corresponding residuals evaluated at the true parameter value for $t\in \left[T_{i-1}^{0}+1,T_{i}^{0}\right]$, and second, using $d_{t}={\overset{˜}{u}}_{t}-{\overset{ˆ}{u}}_{t}={\overset{ˆ}{x}}_{t}^{\prime }\left({\overset{ˆ}{\beta }}_{x,j}-\beta_{x,i}^{0}\right)-z_{1,t}^{\prime }\left({\overset{ˆ}{\beta }}_{z_{1},j}-\beta_{z_{1},i}^{0}\right)$ over $t\in \left[{\overset{ˆ}{T}}_{j-1}+1,{\overset{ˆ}{T}}_{j}\right]\cap \left[T_{i-1}^{0}+1,T_{i}^{0}\right]$, it follows that

(14) $$T^{-1}\overset{T}{\underset{t=1}{\sum }}{\overset{ˆ}{u}}_{t}^{2}=T^{-1}\overset{T}{\underset{t=1}{\sum }}{{\overset{˜}{u}}_{t}}^{2}+T^{-1}\overset{T}{\underset{t=1}{\sum }}{d_{t}}^{2}-2T^{-1}\overset{T}{\underset{t=1}{\sum }}{\overset{˜}{u}}_{t}d_{t}\text{.}$$

Consistency is established by proving that if at least one of the estimated break fractions does not converge in probability to a true break fraction then the results in (13)–(14) contradict each other. This conflict is established using the results in the following lemma.

Lemma 1 *Let* $y_{t}$ *be generated by* (7)*,* $x_{t}$ *be generated by* (8)*,* ${\overset{ˆ}{x}}_{t}$ *be generated by* (9) *and* Assumptions 6–11 *hold.*

(i) $T^{-1}\sum_{t=1}^{T}{\overset{˜}{u}}_{t}d_{t}=o_{p}\left(1\right)$*.*
(ii) *If  for some* j*, then*
  $$\underset{T\to \infty }{lim sup}\;P\left(T^{-1}\overset{T}{\underset{t=1}{\sum }}{d_{t}}^{2}>C\left\{\;{\left\|\Delta_{0}\left(\overset{0}{\underset{x,j}{\beta }}-\overset{0}{\underset{x,j+1}{\beta }}\right)\right\|}^{2}+{\left\|\overset{0}{\underset{z_{1},j}{\beta }}-\overset{0}{\underset{z_{1},j+1}{\beta }}\right\|}^{2}\;\right\}+\xi_{T}\right)>\overset{¯}{\epsilon }$$
  *for some* C>0 *and* $\overset{¯}{\epsilon }>0$*, where* $\xi_{T}=o_{p}\left(1\right)$*.*

Using (13)–(14) and Lemma 1, consistency is established along the lines anticipated above.

Theorem 1 *Let* $y_{t}$ *be generated by* (7)*,* $x_{t}$ *be generated by* (8)*,* ${\overset{ˆ}{x}}_{t}$ *be generated by* (9) *and* Assumptions 6–11 *hold, then* ${\overset{ˆ}{\lambda }}_{j}\;\overset{p}{\to }\lambda_{j}^{0}$ *for all* j=1,2,…,m*.*

The consistency of the 2SLS-based break point estimator is in sharp contrast to the inconsistency of the GMM-based estimator established in Proposition 3. To illustrate the finite sample differences between the estimators, we simulated the behaviour of the 2SLS-based estimator in the one-break model considered in Section 2 and plot the empirical distribution of the break fraction estimator in Fig. 1. In contrast to the diffuse distribution of the GMM-based estimator, the distribution of the 2SLS-based estimator is very concentrated around the true break fraction. For completeness, we also simulated the behaviour of the 2SLS-based estimator in the no-break model when the estimation is performed under the assumption of one break. In this case, the 2SLS-based and GMM-based estimators of the break fraction are similarly diffuse.

To establish asymptotic normality of the parameter estimators, we need to show that the break-fractions are converging faster than the parameters and thus their randomness does not contaminate the limiting distribution of the parameter estimators. This is established in the following result.

Theorem 2 *Let* $y_{t}$ *be generated by* (7)*,* $x_{t}$ *be generated by* (8)*,* ${\overset{ˆ}{x}}_{t}$ *be generated by* (9) *and* Assumptions 6–11 *hold then, for every* η>0*, there exists* C *such that for all large* T*,*

$$P\left(T\left|{\overset{ˆ}{\lambda }}_{j}-\lambda_{j}^{0}\right|>C\right)<\eta \text{,}\;\text{for }j=1,\ldots ,m\text{.}$$

Given Theorem 2, it can be shown that the limiting distribution of the 2SLS parameter estimators is the same as if the break-points are known *a priori*.

Theorem 3 *Let* $y_{t}$ *be generated by* (7)*,* $x_{t}$ *be generated by* (8)*,* ${\overset{ˆ}{x}}_{t}$ *be generated by* (9) *and* Assumptions 6–11 *hold, then*

$$T^{1/2}\left(\;\overset{ˆ}{\beta }\left({\left\{{\overset{ˆ}{T}}_{i}\right\}}_{i=1}^{m}\right)-\beta^{0}\;\right)\Rightarrow N\left(\;0_{p\left(m+1\right)\times 1},\;V_{\beta }\;\right)$$

*where* $\beta^{0}={\left[{\beta_{1}^{0}}^{\prime },{\beta_{2}^{0}}^{\prime },\ldots ,{\beta_{h+1}^{0}}^{\prime }\right]}^{\prime },\beta_{i}^{0}={\left[{\beta_{x,i}^{0}}^{\prime },{\beta_{z_{1},i}^{0}}^{\prime }\right]}^{\prime }$*,*

$$V_{\beta }=\left(\begin{array}{ccc} V_{\beta }^{\left(1,1\right)} & \cdots & V_{\beta }^{\left(1,m+1\right)}\\ \vdots & \ddots & \vdots \\ V_{\beta }^{\left(m+1,1\right)} & \cdots & V_{\beta }^{\left(m+1,m+1\right)} \end{array}\right)$$

$$V_{i,i}=A_{i}\left\{C_{i}V_{i}C_{i}^{\prime }-E_{i}D_{i}V_{i}C_{i}^{\prime }-C_{i}V_{i}D_{i}^{\prime }E_{i}^{\prime }+E_{i}D_{i}VD_{i}^{\prime }E_{i}^{\prime }\right\}A_{i}^{\prime }$$

$$V_{i,j}=A_{i}E_{i}D_{i}VD_{j}^{\prime }E_{j}^{\prime }A_{j}^{\prime }-A_{i}E_{i}D_{i}V_{j}C_{j}^{\prime }A_{j}^{\prime }-A_{i}C_{i}V_{i}D_{j}^{\prime }E_{j}^{\prime }A_{j}^{\prime }\text{,}\;\text{for }i\neq j$$

$$A_{i}={\left[\Upsilon_{0}^{\prime }Q_{i}\Upsilon_{0}\right]}^{-1}\Upsilon_{0}^{\prime }\text{,}\;E_{i}=Q_{i}Q_{ZZ}{\left(1\right)}^{-1}\text{,}\;\text{for }i=1,2,\ldots ,m+1$$

$$\Upsilon_{0}=\left[\Delta_{0},\Pi \right]\text{,}\;C_{i}=\left[I_{q},{\beta_{x,i}^{0}}^{\prime }⊗I_{q}\right]\text{,}\;D_{i}=\left[0_{q\times q},{\beta_{x,i}^{0}}^{\prime }⊗I_{q}\right]$$

$$Q_{i}=Q_{ZZ}\left(\lambda_{i}^{0}\right)-Q_{ZZ}\left(\lambda_{i-1}^{0}\right)\text{,}\;V_{i}=\mathrm{V ar}\left[T^{-1/2}\overset{\left[\lambda_{i}T\right]}{\underset{t=\left[\lambda_{i-1}T\right]+1}{\sum }}h_{t}\right]\text{,}\;V=\mathrm{V ar}\left[T^{-1/2}\overset{T}{\underset{t=1}{\sum }}h_{t}\right]\text{.}$$

Note that $V_{\left(i,j\right)}$ is non-zero in general because the first stage regression pools observations across regimes and this creates a connection between the 2SLS estimators from different regimes. A consistent estimator of this variance can be constructed in a straightforward fashion by replacing $\Delta_{0},\beta^{0},Q_{ZZ}\left(r\right),Q_{i},V_{i}$ and V by respectively ${\overset{ˆ}{\Delta }}_{T},\overset{ˆ}{\beta }\left({\left\{{\overset{ˆ}{T}}_{i}\right\}}_{i=1}^{m}\right),T^{-1}\sum_{t=1}^{\left[Tr\right]}z_{t}z_{t}^{\prime }$, and HAC estimators of $V_{i}$ and V based on ${\overset{ˆ}{u}}_{t}=y_{t}-{\left(x_{t}^{\prime },z_{1,t}^{\prime }\right)}^{\prime }\overset{ˆ}{\beta }\left({\left\{{\overset{ˆ}{T}}_{i}\right\}}_{i=1}^{m}\right)$ and ${\overset{ˆ}{v}}_{t}=x_{t}-{\overset{ˆ}{\Delta }}_{T}^{\prime }z_{t}$.^12^

(ii) *Hypothesis testing*: In this sub-section, we consider three types of hypothesis tests that naturally arise in this class of models: (a) $H_{0}:\;m=0$ vs. $H_{1}:\;m=k$; (b) $H_{0}:\;m=0$ vs. $H_{1}:\;m\leq K$; (c) $H_{0}:\;m=\ell$ vs. $H_{1}:\;m=\ell +1$. We consider F-type tests and Wald-type tests for each. To develop both types of tests, we need to impose additional assumptions on the instrument cross-product matrix and long run variance of the instrument-error product vector, $h_{t}$. The exact nature of the assumptions depends on the type of statistic and the null hypothesis.

We begin by considering F-type statistics for $H_{0}:\;m=0$. For this scenario, we impose the following two assumptions.

Assumption 12 $T^{-1}\sum_{t=1}^{\left[Tr\right]}z_{t}z_{t}^{\prime }\overset{p}{\to }rQ_{ZZ}$ uniformly in r∈[0,1] where $Q_{ZZ}$ is a pd matrix of constants.

Assumption 13 Let $b_{t}={\left(u_{t},v_{t}^{\prime }\right)}^{\prime }$ and $F=\sigma -\mathrm{field}\left\{\ldots ,z_{t-1},z_{t},\ldots ,b_{t-2},b_{t-1}\right\}$. $b_{t}$ is a martingale difference relative to $\left\{F_{t}\right\}$ and $\sup_{t}E\left[{\left\|b_{t}\right\|}^{4}\right]<\infty$ and the conditional variance of the errors is independent of t, that is $\mathrm{V ar}\left[u_{t},v_{t}^{\prime }|z_{t}\right]=\Omega$, a constant pd matrix with the conditional variances of $u_{t}$ and $v_{t}$ denoted by $\sigma^{2}$ and Σ respectively, and the conditional covariance between $u_{t}$ and $v_{t}$ denoted by $\gamma^{\prime }$.

The restrictions in Assumptions 12 and 13 are analogous to those imposed by Bai and Perron (1998) in their Assumptions A8 and A9 which underpin their analysis of various F-statistics for testing for multiple breaks within the OLS framework.

The sup-F type test of $H_{0}:\;m=0$ vs. $H_{A}:\;m=1$ has been considered by Andrews (1993). The results below are the 2SLS extensions of Bai and Perron’s (1998) tests.

The sup-F type test statistic can be defined as follows. Let $\left(T_{1},\ldots ,T_{k}\right)$ be a partition such that $T_{i}=\left[T\lambda_{i}\right]\;\left(i=1,\ldots ,k\right)$. Define

(15) $$F_{T}\left(\lambda_{1},\ldots ,\lambda_{k};p\right)=\left\{\;\frac{T-\left(k+1\right)p}{kp}\;\right\}\left\{\;\frac{{\mathrm{SSR}}_{0}-{\mathrm{SSR}}_{k}}{{\mathrm{SSR}}_{k}}\;\right\}$$

where ${\mathrm{SSR}}_{0}$ and ${\mathrm{SSR}}_{k}$ are the sum of squared residuals based on fitted $x_{t}$ under null and alternative hypotheses, respectively. Recall from Assumption 7 that the minimization is performed over partitions which are asymptotically large and the size of the partitions is controlled by ϵ, a non-negative constant. Accordingly, we define

$$\Lambda_{\epsilon }=\left\{\left(\lambda_{1},\ldots ,\lambda_{k}\right):\left|\lambda_{i+1}-\lambda_{i}\right|\geq \epsilon ,\lambda_{1}\geq \epsilon ,\lambda_{k}\leq 1-\epsilon \right\}\text{.}$$

Finally, the sup-F test statistic is defined as

(16) $$\mathrm{Sup}\text{-}F_{T}\left(k;p\right)={\mathrm{Sup}}_{\left(\lambda_{1},\ldots ,\lambda_{k}\right)\in \Lambda_{\epsilon }}F_{T}\left(\lambda_{1},\ldots ,\lambda_{k};p\right)\text{.}$$

Theorem 4 *If the data are generated by* (7)*–*(8) *with* $m=0,{\overset{ˆ}{x}}_{t}$ *is generated by* (9) *and* Assumptions 6–13 *hold then*^13^$\mathrm{Sup}\text{-}F_{T}\left(k;p\right)\Rightarrow \mathrm{Sup}\text{-}F_{k,p}\equiv {\mathrm{Sup}}_{\left(\lambda_{1},\ldots ,\lambda_{k}\right)\in \Lambda_{\epsilon }}F\left(\lambda_{1},\ldots ,\lambda_{k};p\right)$ *where*

$$F\left(\lambda_{1},\ldots ,\lambda_{k};p\right)\equiv \frac{1}{kp}\overset{k}{\underset{i=1}{\sum }}\frac{{\left\|\lambda_{i+1}W_{i}-\lambda_{i}W_{i+1}\right\|}^{2}}{\lambda_{i}\lambda_{i+1}\left(\lambda_{i+1}-\lambda_{i}\right)}$$

*where* k *is the number of break points under the alternative hypothesis, and* $W_{i}\equiv B_{p}\left(\lambda_{i}\right)$*, where* $B_{p}\left(\cdot \right)$ *is a* p×1 *vector of independent standard Brownian motions.*

We note that the limiting distribution in Theorem 4 is exactly the same as the one (Bai and Perron, 1998) obtain for the sup-F test based on OLS estimators when the regressors are exogenous. Percentiles for this distribution can be found in Bai and Perron (1998, Table I) for ϵ=0.05 and in Bai and Perron (2003) for other values of ϵ.

The $\mathrm{Sup}\text{-}F_{T}\left(k;p\right)$ statistic is used to test the null hypothesis of structural stability against the k-break model, and so is designed for the case in which a particular choice of k is of interest. In many circumstances, a researcher is unlikely to know *a priori* the appropriate choice of k for the alternative hypothesis. To circumvent this problem, Bai and Perron (1998) propose so called “Double Maximum tests” that combine information from the $\mathrm{Sup}\text{-}F_{T}\left(k;p\right)$ statistics for different values of k running from one to some ceiling K. We consider here only the following example of Double Maximum test,^14^

(17) $$\mathrm{UDmax}\;F_{T}\left(K;p\right)=\underset{1\leq k\leq K}{\max}\underset{\left(\lambda_{1},\ldots ,\lambda_{k}\right)\in \Lambda_{\epsilon }}{\sup}F_{T}\left(\lambda_{1},\ldots ,\lambda_{k};p\right)\text{.}$$

The limiting distribution of this statistic follows directly from Theorem 4.

Corollary 1 *Under the conditions of* Theorem 4*, it follows that*

$$\mathrm{UDmax}\;F_{T}\left(K;p\right)⟹\underset{1\leq k\leq K}{\max}\left\{\mathrm{Sup}\text{-}F_{k,p}\;\right\}\text{.}$$

Critical values for the limiting distribution in Corollary 1 are presented in Bai and Perron (1998, Table 1) for ϵ=0.05 and in Bai and Perron (2003) for other values of ϵ.

The $\mathrm{Sup}\text{-}F_{T}\left(k;p\right)$ and $\mathrm{UDmax}\;F_{T}\left(K;p\right)$ statistics are used to test the null hypothesis of no breaks. It is also of interest to develop statistics for testing the null hypothesis of ℓ breaks against the alternative of ℓ+1 breaks. For this scenario, we relax Assumptions 12 and 13 as follows.

Assumption 14 $T^{-1}\sum_{t=\left[Tr\right]+1}^{\left[Ts\right]}z_{t}z_{t}^{\prime }\overset{p}{\to }\left(r-s\right)Q_{ZZ}^{\left(i\right)}$, where $\lambda_{i-1}^{0}\leq r<s\leq \lambda_{i}^{0}$, uniformly in r×s and $Q_{ZZ}^{\left(i\right)}$ is a pd matrix of constants, not necessarily the same for all i.

Assumption 15 $\mathrm{V ar}\left[{\left(u_{t},v_{t}^{\prime }\right)}^{\prime }|z_{t}\;\right]=\Omega_{i}$, a pd matrix of constants, for $t\in \left(\left[T\lambda_{i-1}^{0}\right]+1,\left[T\lambda_{i}^{0}\right]\right)$ and $\sigma_{i}^{2},\Sigma_{i}$ and $\gamma_{i}$ denote the sub-matrices of $\Omega_{i}$ relating respectively to the conditional variance of $u_{t}$, the conditional variance of $v_{t}$ and the conditional covariance of $v_{t}$ and $u_{t}$.

Notice that Assumption 14 only imposes homogeneity of the instrument cross-product matrix within each regime and Assumption 15 allows the conditional error variance to change at the same time as the structural parameters.

Following Bai and Perron (1998), a suitable statistic can be constructed as follows. For the model with ℓ breaks, the estimated break points, denoted by ${\overset{ˆ}{T}}_{1},\ldots ,{\overset{ˆ}{T}}_{\ell }$, are obtained by a global minimization of the sum of the squared residuals as in (12). For the model with ℓ+1 breaks, ℓ breaks are fixed at ${\overset{ˆ}{T}}_{1},\ldots ,{\overset{ˆ}{T}}_{\ell }$ and then the location of the (ℓ+1)th break is chosen by minimizing the residual sum of squares. The test statistic is given in Box I. The following theorem gives the limiting distribution of this statistic under the null hypothesis of ℓ breaks.

Theorem 5 *If the data are generated by* (7)*–*(8) *with* $m=\ell ,{\overset{ˆ}{x}}_{t}$ *is generated by* (9) *and* Assumptions 6–11, 14 and 15 *hold then* $\lim_{T\to \infty }P\left(F_{T}\left(\ell +1|\ell \right)\leq x\right)=G_{p,\eta }{\left(x\right)}^{\ell +1}$ *where* $G_{p,\eta }\left(x\right)$ *is the distribution function of* $\sup_{\eta \leq \mu \leq 1-\eta }{\left\|W\left(\mu \right)-\mu W\left(1\right)\right\|}^{2}/\mu \left(1-\mu \right)$ *and* $W\left(\mu \right)\equiv B_{p}\left(\mu \right)$*.*

Once again, the limiting behaviour of the test statistic is the same as that of the analogous statistic proposed by Bai and Perron (1998) for the OLS case. Critical values can be found in Bai and Perron (1998, Table II) for the case with η=0.05 and in Bai and Perron (2003) for other values of η.

The restriction on the errors in Assumptions 13 or 15 is satisfied in some applications but rules out many other cases of interest. Unfortunately, it is not simple to modify the F-type statistics to handle more general error processes, and so we also consider statistics based on the Wald principle. For this part of the analysis, the errors are only restricted to satisfy the following.

Assumption 16 Define $V_{T}\left(r\right)=\mathrm{V ar}\left[T^{-1/2}\sum_{t=1}^{\left[Tr\right]}h_{t}\right]$ then $V_{T}\left(r\right)\to rV$ uniformly in r∈[0,1] where V is a pd matrix.

Notice that this assumption allows for serial correlation and conditional heteroscedasticity in $h_{t}$ and, thus, in the errors $u_{t}$ and $v_{t}$. However, note that we maintain Assumption 8(ii) which includes $E\left[h_{t}\right]=0$, and so if the errors are serially correlated then, in general, $z_{t}$ must exclude lagged values of $y_{t}$ or $x_{t}$.

To develop the Wald test of $H_{0}:\;m=0$ versus $H_{1}:\;m=k$, we restate the null and alternative hypotheses in terms of linear restrictions on the parameters. Accordingly, we define $R_{k}={\overset{˜}{R}}_{k}⊗I_{p}$ where ${\overset{˜}{R}}_{k}$ is the k×(k+1) matrix whose i−jth element, ${\overset{˜}{R}}_{k}\left(i,j\right)$, is given by: ${\overset{˜}{R}}_{k}\left(i,i\right)=1,{\overset{˜}{R}}_{k}\left(i,i+1\right)=-1,{\overset{˜}{R}}_{k}\left(i,j\right)=0$ for i=1,2,…,k and j≠i,i+1. The null and alternative can then be equivalently stated as: $H_{0}:\;R_{k}\beta^{0}\left(k\right)=0$ versus $H_{1}:\;R_{k}\beta^{0}\left(k\right)\neq 0$ where $\beta^{0}\left(k\right)={\left({\beta_{1}^{0}}^{\prime },{\beta_{2}^{0}}^{\prime },\ldots ,{\beta_{k}^{0}}^{\prime }\right)}^{\prime }$. The test statistic is then:

(19) $$\mathrm{Sup}\text{-}{\mathrm{Wald}}_{T}\left(k,p\right)=\underset{\left(\lambda_{1},\lambda_{2},\ldots ,\lambda_{k}\right)\in \Lambda_{\epsilon }}{\sup}T\overset{ˆ}{\beta }{\left({\overset{¯}{T}}_{k}\right)}^{\prime }\overset{\prime }{\underset{k}{R}}{\left[R_{k}{\overset{ˆ}{V}}_{W}\left({\overset{¯}{T}}_{k}\right)R_{k}^{\prime }\right]}^{-1}R_{k}\overset{ˆ}{\beta }\left({\overset{¯}{T}}_{k}\right)$$

where $\overset{ˆ}{\beta }\left({\overset{¯}{T}}_{k}\right)$ is the 2SLS estimator of $\beta^{0}\left(k\right)$ based on k-partition ${\overset{¯}{T}}_{k}=\left(\left[\lambda_{1}T\right],\ldots ,\left[\lambda_{k}T\right]\right),{\overset{ˆ}{V}}_{W}\left({\overset{¯}{T}}_{k}\right)=\mathrm{diag}\left[{\overset{ˆ}{V}}_{W}^{\left(1\right)}\left({\overset{¯}{T}}_{k}\right),\ldots ,{\overset{ˆ}{V}}_{W}^{\left(k+1\right)}\left({\overset{¯}{T}}_{k}\right)\right]$,

$${\overset{ˆ}{V}}_{W}^{\left(i\right)}\left({\overset{¯}{T}}_{k}\right)={\left\{T^{-1}\overset{\left[\lambda_{i}T\right]}{\underset{t=\left[\lambda_{i-1}T\right]+1}{\sum }}w_{t}w_{t}^{\prime }\right\}}^{-1}{\overset{ˆ}{H}}_{i}\left({\overset{¯}{T}}_{k}\right){\left\{T^{-1}\overset{\left[\lambda_{i}T\right]}{\underset{t=\left[\lambda_{i-1}T\right]+1}{\sum }}w_{t}w_{t}^{\prime }\right\}}^{-1}\text{,}$$

where ${\overset{ˆ}{H}}_{i}\left({\overset{¯}{T}}_{k}\right)$ is a consistent estimator of $H_{i}=\lim_{T\to \infty }\mathrm{V ar}\left[T^{-1/2}\sum_{t=\left[\lambda_{i-1}T\right]+1}^{\left[\lambda_{i}T\right]}\Upsilon_{0}^{\prime }z_{t}\left\{u_{t}+v_{t}^{\prime }\beta_{x,i}^{0}\left(k\right)\right\}\right],w_{t}={\left({\overset{ˆ}{x}}_{t}^{\prime },z_{1,t}^{\prime }\right)}^{\prime }$ and ${\overset{ˆ}{H}}_{i}\left({\overset{¯}{T}}_{k}\right)$ can be constructed using a HAC estimator based on ${\overset{ˆ}{\Upsilon }}_{T}^{\prime }z_{t}\left\{{\overset{ˆ}{u}}_{t}+{\overset{ˆ}{v}}_{t}^{\prime }{\overset{ˆ}{\beta }}_{x}\right\}$, with ${\overset{ˆ}{\Upsilon }}_{T}=\left[{\overset{ˆ}{\Delta }}_{T},\Pi \right],{\overset{ˆ}{u}}_{t}=y_{t}-x_{t}^{\prime }{\overset{ˆ}{\beta }}_{x}-z_{1,t}^{\prime }{\overset{ˆ}{\beta }}_{z_{1}}$ and ${\overset{ˆ}{v}}_{t}=x_{t}-{\overset{ˆ}{\Delta }}_{T}^{\prime }z_{t}$, and $\left\{{\overset{ˆ}{\beta }}_{x},{\overset{ˆ}{\beta }}_{z_{1}}\right\}$ are the 2SLS estimators of the coefficients on x and $z_{1}$ obtained under the null hypothesis of no breaks.

An important feature of ${\overset{ˆ}{V}}_{W}^{\left(i\right)}\left({\overset{¯}{T}}_{k}\right)$ is that it ignores the dependence across sub-samples noted in the discussion following Theorem 3. The reason for this is as follows: under Assumption 12, $T^{1/2}R_{k}\overset{ˆ}{\beta }\left({\overset{¯}{T}}_{k}\right)$ does not involve the terms that create the dependence between estimators from different regimes. The following theorem gives the limiting distribution of the sup-Wald test.

Theorem 6 *If the data are generated by* (7)*–*(8) *with* $m=0,{\overset{ˆ}{x}}_{t}$ *is generated by* (9) *and* Assumptions 6–12 and 16 *hold then*

$$\mathrm{Sup}\text{-}{\mathrm{Wald}}_{T}\left(k,p\right)\Rightarrow \overset{k}{\underset{i=1}{\sum }}\frac{{\left\|\lambda_{i+1}W_{i}-\lambda_{i}W_{i+1}\right\|}^{2}}{\lambda_{i}\lambda_{i+1}\left(\lambda_{i+1}-\lambda_{i}\right)}$$

*where* k *is the number of break points under the alternative hypothesis.*

A comparison of Theorems 4 and 6 indicates that $\left(1/kp\right)\mathrm{Sup}\text{-}{\mathrm{Wald}}_{T}\left(k,p\right)$ has the same limiting distribution as $\mathrm{Sup}\text{-}F_{T}\left(k;p\right)$.

To test $H_{0}:\;m=0$ vs. $H_{1}:\;m\leq K$, we define analogously to $\mathrm{UDmax}\;F_{T}\left(K;p\right)$ the statistic:

$${\mathrm{UDmaxWald}}_{T}\left(K;p\right)=\underset{1\leq k\leq K}{\max}\left(1/kp\right)\mathrm{Sup}\text{-}{\mathrm{Wald}}_{T}\left(k,p\right)$$

Corollary 2 *Under the conditions of* Theorem 6*, it follows that*

$${\mathrm{UDmaxWald}}_{T}\left(K;p\right)⟹\underset{1\leq k\leq K}{\max}\left\{\mathrm{Sup}\text{-}F_{k,p}\;\right\}\text{.}$$

The limiting distribution of ${\mathrm{UDmax Wald}}_{T}\left(K;p\right)$ is identical to that for $\mathrm{UDmax}\;F_{T}\left(K;p\right)$ given in Corollary 1. Notice that the test statistic involves $\mathrm{Sup}\text{-}{\mathrm{Wald}}_{T}\left(k,p\right)$ divided by kp; this scaling is employed because the limiting distribution of $\mathrm{Sup}\text{-}{\mathrm{Wald}}_{T}\left(k,p\right)$ is increasing in k for fixed p and so, without the scaling, the test statistic $\max_{1\leq k\leq K}\mathrm{Sup}\text{-}{\mathrm{Wald}}_{T}\left(k,p\right)$ would be equivalent to testing 0 versus K breaks.

To test $H_{0}:\;m=\ell$ vs. $H_{1}:\;m=\ell +1$ via the Wald principle, we proceed as follows. Under the null hypothesis, there are ℓ breaks and hence ℓ+1 regimes within which the parameters are constant; under the alternative one of these regimes contains an additional break point at which the parameters change. We can therefore test the null hypothesis by calculating, for each of the ℓ+1 regimes, the Wald statistic for a single break and then basing inference on the supremum of these ℓ+1 statistics. Therefore, the test statistic is

$${\mathrm{Wald}}_{T}\left(\ell +1|\ell \right)=\underset{1\leq i\leq \ell +1}{\max}\underset{\tau \in \Lambda_{i,\eta }}{\sup}{\mathrm{Wald}}_{T,\ell }\left(\tau ,i;p\right)$$

where ${\mathrm{Wald}}_{T,\ell }\left(\tau ,i;p\right)$ is defined to be the Wald statistic for a single break at $t={\overset{ˆ}{T}}_{i-1}+\tau$ based on the sub-sample $\Lambda_{i,\eta }$, that is

$${\mathrm{Wald}}_{T,\ell }\left(\tau ,i;p\right)=T\overset{ˆ}{\beta }{\left(\tau ;i\right)}^{\prime }R_{1}^{\prime }{\left[R_{1}{\overset{ˆ}{V}}_{W}\left(\tau ;i\right)R_{1}^{\prime }\right]}^{-1}R_{1}\overset{ˆ}{\beta }\left(\tau ;i\right)$$

where $\overset{ˆ}{\beta }\left(\tau ;i\right)={\left[{\overset{ˆ}{\beta }}_{1}^{\prime }\left(\tau ;i\right),{\overset{ˆ}{\beta }}_{2}^{\prime }\left(\tau ;i\right)\right]}^{\prime },{\overset{ˆ}{\beta }}_{1}\left(\tau ;i\right)$ are the 2SLS estimators of the parameters in the structural equation based on observations $S_{1}\left(\tau ,i\right)=\left\{{\overset{ˆ}{T}}_{i-1}+1,{\overset{ˆ}{T}}_{i-1}+2,\ldots ,{\overset{ˆ}{T}}_{i-1}+\tau \right\},{\overset{ˆ}{\beta }}_{2}\left(\tau ;i\right)$ are the 2SLS estimators of the parameters in the structural equation based on observations $S_{2}\left(\tau ,i\right)=\left\{{\overset{ˆ}{T}}_{i-1}+\tau +1,\ldots ,{\overset{ˆ}{T}}_{i}\right\},{\overset{ˆ}{V}}_{W}\left(\tau ;i\right)=\mathrm{diag}\left[{\overset{ˆ}{V}}_{W}^{\left(1\right)}\left(\tau ;i\right),{\overset{ˆ}{V}}_{W}^{\left(2\right)}\left(\tau ;i\right)\right]$,

$${\overset{ˆ}{V}}_{W}^{\left(j\right)}\left(\tau ;i\right)={\left\{T^{-1}\underset{S_{j}\left(\tau ,i\right)}{\sum }w_{t}\overset{\prime }{\underset{t}{w}}\right\}}^{-1}{\overset{ˆ}{H}}_{i}^{\left(j\right)}\left({\overset{¯}{T}}_{k}\right){\left\{T^{-1}\underset{S_{j}\left(\tau ,i\right)}{\sum }w_{t}\overset{\prime }{\underset{t}{w}}\right\}}^{-1}\text{,}$$

$\sum_{S_{j}\left(\tau ,i\right)}$ denotes summation over $t\in S_{j}\left(\tau ,i\right)$ for j=1,2, and ${\overset{ˆ}{H}}_{i}^{\left(j\right)}$ is a consistent estimator of $\lim_{T\to \infty }\mathrm{V ar}\left[T^{-1/2}\sum_{S_{j}\left(\tau ,i\right)}\Upsilon_{0}^{\prime }z_{t}\left\{u_{t}+v_{t}^{\prime }\beta_{x,i}^{0}\right\}\right]$. ${\overset{ˆ}{H}}_{i}^{\left(j\right)}$ can be constructed using a HAC estimator based on ${\overset{ˆ}{\Upsilon }}_{T}^{\prime }z_{t}\left\{{\overset{ˆ}{u}}_{t}+{\overset{ˆ}{v}}_{t}^{\prime }{\overset{ˆ}{\beta }}_{x,i}\right\},{\overset{ˆ}{u}}_{t}=y_{t}-x_{t}^{\prime }{\overset{ˆ}{\beta }}_{x,i}-z_{1,t}^{\prime }{\overset{ˆ}{\beta }}_{z_{1},i}$ and ${\overset{ˆ}{v}}_{t}=x_{t}-{\overset{ˆ}{\Delta }}_{T}^{\prime }z_{t}$; such an estimator is consistent under $H_{0}$. The following theorem gives the limiting distribution of ${\mathrm{Wald}}_{T}\left(\ell +1|\ell \right)$.

Theorem 7 *If the data are generated by* (7)*–*(8) *with* $m=\ell ,{\overset{ˆ}{x}}_{t}$ *is generated by* (9) *and* Assumptions 6–11, 14 and 16 *hold then* $\lim_{T\to \infty }P\left({\mathrm{Wald}}_{T}\left(\ell +1|\ell \right)\leq x\right)=G_{p,\eta }{\left(x\right)}^{\ell +1}$ *where* $G_{p,\eta }\left(x\right)$ *is defined in* Theorem 5*.*

(iii) *Estimation of the number of breaks*.

Following Bai and Perron (1998), the statistics described in this section can be used to determine the estimated number of break points, ${\overset{ˆ}{m}}_{T}$ say, via the following sequential strategy (for illustrative purposes we describe the method in terms of the F-type statistics but the same strategy can also be used with the Wald-type tests). On the first step, use either $\mathrm{Sup}\text{-}F_{T}\left(1;p\right)$ or $\mathrm{UDmax}\;F_{T}\left(K,p\right)$ to test the null hypothesis that there are no breaks. If this null is not rejected then ${\overset{ˆ}{m}}_{T}=0$; else proceed to the next step. On the second step $F_{T}\left(2|1\right)$ is used to test the null hypothesis that there is only one break against the alternative hypothesis of two breaks. If $F_{T}\left(2|1\right)$ is insignificant then ${\overset{ˆ}{m}}_{T}=1$; else proceed to the next step. On the ℓth step $F_{T}\left(\ell +1|\ell \right)$ is used to test the null hypothesis that there are ℓ breaks against the alternative hypothesis of ℓ+1 breaks. If $F_{T}\left(\ell +1|\ell \right)$ is insignificant then ${\overset{ˆ}{m}}_{T}=\ell$; else proceed to the next step. This sequence is continued until some preset ceiling for the number of breaks, L say, is reached. If all statistics in the sequence are significant then the conclusion is that there are at least L breaks.

(iv) *Finite sample performance*.

In this sub-section, we evaluate the finite sample performance of the methods described in this section. We consider in order models with one, two and no breaks.

*One break model*: We return to the model used in the simulations reported in Section 2, except this time, we report results for q=4,8 and T=120,240,480. Recall that Fig. 1 contains a plot of the empirical distribution of ${\overset{ˆ}{\lambda }}_{1}$ for the estimation with m=1. It can be seen that this distribution is collapsing towards a point mass of one at $\lambda_{1}^{0}=0.5$ as T increases in line with Theorem 1. Table 1 reports the coverage probabilities of the 2SLS estimator of $\beta_{i}^{0}$ based on the asymptotic distribution in Theorem 3.^15^ As can be seen, the coverage is close to the nominal levels. Table 2 reports the rejection frequencies for the F-type and Wald-type statistics. Specifically, we report values for: (i) the $\mathrm{Sup}\text{-}F_{T}\left(k;1\right)$ and $\mathrm{Sup}\text{-}{\mathrm{Wald}}_{T}\left(k;1\right)$ statistics with k=1,2, and the ${\mathrm{UDmaxF}}_{T}\left(5,1\right)$ and ${\mathrm{UDmaxWald}}_{T}\left(5,1\right)$; note that the null hypothesis is incorrect for these statistics; (ii) the $F_{T}\left(\ell +1|\ell \right)$ and ${\mathrm{Wald}}_{T}\left(\ell +1|\ell \right)$ statistics for ℓ=1,2,3; note that the null is correct for ℓ=1 but involves more than the true number of breaks for ℓ>1. It can be seen that the sup-type and UDmax-type statistics correctly reject the null with probability one. The $F_{T}\left(2|1\right)$ and ${\mathrm{Wald}}_{T}\left(2|1\right)$ statistics are slightly undersized but close to their nominal size; if ℓ exceeds the true number of breaks then both $F_{T}\left(\ell +1|\ell \right)$ and ${\mathrm{Wald}}_{T}\left(\ell +1|\ell \right)$ reject very rarely. Table 3 reports the empirical distribution of the estimated number of break points obtained using the sequential strategy in (iii) above with L=5. We first note that the results are identical whether the $\mathrm{Sup}\text{-}F_{T}\left(1;p\right)\;\left(\mathrm{Sup}\text{-}{\mathrm{Wald}}_{T}\left(1;p\right)\right)$ or the ${\mathrm{UDmaxF}}_{T}\left(5,1\right)\;\left({\mathrm{UDmaxWald}}_{T}\left(5,1\right)\right)$ statistic is used on the first step (and so we only report the latter) although there are some slight differences if the F-type or Wald-type statistic is used. As can be seen, the method estimates the true number with probability never less than 94.6% and never underfits. Overfitting is confined to picking two breaks (one too many) with a three break model being picked only once in some designs; more than three breaks are never selected.

*Two break model*: The data generation process for the structural equation is

$$y_{t}={\left[1,\;x_{t}\right]}^{\prime }\beta_{i}^{0}+u_{t}\text{,}$$

where $\beta_{i}^{0}={\left(-1\right)}^{i+1}\left[1,0.1\right]$ for $t=\left[\left[\lambda_{i-1}T\right]+1,\left[\lambda_{i}T\right]\right],\lambda_{1}=1/3,\lambda_{2}=2/3$. All other aspects of the design are the same as the one break model.

**Fig. 3:**
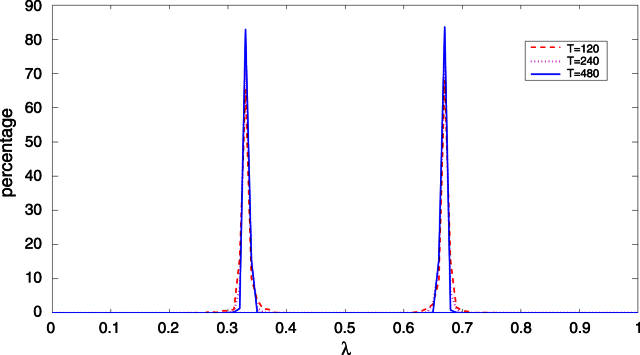
Distribution of estimated break fractions in the two break model.

Fig. 3 contains plots of the empirical distribution of the break fraction estimators for the estimation with m=2. It can be seen that the distribution for each break fraction estimator is collapsing towards a point mass of one at the appropriate true parameter value (0.33 or 0.66) as T increases in line with Theorem 1. Table 4 reports the coverage probabilities of the 2SLS estimator of $\beta_{i}^{0}$ based on the asymptotic distribution in Theorem 3. As in the one break model, the coverage probabilities are very close to the nominal levels. Table 5 reports the rejection frequencies for the test statistics. As in the one break model, the null hypothesis is incorrect for the $\mathrm{Sup}\text{-}F_{T}\left(k;1\right)$ and $\mathrm{Sup}\text{-}{\mathrm{Wald}}_{T}\left(k;1\right)$ statistics with k=1,2, and the ${\mathrm{UDmaxF}}_{T}\left(5,1\right)$ and ${\mathrm{UDmaxWald}}_{T}\left(5,1\right)$ statistics. However, this time for $F_{T}\left(\ell +1|\ell \right)$ and ${\mathrm{Wald}}_{T}\left(\ell +1|\ell \right)$, the null is incorrect for ℓ=1 but correct for ℓ=2. It can be seen that the sup-type and UDmax-type statistics, $F_{T}\left(2|1\right)$ and ${\mathrm{Wald}}_{T}\left(2|1\right)$ correctly reject the null with probability one. The $F_{T}\left(3|2\right)$ and ${\mathrm{Wald}}_{T}\left(3|2\right)$ statistics are slightly undersized but close to their nominal size. Table 6 reports the empirical distribution of the estimated number of break points obtained using the sequential strategy in (iii) above with L=5.^16^ As can be seen, the method estimates the true number with probability never less than 94.7% and never underfits. Overfitting is confined to picking three breaks (one too many).

*No break model*: Data are generated from (5) with $\beta_{1}^{0}=\beta_{2}^{0}=\left[1,0.1\right]$. All other aspects of the design are the same as the one break model. Table 7 contains the empirical rejection frequencies of the test statistics: note that the null hypothesis is correct for all statistics except $F_{T}\left(\ell +1|\ell \right)$ and ${\mathrm{Wald}}_{T}\left(\ell +1|\ell \right)$ for which the null involves the assumption of (too many) breaks. It can be seen that the sup-type and UDmax-type tests based on the F statistic are close to their nominal size but the corresponding tests based on the Wald statistic tend to be slightly over-sized. Interestingly the sup-type Wald tests are closer to their nominal size than the UDmax-Wald test. This difference has implications for the estimation of the number of breaks: the sequential strategy based on F-statistics selects the true value of m at least 94% of the time, but the strategy based on the Wald statistics only does so at least 90% of the time (see Table 8).

## 5 Unstable reduced form: model and estimation

We now consider the case in which the reduced form for $x_{t}$ is

(20) $$x_{t}^{\prime }=z_{t}^{\prime }\Delta_{0}^{\left(i\right)}+v_{t}^{\prime }\text{,}\;i=1,2,\ldots ,h+1,\;t=T_{i-1}^{*}+1,\ldots ,T_{i}^{*}$$

where $T_{0}^{*}=0$ and $T_{h+1}^{*}=T$. The points $\left\{T_{i}^{*}\right\}$ are assumed to be generated as follows.

Assumption 17 $T_{i}^{*}=\left[T\pi_{i}^{0}\right]$, where $0<\pi_{1}^{0}<\cdots <\pi_{h}^{0}<1$.

Note that the break fractions $\left\{\pi_{i}^{0}\right\}$ may or may not coincide with $\left\{\lambda_{i}^{0}\right\}$. Let $\pi^{0}={\left[\pi_{1}^{0},\pi_{2}^{0},\ldots ,\pi_{h}^{0}\right]}^{\prime }$. Also note that (20) can be re-written as follows

(21) $$x_{t}^{\prime }={\overset{˜}{z}}_{t}{\left(\pi^{0}\right)}^{\prime }\Theta_{0}+v_{t}^{\prime }\text{,}\;t=1,2,\ldots ,T$$

where $\Theta_{0}={\left[\Delta_{0}^{{\left(1\right)}^{\prime }},\Delta_{0}^{{\left(2\right)}^{\prime }},\ldots ,\Delta_{0}^{{\left(h+1\right)}^{\prime }}\right]}^{\prime },{\overset{˜}{z}}_{t}\left(\pi^{0}\right)=\iota \left(t,T\right)⊗z_{t},\iota \left(t,T\right)$ is a (h+1)×1 vector with first element $I\left\{t/T\in \left(0,\pi_{1}^{0}\right]\right\},h+1$th element $I\left\{t/T\in \left(\pi_{h}^{0},1\right]\right\},k$th element $I\left\{t/T\in \left(\pi_{k-1}^{0},\pi_{k}^{0}\right]\right\}$ for k=1,2,…,h and I{⋅} is an indicator variable that takes the value one if the event in the curly brackets occurs. Notice that (21) fits the generic constant parameter form of (8), and this similarity facilitates the analysis of the limiting properties of the estimators below.

Within our analysis, it is assumed that the break points in the reduced form are estimated prior to estimation of the structural equation in (7). For our analysis to go through, the estimated break fractions in the reduced form must satisfy certain conditions that are detailed below. Once the instability of the reduced form is incorporated into ${\overset{ˆ}{x}}_{t}$, the 2SLS estimation is implemented in the fashion described in Section 3. However, the presence of this additional source of instability means that it is also necessary to modify Assumption 7.

Assumption 18 The minimization in (12) is over all partitions $\left(T_{1},\ldots ,T_{m}\right)$ such that $T_{i}-T_{i-1}>\max\left\{q-1,\epsilon T\right\}$ for some ϵ>0 and $\epsilon <\inf_{i}\left(\lambda_{i+1}^{0}-\lambda_{i}^{0}\right)$ and $\epsilon <\inf_{j}\left(\pi_{j+1}^{0}-\pi_{j}^{0}\right)$.

The remainder of our discussion focuses on the unstable reduced form case. In part (i), we consider the limiting behaviour of the estimators of the break fraction and the structural parameters, and in part (ii) we consider hypothesis testing and estimation of the number of breaks.

(i) *Limiting behaviour of the estimators*.

We suppose that the vector of true break points in the reduced form, $\pi^{0}$, is estimated by $\overset{ˆ}{\pi }$, and these estimated breaks are imposed on the reduced form for $x_{t}$. Let ${\overset{ˆ}{\Theta }}_{T}$ be the OLS estimator of $\Theta_{0}$ from the model

(22) $$x_{t}^{\prime }={\overset{˜}{z}}_{t}{\left(\overset{ˆ}{\pi }\right)}^{\prime }\Theta_{0}+\text{error}\;t=1,2,\ldots ,T$$

where ${\overset{˜}{z}}_{t}\left(\overset{ˆ}{\pi }\right)$ is defined analogously to ${\overset{˜}{z}}_{t}\left(\pi^{0}\right)$, and now define ${\overset{ˆ}{x}}_{t}$to be

(23) $${\overset{ˆ}{x}}_{t}^{\prime }={\overset{˜}{z}}_{t}{\left(\overset{ˆ}{\pi }\right)}^{\prime }{\overset{ˆ}{\Theta }}_{T}={\overset{˜}{z}}_{t}{\left(\overset{ˆ}{\pi }\right)}^{\prime }{\left\{\overset{T}{\underset{t=1}{\sum }}{\overset{˜}{z}}_{t}\left(\overset{ˆ}{\pi }\right){\overset{˜}{z}}_{t}{\left(\overset{ˆ}{\pi }\right)}^{\prime }\right\}}^{-1}\overset{T}{\underset{t=1}{\sum }}{\overset{˜}{z}}_{t}\left(\overset{ˆ}{\pi }\right)x_{t}^{\prime }\text{.}$$

Below we present extensions of Theorems 1–3 to the unstable reduced form case. In our analysis we maintain Assumptions 8, 10 and 11, but need to also impose the following conditions.

Assumption 19 (i) $\overset{ˆ}{\pi }=\pi^{0}+O_{p}\left(T^{-1}\right)$; (ii) $rank\left\{\;\left[\Delta_{0}^{\left(i\right)},\;\Pi \right]\;\right\}=p$ for i=1,2,…,h+1 for Π defined in Assumption 9; (iii) there exists an $l_{*}$ with $0<l_{*}<\min\left\{T_{i}^{*},T-T_{i}^{*}\right\}$ such that for all $l>l_{*}$, with $l\leq \min\left\{T_{i}^{*},T-T_{i}^{*}\right\}$, the minimum eigenvalues of $B_{il}=\left(1/l\right)\sum_{t=T_{i}^{*}+1}^{T_{i}^{*}+l}z_{t}{z_{t}}^{\prime }$ and of $B_{il}^{*}=\left(1/l\right)\sum_{t=T_{i}^{*}-l}^{T_{i}^{*}}z_{t}{z_{t}}^{\prime }$ are bounded away from zero in probability, for all i=1,…,h+1.

Note that Assumption 19(i) implies $\overset{ˆ}{\pi }$ is consistent for $\pi^{0}$ and $T\left(\overset{ˆ}{\pi }-\pi^{0}\right)$ is bounded in probability. Such an estimator might be obtained by applying the methodology of Bai and Perron (1998), equation by equation, and then pooling the resulting estimates of the break fractions. For our purposes, it only matters that Assumption 19(i) holds and not how $\overset{ˆ}{\pi }$ is obtained. The latter is, of course, a matter of practical importance but its exploration is beyond the scope of this paper. Assumption 19(ii) plays an analogous role to Assumption 9. Assumption 19(iii) is similar to Assumption 10 above but refers to the reduced form.

The following theorem establishes the limiting properties of the 2SLS break point and coefficient estimators.

Theorem 8 *If* Assumptions 6*,* 8*,* 10*,* 11*,* 17–19(i)–(ii) *hold,* $y_{t}$ *is generated via* (7)*,* $x_{t}$ *is generated via* (21) *and* ${\overset{ˆ}{x}}_{t}$ *is calculated via* (23)*, then*

(i) ${\overset{ˆ}{\lambda }}_{j}\overset{p}{\to }\lambda_{j}^{0}\text{ for all }j=1,2,\ldots ,m$*.*
*If in addition,*Assumption 19(iii) *holds then:*
(ii) *For every* η>0*, there exists* C *such that for all large* $T,P\left(T\left|{\overset{ˆ}{\lambda }}_{j}-\lambda_{j}^{0}\right|>C\right)<\eta$*, for* j=1,…,m*.*
(iii) $T^{1/2}\left(\;\overset{ˆ}{\beta }\left({\left\{{\overset{ˆ}{T}}_{i}\right\}}_{i=1}^{m}\right)-\beta^{0}\;\right)\Rightarrow N\left(\;0_{p\left(m+1\right)\times 1},\;V_{\beta }\;\right)$ *where* $\beta^{0}={\left[{\beta_{1}^{0}}^{\prime },{\beta_{2}^{0}}^{\prime },\ldots ,{\beta_{h+1}^{0}}^{\prime }\right]}^{\prime },\beta_{i}^{0}={\left[{\beta_{x,i}^{0}}^{\prime },{\beta_{z_{1},i}^{0}}^{\prime }\right]}^{\prime }$*,*
  $$V_{\beta }=\left(\begin{array}{ccc} V_{\beta }^{\left(1,1\right)} & \cdots & V_{\beta }^{\left(1,m+1\right)}\\ \vdots & \ddots & \vdots \\ V_{\beta }^{\left(m+1,1\right)} & \cdots & V_{\beta }^{\left(m+1,m+1\right)} \end{array}\right)$$
  $$V_{i,i}={\overset{˜}{A}}_{i}\left\{{\overset{˜}{C}}_{i}{\overset{˜}{V}}_{i}{\overset{˜}{C}}_{i}^{\prime }-{\overset{˜}{E}}_{i}{\overset{˜}{D}}_{i}{\overset{˜}{V}}_{i}{\overset{˜}{C}}_{i}^{\prime }-{\overset{˜}{C}}_{i}{\overset{˜}{V}}_{i}{\overset{˜}{D}}_{i}^{\prime }{\overset{˜}{E}}_{i}^{\prime }+{\overset{˜}{E}}_{i}{\overset{˜}{D}}_{i}\overset{˜}{V}{\overset{˜}{D}}_{i}^{\prime }{\overset{˜}{E}}_{i}^{\prime }\right\}{\overset{˜}{A}}_{i}^{\prime }$$
  $$V_{i,j}={\overset{˜}{A}}_{i}{\overset{˜}{E}}_{i}{\overset{˜}{D}}_{i}\overset{˜}{V}{\overset{˜}{D}}_{j}^{\prime }{\overset{˜}{E}}_{j}^{\prime }{\overset{˜}{A}}_{j}^{\prime }-{\overset{˜}{A}}_{i}{\overset{˜}{E}}_{i}{\overset{˜}{D}}_{i}{\overset{˜}{V}}_{j}{\overset{˜}{C}}_{j}^{\prime }{\overset{˜}{A}}_{j}^{\prime }-\;{\overset{˜}{A}}_{i}{\overset{˜}{C}}_{i}{\overset{˜}{V}}_{i}{\overset{˜}{D}}_{j}^{\prime }{\overset{˜}{E}}_{j}^{\prime }{\overset{˜}{A}}_{j}^{\prime }\text{,}\;\text{for }i\neq j$$
  $${\overset{˜}{A}}_{i}={\left[{\overset{˜}{\Upsilon }}_{0}^{\prime }{\overset{˜}{Q}}_{i}{\overset{˜}{\Upsilon }}_{0}\right]}^{-1}{\overset{˜}{\Upsilon }}_{0}^{\prime }\text{,}\;{\overset{˜}{E}}_{i}={\overset{˜}{Q}}_{i}{\overset{˜}{Q}}_{ZZ}{\left(1\right)}^{-1}\text{,}\;\text{for }i=1,2,\ldots ,m+1$$
  $${\overset{˜}{\Upsilon }}_{0}^{\prime }=\left[\Upsilon_{1}^{\prime },\Upsilon_{2}^{\prime },\ldots ,\Upsilon_{h+1}^{\prime }\right]\text{,}\;\Upsilon_{i}=\left[\Delta_{0}^{\left(i\right)},\Pi \right]\text{,}$$
  $${\overset{˜}{C}}_{i}=\left[I_{\overset{˜}{q}},{\beta_{x,i}^{0}}^{\prime }⊗I_{\overset{˜}{q}}\right]\text{,}\;D_{i}=\left[0_{\overset{˜}{q}\times \overset{˜}{q}},{\beta_{x,i}^{0}}^{\prime }⊗I_{\overset{˜}{q}}\right]\text{,}\;\overset{˜}{q}=q\left(h+1\right)\text{,}$$
  $${\overset{˜}{Q}}_{i}={\overset{˜}{Q}}_{ZZ}\left(\lambda_{i}^{0}\right)-{\overset{˜}{Q}}_{ZZ}\left(\lambda_{i-1}^{0}\right)\text{,}\;{\overset{˜}{Q}}_{ZZ}\left(\lambda \right)=\mathrm{plim}\;T^{-1}\overset{\left[\lambda T\right]}{\underset{t=1}{\sum }}{\overset{˜}{z}}_{t}\left(\pi^{0}\right){\overset{˜}{z}}_{t}{\left(\pi^{0}\right)}^{\prime }$$
  $${\overset{˜}{V}}_{i}=\mathrm{V ar}\left[T^{-1/2}\overset{\left[\lambda_{i}T\right]}{\underset{t=\left[\lambda_{i-1}T\right]+1}{\sum }}{\overset{˜}{h}}_{t}\right]\text{,}\;\overset{˜}{V}=\mathrm{V ar}\left[T^{-1/2}\overset{T}{\underset{t=1}{\sum }}{\overset{˜}{h}}_{t}\right]\text{,}\;{\overset{˜}{h}}_{t}=\left(u_{t},v_{t}^{\prime }\right)⊗{\overset{˜}{z}}_{t}\left(\pi^{0}\right)\text{.}$$

Theorem 8(i)–(ii) show that the estimated break point exhibits similar limiting behaviour in the stable and unstable reduced form cases. Theorem 8(iii) reveals that, in general, the form of the covariance matrix depends on the relative locations of the breaks in the structural equation and the reduced form. However, it is worth noting that certain simplifications are possible in cases that may be of empirical relevance. First, if all the breaks in the structural and reduced form equations coincide then we have the following result.

Corollary 3 *Under the conditions of* Theorem 8(iii)*, if* m=h *and* $\lambda_{i}^{0}=\pi_{i}^{0}$ *for all* i=1,2,…,m*then* $V_{\beta }=\mathrm{diag}\left[V_{1,1},V_{2,2},\ldots ,V_{m+1,m+1}\right]$ *where* $V_{i,i}={\overset{¯}{A}}_{i}{\overset{¯}{H}}_{i}{\overset{¯}{A}}_{i}^{\prime }$ *where* ${\overset{¯}{A}}_{i}={\left[{\Upsilon_{i}}^{\prime }Q_{i}\Upsilon_{i}\right]}^{-1}{\Upsilon_{i}}^{\prime }$ *and* ${\overset{¯}{H}}_{i}=\lim_{T\to \infty }\mathrm{V ar}\left[T^{-1/2}\sum_{t=\left[\lambda_{i-1}^{0}T\right]+1}^{\left[\lambda_{i}^{0}T\right]}z_{t}u_{t}\right]$*.*

The intuition behind this result is that in this case the terms involving the reduced form error cancel out asymptotically in $T^{-1/2}\sum_{t=\left[\lambda_{i-1}^{0}T\right]+1}^{\left[\lambda_{i}^{0}T\right]}z_{t}{\overset{˜}{u}}_{t}$. Second, if there are more breaks in the reduced form than in the structural equation but all the breaks in the structural equation coincide with a corresponding break in the reduced form then we have the following result.

Corollary 4 *Under the conditions of* Theorem 8(iii)*, if* m<h *and* $\lambda_{i}^{0}=\pi_{j\left(i\right)}^{0}$ *for all* i=1,2,…,m *and some* j(i)*then* $V_{\beta }=\mathrm{diag}\left[V_{1,1},V_{2,2},\ldots ,V_{m+1,m+1}\right]$ *where* $V_{i,i}$ *is defined in* Theorem 8(iii)*.*

The intuition behind this result is that the pattern of the breaks means that there is no correlation asymptotically between the 2SLS estimators in different regimes.

(ii) *Hypothesis testing and estimation of the number of breaks*.

In the case where the reduced form is stable, it is possible to develop statistics with the distributions tabulated in Bai and Perron (1998). Unfortunately, these statistics do not appear to extend directly to the unstable reduced form case. For while the unstable reduced form in (20) can be re-written as a “stable reduced form” involving augmented parameter and instrument vectors, it does not satisfy the assumptions imposed in the derivation of the tests in Section 4. To illustrate this issue, consider the assumed behaviour of the instrument cross-product matrix, $T^{-1}\sum_{t=1}^{\left[Tr\right]}z_{t}z_{t}^{\prime }$. Under Assumption 12, the limit of this matrix is $rQ_{ZZ}$ and is thus linear in r. However, if we consider the augmented instrument cross-product matrix $T^{-1}\sum_{t=1}^{\left[Tr\right]}{\overset{˜}{z}}_{t}\left(\pi^{0}\right){\overset{˜}{z}}_{t}{\left(\pi^{0}\right)}^{\prime }$ then the limit of this matrix cannot be linear in r. In fact, if Assumption 12 holds and $\pi_{i-1}^{0}<r<\pi_{i}^{0}$ for some i then

$$T^{-1}\overset{\left[Tr\right]}{\underset{t=1}{\sum }}{\overset{˜}{z}}_{t}\left(\pi^{0}\right){\overset{˜}{z}}_{t}{\left(\pi^{0}\right)}^{\prime }\overset{p}{\to }\left(\pi_{1}^{0},\pi_{2}^{0}-\pi_{1}^{0},\ldots ,\pi_{i-1}^{0}-\pi_{i-2}^{0},r-\pi_{i-1}^{0},0_{1\times \left(h+1-i\right)}\right)⊗Q_{ZZ}\neq rM\text{,}\;\text{for some matrix }M\text{.}$$

A similar problem arises with the long run variance matrix $\lim_{T\to \infty }\mathrm{V ar}\left[T^{-1/2}\sum_{t=1}^{\left[Tr\right]}{\overset{˜}{h}}_{t}\right]$.^17^

However, it is possible to develop fixed break point tests within this setting and in this sub-section we show that such tests can be combined with those derived for the stable reduced form case to produce a method for estimation of m. This method turns out to be quite simple and thus has an appeal for practitioners. We first outline the method for estimation of m and then present the necessary fixed break point test statistic.

*Methodology for estimation of* m

1. Estimate reduced form and test for multiple changes in parameters using, for example, the methods in Bai and Perron (1998).
2.(a) If the reduced form is judged stable then use the methodology described in Section 4(iii) to estimate m.
2.(b) If the reduced form is unstable then estimate h using, for example, the methods in Bai and Perron (1998). Let $\overset{ˆ}{h}$ be the number of breaks, and collect the estimates into the $\overset{ˆ}{h}\times 1$ vector $\overset{ˆ}{\pi }$.
  (i) Divide the sample into $\overset{ˆ}{h}+1$ sub-samples: $T_{j}=\left\{t\in \left[{\overset{ˆ}{\tau }}_{j-1}+1,\ldots ,{\overset{ˆ}{\tau }}_{j}\right]\right\}$, where ${\overset{ˆ}{\tau }}_{j}=\left[{\overset{ˆ}{\pi }}_{j}T\right],{\overset{ˆ}{\pi }}_{0}=0$ and ${\overset{ˆ}{\pi }}_{h+1}=1$.
  (ii) Apply the methodology described in Section 4(iii) to estimate the number of breaks in the structural equation for $T_{j}$.^18^ Let $\overset{ˆ}{m}\left(j\right)$ be the number of breaks on this segment and denote the location of these breaks by ${\overset{ˆ}{\lambda }}_{i}\left(j\right)$ for $i=1,2,\ldots ,\overset{ˆ}{m}\left(j\right)$.
  (iii) Define $L=\left\{{\overset{ˆ}{\lambda }}_{i}\left(j\right);\;i=1,2,\ldots ,\overset{ˆ}{m}\left(j\right);\;j=1,2,\ldots ,\overset{ˆ}{h}\right\}$. Conditional on breaks in L, test whether there is a break in the structural equation at ${\overset{ˆ}{\tau }}_{j}$ for $j=1,2\ldots ,\overset{ˆ}{h}$ individually using the test statistic ${\mathrm{Wald}}_{T}\left(j\right)$ defined below. Define $L_{\pi }=\left\{{\overset{ˆ}{\pi }}_{j},\text{ for which }{\mathrm{Wald}}_{T}\left(j\right)\;\text{ is significant};j=1,2,\ldots ,\overset{ˆ}{h}\right\}$.^19^
  (iv) Estimated set of break points is $L\cup L_{\pi }$, and the estimated number of break points, $\overset{ˆ}{m}$, is the cardinality of $L\cup L_{\pi }$.

We now present the formula for ${\mathrm{Wald}}_{T}\left(j\right)$ and its limiting distribution. Suppose we wish to test the null hypotheses that there is a break in the structural equation at ${\overset{ˆ}{\tau }}_{j}$ conditional on the breaks in L. In this case, we can confine attention to the sample $t=\left[{\overset{ˆ}{\lambda }}_{\overset{ˆ}{m}\left(j-1\right)}\left(j-1\right)T\right]+1,\ldots ,\left[{\overset{ˆ}{\lambda }}_{1}\left(j\right)T\right]$ and employ the Wald test for a single (fixed) break at ${\overset{ˆ}{\tau }}_{j}$. To facilitate the exposition, we write the structural equation as:

$$y_{t}=\left(x_{t}^{\prime },z_{1,t}^{\prime }\right)b_{1}\left(j\right)+u_{t}\text{,}\;\text{for }t=\left[{\overset{ˆ}{\lambda }}_{\overset{ˆ}{m}\left(j-1\right)}\left(j-1\right)T\right]+1,\ldots ,{\overset{ˆ}{\tau }}_{j}=\left(x_{t}^{\prime },z_{1,t}^{\prime }\right)b_{2}\left(j\right)+u_{t}\text{,}\;\text{for }t={\overset{ˆ}{\tau }}_{j}+1,\ldots ,\left[{\overset{ˆ}{\lambda }}_{1}\left(j\right)T\right]\text{.}$$

Let $\left\{{\overset{ˆ}{b}}_{1}\left(j\right),\;{\overset{ˆ}{b}}_{2}\left(j\right)\right\}$ be the 2SLS estimators of $\left\{b_{1}\left(j\right),\;b_{2}\left(j\right)\right\}$; then, the appropriate Wald statistic is

(24) $${\mathrm{Wald}}_{T}\left(j\right)=T{\left\{{\overset{ˆ}{b}}_{1}\left(j\right)-\;{\overset{ˆ}{b}}_{2}\left(j\right)\right\}}^{\prime }{\left\{\overset{¯}{V}\left(j\right)\right\}}^{-1}\left\{{\overset{ˆ}{b}}_{1}\left(j\right)-\;{\overset{ˆ}{b}}_{2}\left(j\right)\right\}$$

where

$$\overset{¯}{V}\left(j\right)={\overset{¯}{V}}_{1}\left(j\right)+{\overset{¯}{V}}_{2}\left(j\right)\text{,}\;{\overset{¯}{V}}_{k}\left(j\right)={\overset{¯}{A}}_{k}\left\{{\overset{¯}{C}}_{k}V_{k}{\overset{¯}{C}}_{k}^{\prime }+{\overset{¯}{D}}_{k}V_{k}{\overset{¯}{D}}_{k}^{\prime }-c_{k}\left({\overset{¯}{C}}_{k}V_{k}{\overset{¯}{D}}_{k}^{\prime }+{\overset{¯}{D}}_{k}V_{k}{\overset{¯}{C}}_{k}^{\prime }\right)\right\}{\overset{¯}{A}}_{k}^{\prime }\text{,}$$

$${\overset{¯}{A}}_{1}={\left(\Upsilon_{j}^{\prime }{\overset{¯}{Q}}_{ZZ}^{\left(1\right)}\Upsilon_{j}\right)}^{-1}\Upsilon_{j}^{\prime }\text{,}\;{\overset{¯}{C}}_{1}={\left(\pi_{j}^{0}-\nu_{0}\right)}^{-1/2}\left[I_{q},{b_{x}\left(j\right)}^{\prime }⊗I_{q}\right]\text{,}\;\nu_{0}=\lambda_{l}\left(j\right)=\mathrm{plim}\;{\overset{ˆ}{\lambda }}_{\overset{ˆ}{m}\left(j-1\right)}\left(j-1\right)\text{,}$$

$${\overset{¯}{D}}_{1}={\left(\pi_{j}^{0}-\pi_{j-1}^{0}\right)}^{-1/2}\left[0_{q\times q},{b_{x}\left(j\right)}^{\prime }⊗I_{q}\right]\text{,}\;c_{1}={\left(\pi_{j}^{0}-\nu_{0}\right)}^{1/2}{\left(\pi_{j}^{0}-\pi_{j-1}^{0}\right)}^{-1/2}\text{,}$$

$${\overset{¯}{V}}_{1}=\underset{T\to \infty }{\lim}\mathrm{V ar}\left[\overset{-1/2}{\underset{1}{T}}\underset{1}{\sum }h_{t}\right]\text{,}\;T_{1}=\left(\overset{0}{\underset{j}{\pi }}-\nu_{0}\right)T\text{,}\;{\overset{¯}{Q}}_{ZZ}^{\left(1\right)}=\mathrm{plim}\;T_{1}^{-1}\underset{1}{\sum }z_{t}\overset{\prime }{\underset{t}{z}}\text{,}$$

$${\overset{¯}{A}}_{2}={\left(\Upsilon_{j+1}^{\prime }{\overset{¯}{Q}}_{ZZ}^{\left(2\right)}\Upsilon_{j+1}\right)}^{-1}\Upsilon_{j+1}^{\prime }\text{,}\;{\overset{¯}{C}}_{2}={\left(\nu_{1}-\pi_{j}^{0}\right)}^{-1/2}\left[I_{q},{b_{x}\left(j\right)}^{\prime }⊗I_{q}\right]\text{,}\;\nu_{1}=\lambda_{u}\left(j\right)=\mathrm{plim}\;{\overset{ˆ}{\lambda }}_{1}\left(j\right)\text{,}$$

$${\overset{¯}{D}}_{2}={\left(\pi_{j+1}^{0}-\pi_{j}^{0}\right)}^{-1/2}\left[0_{q\times q},{b_{x}\left(j\right)}^{\prime }⊗I_{q}\right]\text{,}\;c_{2}={\left(\nu_{1}-\pi_{j}^{0}\right)}^{1/2}{\left(\pi_{j+1}^{0}-\pi_{j}^{0}\right)}^{-1/2}\text{,}$$

$${\overset{¯}{V}}_{2}=\underset{T\to \infty }{\lim}\mathrm{V ar}\left[\overset{-1/2}{\underset{2}{T}}\underset{2}{\sum }h_{t}\right]\text{,}\;T_{2}=\left(\nu_{1}-\overset{0}{\underset{j}{\pi }}\right)T\text{,}\;{\overset{¯}{Q}}_{ZZ}^{\left(2\right)}=\mathrm{plim}\;T_{2}^{-1}\underset{2}{\sum }z_{t}\overset{\prime }{\underset{t}{z}}\text{,}$$

$\sum_{1}$ denotes summation over $t=\left[\nu_{0}T\right]+1,\ldots ,\left[\pi_{j}^{0}T\right]$, $\sum_{2}$ denotes summation over $t=\left[\pi_{j}^{0}T\right]+1,\ldots ,\left[\nu_{1}T\right]$ and $b\left(j\right)={\left[b_{x}{\left(j\right)}^{\prime },b_{z_{1}}{\left(j\right)}^{\prime }\right]}^{\prime }$ is the common value of $\left\{\beta_{i}\left(j\right),\;i=1,2\right\}$ under $H_{0}$.

Theorem 9 *If* Assumptions 6, 8, 10, 11, 14 and 17–19 *hold,* $y_{t}$ *is generated via* (7)*,* $x_{t}$ *is generated via* (21) *and* ${\overset{ˆ}{x}}_{t}$ *is calculated via* (23) *then under* $H_{0}:b_{1}\left(j\right)=b_{2}\left(j\right)$*, we have* ${\mathrm{Wald}}_{T}\left(j\right)\overset{d}{\to }\chi_{p}^{2}$*.*

There may be strong reasons to suppose that a break in the reduced form is either present in the structural equation or it is not, and thus the outcome of the Wald test is sufficient to distinguish between these two states of the world. However, since the Wald test has power against other break points, it may be advisable to re-estimate the structural equation on $t=\left[{\overset{ˆ}{\lambda }}_{m\left(j-1\right)}\left(j-1\right)T\right]+1,\ldots ,\left[{\overset{ˆ}{\lambda }}_{1}\left(j\right)T\right]$ to determine the location of the break.

(iii) *Finite sample performance*.

We now investigate the finite sample properties of the Wald statistic and the methodology for estimation of m discussed above. Data are generated from the structural equation,

$$y_{t}=\left[1,x_{t}\right]\beta^{\left(i\right)}+u_{t}$$

where i=1 if $t/T\leq \lambda^{0}$, and i=2 else, and the reduced form

$$x_{t}=z_{t}^{\prime }\delta^{\left(j\right)}+v_{t}$$

where j=1 if $t/T\leq \pi^{0}$, and j=2 else. The vector $z_{t}$ is 5×1 and includes the intercept with the other elements being independent draws from a 4×1 standard normal distribution. The reduced form parameters are $\delta^{\left(i\right)}={\left(-1\right)}^{i+1}\left[1,d\right]$, for i=1,2, and d is chosen to ensure the population $R^{2}=0.5$; see footnote 5. We consider three scenarios of interest: *Case* I, no breaks in the structural but a break in the reduced form, ($\lambda^{0}=0$), $\beta^{\left(i\right)}={\left[1,0.1\right]}^{\prime },i=1,2;\pi^{0}=0.5$; *Case* II, a coincident break in the structural equation and the reduced form, $\lambda^{0}=\pi^{0}=0.5,\beta^{\left(i\right)}={\left(-1\right)}^{i+1}{\left[1,0.1\right]}^{\prime }$; *Case* III, a break in both equations but at distinct points in the sample, $\lambda^{0}=0.6,\pi^{0}=0.4,\beta^{\left(i\right)}={\left(-1\right)}^{i+1}{\left[1,0.1\right]}^{\prime }$. All other aspects of the data generation process for the reduced form are the same as in the stable reduced form case. In estimation of the reduced form, the number of breaks is assumed known to be one but its location is unknown and so estimated. A maximum of three breaks in the structural equation is allowed in each sub-sample.

The results are presented in Table 9. We report results using both 5% and 1% significance levels for all tests. We find that if a 5% significance level is used then the true number of breaks in the structural equation is estimated at least 84% of the time; if a 1% level is used then the minimum is at least 96% of the time. In no cases is the number of breaks estimated to be too small but there is a chance of overfitting. The latter is to be expected given the basis in hypothesis testing. Our results clearly indicate that a 1% significance level appears preferable because it leads to a very small probability of overfitting. In Case III where the breaks do not coincide, the methodology yields reliable estimators of the location of the break in the structural equation with 97.9% of the replications yielding an estimator within 0.03 of the true break fraction at T=240 and 99.5% at T=480. Overall, our methodology appears to work well within this design when implemented with 1% significance level tests. Further work is needed to explore the properties of the methodology in other settings. Nevertheless, these initial results are encouraging.

## 6 Empirical application

In this section, we use our methods to explore the stability of the New Keynesian Phillips Curve (NKPC) model for US data. Zhang et al. (2008) report that the stylized version of the NKPC does not have serially uncorrelated errors, so we follow their practice and include lagged values of the change in inflation $\Delta \inf_{t}=\inf_{t}-\inf_{t-1}$ to remove this dynamic structure from the errors.^20^ Accordingly, our analysis is based on the following NKPC version:

(25) $$\underset{t}{\inf}=c_{0}+\alpha_{f}\overset{e}{\underset{t+1|t}{\inf}}+\alpha_{b}\underset{t-1}{\inf}+\alpha_{\mathrm{og}}{\mathrm{og}}_{t}+\overset{3}{\underset{i=1}{\sum }}\alpha_{i}\Delta \underset{t-i}{\inf}+u_{t}\text{.}$$

Whether in Eq. (25) the usual output gap measure or a real marginal cost measure should be used to study the trade-off between inflation and unemployment over the cycle is an issue at the centre of a current debate.^21^Gali and Gertler (1999) attribute the usual findings of negative $\alpha_{\mathrm{og}}$ to measurement error in potential output, and argue that real marginal cost better accounts for direct productivity gains on inflation. On the other hand, real marginal cost is also unobserved, and other authors, e.g. Rudd and Whelan (2005) argue that the current practice of replacing marginal cost with average unit labour cost has little theoretical foundations. In our framework, we find–for the sub-samples with enough observations–evidence of a trade-off between inflation and unemployment (to the extent that output gap reflects employment), and a measure that would more directly reflect productivity gains on inflation would only be expected to strengthen our result.

We use quarterly US data spanning 1968.3–2001.4. The span of the data is slightly longer than Zhang et al. (2008) but the definitions of the variables are the same: $\inf_{t}$ is the annualized quarterly growth rate of the GDP deflator, ${\mathrm{og}}_{t}$ is obtained from the estimates of potential GDP published by the Congressional Budget Office, $\inf_{t+1|t}^{e}$ is the Greenbook one quarter ahead forecast of inflation prepared within the Fed.^22^

Both expected inflation and output gap are endogenous, with reduced forms:

(26) $$\overset{e}{\underset{t+1|t}{\inf}}=z_{t}^{\prime }\delta_{1}+v_{1,t}$$

(27) $${\mathrm{og}}_{t}=z_{t}^{\prime }\delta_{2}+v_{2,t}$$

where $z_{t}$ contains all other explanatory variables on the right-hand side of (25) along with the first lagged value of each of the short term interest rate, the unemployment rate, and the growth rate of the money aggregate M2.

Before applying our methodology, we first test for any evidence of weak identification. For our data, Stock and Yogo’s (2005) minimum eigenvalue statistic equals 15.22, which indicates we can reject the hypothesis of a maximum 5% bias ratio of 2SLS to OLS, and thus provides evidence that weak identification is not a problem.^23^ This corroborates the findings reported in Zhang et al. (2008).

We first assess the stability of the reduced forms in (26)–(27) via Bai and Perron’s (1998) methodology.^24^ We assume that the maximum number of breaks is 5 and set ϵ=0.1. The results are reported in Table 10. First consider the reduced form for $\inf_{t+1|t}^{e}$. There is clear evidence of parameter variation with all the sup-F statistics being significant at the 1% level. Using the sequential testing strategy, we identify two breaks: one at 1975.2 and the other at 1981.1. As a robustness check, we also use BIC to choose the break points and obtain the same estimates.^25^ Now consider the reduced form for ${\mathrm{og}}_{t}$. Again, there is evidence of parameter variation. The sequential strategy suggests a break at 1975.2. In contrast, BIC favours the model with no breaks. As pointed out in the sequential strategy of Section 5, for our purposes, it does not matter whether the break at 1975.2 occurs in both reduced forms or not; only the union of all breaks in the reduced forms counts.

This union is {1975.2,1981.1}, thus there are three sub-samples, each with stable reduced forms. According to the methodology described in Section 5, we test each of the sub-samples for additional unknown breaks in the structural equation, possibly present because of other structural parts of the economy not modelled here. The outcomes of sup-F tests and sup-Wald tests–robust to heteroscedasticity–all proposed in Section 4–are reported in Table 11. In this table, we define the BIC for a certain number of breaks m as

$$\mathrm{BIC}\left(m\right)=\ln\left[\underset{T_{1},\ldots ,T_{m}}{\min}S_{T}\left(T_{1},\ldots ,T_{m};\;\overset{ˆ}{\delta }\left(\overset{m}{\underset{i=1}{\left\{T_{i}\right\}}}\right)\right)/T\right]+m\left(p+1\right)\ln\left(T\right)/T\text{.}$$

The first two sub-samples are quite small, so we test for maximum one break in the first two sub-samples and maximum two breaks in the last. The results for all samples, coupled with BIC, suggest no further evidence of breaks. Next, we use fixed break-point tests to test whether the breaks in the reduced form coincide with those in the structural equation. The p-values for F tests and Wald tests are respectively: 0.001, 0.003 for a break at 1975.2 and 0.000, 0.000 for a break at 1981.1, indicating that the structural equation features both breaks.

The predicted values for NKPC for the period 1981.1–2001.4^26^ are as follows (standard errors in parentheses):

$$\underset{t}{\inf}=-\underset{\left(0.04\right)}{0.23}+\underset{\left(0.19\right)}{0.60}\overset{e}{\underset{t+1|t}{\inf}}+\underset{\left(0.18\right)}{0.22}\underset{t-1}{\inf}+\underset{\left(0.05\right)}{0.06}{\mathrm{og}}_{t}-\underset{\left(0.16\right)}{0.20}\Delta \underset{t-1}{\inf}-\underset{\left(0.14\right)}{0.20}\Delta \underset{t-2}{\inf}-\underset{\left(0.10\right)}{0.22}\Delta \underset{t-3}{\inf}\text{.}$$

Our results suggest that the forward-looking component of inflation dominates the backward-looking component, in accordance to Zhang et al. (2008). Our results also closely match Zhang et al.’s (2008) findings with regard to the location of first break, but we find evidence of a second break at 1981.1.^27^

## 7 Concluding remarks

In this paper, we propose a simple methodology for estimation and inference in linear regression models with endogenous regressors and multiple breaks. We first show that an approach based on minimizing a GMM criterion over all possible partitions does not yield, in general, consistent estimates of the break-fractions and parameters; in contrast, methods based on 2SLS do deliver consistent estimates due to a more promising construction of the minimand. The methods we propose are based on a sequential strategy in which the reduced form is first tested for breaks and if breaks are present then this information is incorporated into the estimation of the structural equation. We illustrate our methods via simulations and an empirical application to the NKPC for US. We show that the NKPC over the period of study is subject to instability, confirming findings such as in Zhang et al. (2008).

An interesting aspect of our analysis is that we show the limiting distribution of various tests for structural stability is not invariant to the nature of the reduced form. Specifically, if the reduced form is stable then we show that the tests based on our 2SLS estimators have the same limiting distribution derived by Bai and Perron (1998) for the analogous tests based on OLS estimators in a linear model with exogenous regressors. However, if the reduced form is unstable then the limiting distribution is different. This highlights the importance of assessing the structural stability of the reduced form prior to analysing the structural equation.

## Figures and Tables

**Table 1:** Empirical coverage of parameter confidence intervals.

q	T	One break model with stable reduced form						
		Confidence intervals						
			Intercept			Slope		
			99%	95%	90%	99%	95%	90%
4	120	1st regime	0.99	0.95	0.90	0.99	0.96	0.90
		2nd regime	0.99	0.94	0.87	0.98	0.94	0.88
	240	1st regime	0.99	0.94	0.90	0.99	0.95	0.90
		2nd regime	0.98	0.94	0.88	0.99	0.94	0.89
	480	1st regime	0.99	0.95	0.90	0.99	0.96	0.92
		2nd regime	0.98	0.94	0.89	0.99	0.94	0.87
8	120	1st regime	0.98	0.94	0.90	0.99	0.94	0.89
		2nd regime	0.99	0.95	0.89	0.99	0.95	0.89
	240	1st regime	0.99	0.95	0.91	0.99	0.95	0.91
		2nd regime	0.98	0.95	0.90	0.99	0.95	0.90
	480	1st regime	0.99	0.94	0.89	0.99	0.94	0.89
		2nd regime	0.98	0.95	0.91	0.99	0.95	0.90

*Notes*: The column headed 100a% gives the percentage of times the confidence intervals contain the corresponding true parameter values.

**Table 2:** Relative rejection frequencies of test statistics.

One break model with stable reduced form							
q	T	supF(k)		F(l+1|l)			F-UDmax
		1	2	2:1	3:2	4:3	
4	120	1.00	1.00	0.021	0.001	0.001	1.00
	240	1.00	1.00	0.028	0	0	1.00
	480	1.00	1.00	0.030	0	0	1.00
8	120	1.00	1.00	0.033	0.001	0	1.00
	240	1.00	1.00	0.028	0.003	0	1.00
	480	1.00	1.00	0.033	0.001	0	1.00

		supWald(k)		Wald(l+1|l)			W-UDmax
		1	2	2:1	3:2	4:3	

4	120	1.00	1.00	0.043	0.003	0	1.00
	240	1.00	1.00	0.035	0.001	0	1.00
	480	1.00	1.00	0.029	0.001	0	1.00
8	120	1.00	1.00	0.054	0.006	0.001	1.00
	240	1.00	1.00	0.039	0.001	0	1.00
	480	1.00	1.00	0.039	0.001	0	1.00

*Notes*: supF(k) denotes the statistic $\mathrm{Sup}\text{-}F_{T}\left(k;1\right);F\left(l+1|l\right)$ denotes the statistic $F_{T}\left(l+1|l\right)$ and the second tier column beneath it denotes l+1:l; F-UDmax denotes the statistic ${\mathrm{UDmaxF}}_{T}\left(5,1\right);\mathrm{supWald}\left(k\right)$ denotes the statistic $\mathrm{Sup}\text{-}{\mathrm{Wald}}_{T}\left(k;1\right)$; Wald(l+1|l) denotes the statistic ${\mathrm{Wald}}_{T}\left(l+1|l\right)$ and the second tier column beneath it denotes l+1:l; W-UDmax denotes the statistic ${\mathrm{UDmaxWald}}_{T}\left(5,1\right)$; the second tier column under the sup tests denotes either k or l+1:l as appropriate; q is the number of instruments; T is the sample size.

**Table 3:** Empirical distribution of the estimated number of breaks.

One break model with stable reduced form									
q	T	F-UDmax				W-UDmax			
		0	1	2	3	0	1	2	3
4	120	0	0.979	0.021	0	0	0.957	0.043	0
	240	0	0.972	0.028	0	0	0.965	0.035	0
	480	0	0.970	0.030	0	0	0.971	0.029	0
8	120	0	0.967	0.032	0.001	0	0.946	0.053	0.001
	240	0	0.972	0.027	0.001	0	0.961	0.039	0
	480	0	0.967	0.033	0	0	0.961	0.039	0

*Notes*: The figures in the block headed F-UDmax(W-UDmax) give the empirical distribution of the estimated number of breaks, ${\overset{ˆ}{m}}_{T}$, obtained via the sequential strategy using ${\mathrm{UDmaxF}}_{T}\left(5,1\right)\;\left({\mathrm{UDmaxWald}}_{T}\left(5,1\right)\right)$. In each case, L (the maximum number of breaks) is set equal to five and all tests are performed with a nominal 5% significance level; ${\overset{ˆ}{m}}_{T}>3$ in none of the simulations.

**Table 4:** Empirical coverage of parameter confidence intervals.

*Two break model with stable reduced form*								
q	T	Confidence intervals						
			Intercept			Slope		
			99%	95%	90%	99%	95%	90%
4	120	1st regime	0.99	0.93	0.89	0.99	0.94	0.89
		2nd regime	0.98	0.93	0.88	0.98	0.93	0.87
		3rd regime	0.98	0.92	0.86	0.98	0.95	0.89
	240	1st regime	0.99	0.95	0.90	0.99	0.94	0.90
		2nd regime	0.99	0.95	0.89	0.98	0.95	0.90
		3rd regime	0.99	0.95	0.90	0.99	0.95	0.89
	480	1st regime	0.99	0.96	0.89	0.99	0.95	0.91
		2nd regime	0.99	0.95	0.88	0.99	0.94	0.89
		3rd regime	0.99	0.95	0.91	1.00	0.96	0.91
8	120	1st regime	0.99	0.95	0.90	0.99	0.94	0.89
		2nd regime	0.98	0.94	0.89	0.98	0.94	0.88
		3rd regime	0.98	0.93	0.88	0.99	0.95	0.88
	240	1st regime	0.99	0.95	0.92	0.99	0.95	0.90
		2nd regime	0.99	0.94	0.89	0.99	0.94	0.88
		3rd regime	0.98	0.93	0.89	0.98	0.94	0.87
	480	1st regime	0.99	0.95	0.90	0.99	0.95	0.90
		2nd regime	0.99	0.94	0.89	0.99	0.94	0.89
		3rd regime	0.99	0.94	0.90	0.99	0.95	0.90

*Notes*: See Table 1 for definitions.

**Table 5:** Relative rejection frequencies of test statistics.

*Two break model with stable reduced form*						
q	T	supF(k)		F(l+1|l)		F-UDmax
		1	2	2:1	3:2	
4	120	1.00	1.00	1.00	0.021	1.00
	240	1.00	1.00	1.00	0.013	1.00
	480	1.00	1.00	1.00	0.015	1.00
8	120	1.00	1.00	1.00	0.015	1.00
	240	1.00	1.00	1.00	0.007	1.00
	480	1.00	1.00	1.00	0.010	1.00

		supWald(k)		Wald(l+1|l)		W-UDmax
		1	2	2:1	3:2	

4	120	1.00	1.00	1.00	0.033	1.00
	240	1.00	1.00	1.00	0.013	1.00
	480	1.00	1.00	1.00	0.012	1.00
8	120	1.00	1.00	1.00	0.028	1.00
	240	1.00	1.00	1.00	0.012	1.00
	480	1.00	1.00	1.00	0.013	1.00

*Notes*: See Table 2 for definitions.

**Table 6:** Empirical distribution of the estimated number of breaks.

*Two break model with stable reduced form*									
q	T	F-UDmax				W-UDmax			
		0	1	2	3	0	1	2	3
4	120	0	0	0.961	0.039	0	0	0.953	0.047
	240	0	0	0.984	0.016	0	0	0.982	0.018
	480	0	0	0.987	0.013	0	0	0.989	0.011
8	120	0	0	0.962	0.038	0	0	0.947	0.053
	240	0	0	0.978	0.022	0	0	0.976	0.024
	480	0	0	0.987	0.013	0	0	0.985	0.015

*Notes*: See Table 3 for definitions.

**Table 7:** Relative rejection frequencies of test statistics.

*No break model*									
q	T	supF(k)					F(l+1|l)		F-UDmax
		1	2	3	4	5	2:1	3:2	
4	120	0.051	0.058	0.050	0.051	0.045	0.013	0.001	0.053
	240	0.052	0.054	0.047	0.043	0.037	0.013	0.003	0.058
	480	0.060	0.059	0.058	0.068	0.057	0.008	0.001	0.060
8	120	0.043	0.042	0.053	0.049	0.045	0.014	0	0.045
	240	0.052	0.039	0.042	0.042	0.039	0.005	0	0.049
	480	0.058	0.057	0.058	0.052	0.050	0.017	0.001	0.062

		supWald(k)					Wald(l+1|l)		W-UDmax
		1	2	3	4	5	2:1	3:2	

4	120	0.074	0.093	0.083	0.077	0.075	0.018	0.007	0.93
	240	0.072	0.079	0.071	0.065	0.058	0.011	0.004	0.083
	480	0.060	0.063	0.064	0.071	0.063	0.01	0	0.064
8	120	0.064	0.083	0.090	0.085	0.077	0.024	0.007	0.089
	240	0.073	0.068	0.072	0.070	0.057	0.008	0.001	0.086
	480	0.066	0.075	0.070	0.065	0.062	0.014	0	0.075

*Notes*: See Table 2 for definitions.

**Table 8:** Empirical distribution of the estimated number of breaks.

*No break model*									
q	T	F-UDmax				W-UDmax			
		0	1	2	3	0	1	2	3
4	120	0.947	0.048	0.005	0	0.907	0.087	0.006	0
	240	0.942	0.053	0.005	0	0.917	0.079	0.003	0.001
	480	0.940	0.056	0.004	0	0.936	0.059	0.005	0
8	120	0.955	0.039	0.006	0	0.911	0.078	0.011	0
	240	0.951	0.046	0.003	0	0.914	0.084	0.002	0
	480	0.938	0.055	0.007	0	0.925	0.071	0.004	0

*Notes*: See Table 3 for definitions.

**Table 9:** Distribution of the estimated number of breaks with unstable reduced form.

Case	T	α	Wald	Relative frequency of $\overset{ˆ}{m}$			
				0	1	2	3
I	240	0.05	0.088	0.856	0.102	0.004	0
	240	0.01	0.021	0.963	0.031	0.006	0
	480	0.05	0.081	0.868	0.098	0.034	0
	480	0.01	0.013	0.977	0.021	0.002	0
II	240	0.05	1.000	0	0.892	0.104	0.004
	240	0.01	0.998	0	0.974	0.026	0
	480	0.05	1.000	0	0.917	0.082	0.001
	480	0.01	1.000	0	0.979	0.021	0
III	240	0.05	0.073	0	0.845	0.133	0.021
	240	0.01	0.020	0	0.963	0.033	0.004
	480	0.05	0.082	0	0.875	0.099	0.026
	480	0.01	0.010	0	0.980	0.018	0.002

*Notes*: Case I: no breaks in the structural equation, one in the reduced form; Case II: a coincident break in the structural equation and the reduced form; Case III: distinct breaks in the structural equation and the reduced form. α denotes the nominal significance level of all tests. Wald denotes the rejection frequency of the Wald test in (24). $\overset{ˆ}{m}$ is the estimated number of breaks using the methodology in Section 5.

**Table 10:** Application to NKPC—stability statistics for the reduced forms.

Dep.var	k	sup-F	F(k+1|k)	BIC
$\inf_{t+1|t}^{e}$	0			−0.615
	1	43.6	41.7	−0.623
	2	67.0	10.4	−0.680
	3	176.5	34.3	−0.649
	4	80.5	46.8	−0.452
	5	70.2		−0.369
${\mathrm{og}}_{t}$	0			−0.663
	1	50.0	30.53	−0.552
	2	40.1	23.1	−0.497
	3	40.0	11.3	−0.276
	4	34.9	11.3	−0.046
	5	31.9		0.255

*Notes*: Dep. Var. denotes the dependent variable in the reduced form; sup-F is the test statistic for $H_{0}:\;m=0$ vs. $H_{1}:m=k;F\left(k+1|k\right)$ is the test statistic for $H_{0}:\;m=k$ vs. $H_{1}:m=k+1$. The percentiles for the statistics are for k=1,2,… respectively: (i) sup-F: (10%, 1%) significance level = (25.29, 32.8), (23.33, 28.24), (21.89, 25.63), (20.71, 23.83), (19.63, 22.32); (ii) F(k+1:k): (10%, 1%) significance level = (25.29, 32.8), (27.59, 34.81), (28.75, 36.32), (29.71, 36.65).

**Table 11:** Application to NKPC—stability statistics for the structural equation.

Period	sup-F		UD-F	sup-Wald		UD-Wald	BIC		
	0:1	1:2	0:2	0:1	1:2	0:2	m=0	m=1	m=2
1968.4–1975.2	4.15	–	–	23.94	–	–	0.12	3.52	–
1975.3–1981.1	0.98	–	–	0.69	–	–	0.17	0.73	–
1981.2–2001.4	9.86	34.60	20.39	16.68	18.40	31.54	−1.08	−0.84	−0.84

*Notes*: The sign “–” indicates that tests have not been performed due to not enough observations in sub-samples, (0:k) is the statistic for testing $H_{0}:m=0$ vs. $H_{1}:m=k;\left(k:k+1\right)$ is the statistic for testing $H_{0}:m=k$ vs. $H_{1}:m=k+1$; UD indicates UDmax tests. The percentiles for both F-type and Wald-type statistics are at (10%, 1%) significance level respectively: (i) (0:1)=(19.7,26.71); (ii) (1:2)=(21.79,28.36); (iii) UDmax(0:2)=(20.00,26.75).

## Box I

(18) $$F_{T}\left(\ell +1|\ell \right)=\underset{1\leq i\leq \ell +1}{\max}\left\{\frac{{\mathrm{SSR}}_{\ell }\left({\overset{ˆ}{T}}_{1},\ldots ,{\overset{ˆ}{T}}_{\ell }\right)-\underset{\tau \in \Lambda_{i,\eta }}{\inf}{\mathrm{SSR}}_{\ell +1}\left({\overset{ˆ}{T}}_{1},\ldots ,{\overset{ˆ}{T}}_{i-1},\tau ,{\overset{ˆ}{T}}_{i},\ldots ,{\overset{ˆ}{T}}_{\ell }\right)}{\overset{2}{\underset{i}{\overset{ˆ}{\sigma }}}}\right\}$$

where

$${\overset{ˆ}{\sigma }}_{i}^{2}=\overset{{\overset{ˆ}{T}}_{i}}{\underset{t={\overset{ˆ}{T}}_{i-1}+1}{\sum }}{\left(y_{t}-{\overset{ˆ}{x}}_{t}^{\prime }{\overset{ˆ}{\beta }}_{x,i}-z_{1,t}^{\prime }{\overset{ˆ}{\beta }}_{z_{1},i}\right)}^{2}/\left({\overset{ˆ}{T}}_{i}-{\overset{ˆ}{T}}_{i-1}-p\right)$$

$$\Lambda_{i,\eta }=\left\{\tau :{\overset{ˆ}{T}}_{i-1}+\left({\overset{ˆ}{T}}_{i}-{\overset{ˆ}{T}}_{i-1}\right)\eta \leq \tau \leq {\overset{ˆ}{T}}_{i}-\left({\overset{ˆ}{T}}_{i}-{\overset{ˆ}{T}}_{i-1}\right)\eta \right\}$$

and ${\overset{ˆ}{\beta }}_{i}^{\prime }=\left({\overset{ˆ}{\beta }}_{x,i}^{\prime },{\overset{ˆ}{\beta }}_{z_{1},i}^{\prime }\right)$ is the 2SLS estimator calculated using the sample ${\overset{ˆ}{T}}_{i-1}+1,\ldots ,{\overset{ˆ}{T}}_{i}$ on the second stage.

## Appendix Mathematical appendix

### A.1 Results involving GMM

Proof of Proposition 1 Since $W_{T}\left(\lambda \right)$ is deterministic, we replace it by its limit in the proof without loss of generality, and for ease of notation we suppress its dependence on λ. Given the form of W, for $u_{t}\left(\theta \right)=y_{t}-x_{t}^{\prime }\theta$ and $f_{t,i}\left(\lambda \right)=u_{t}\left(\theta_{i}\left(\lambda \right)\right)z_{t},\;\left(i=1,2\right)$, we have

(28) $${\overset{˜}{Q}}_{T}\left(\theta \left(\lambda \right);\lambda \right)=E\left[\;T^{-2}\overset{\left[\lambda T\right]}{\underset{t,s=1}{\sum }}f_{t,1}{\left(\lambda \right)}^{\prime }\;W_{1}\;f_{t,1}\left(\lambda \right)\;\right]+E\left[\;T^{-2}\overset{T}{\underset{t,s=\left[\lambda T\right]+1}{\sum }}f_{t,2}{\left(\lambda \right)}^{\prime }\;W_{2}\;f_{t,2}\left(\lambda \right)\;\right]\equiv A_{1,T}\left(\lambda \right)+A_{2,T}\left(\lambda \right)\text{.}$$

*Case* 1: $\lambda =\lambda^{0}$. Set $T_{1}=\left[\lambda^{0}T\right]$ and $T_{2}=T-T_{1}$. Letting $E_{i}\left[\cdot \right],i=1,2$ denote the expectations taken with respect to the distribution of each regime, and using similar arguments to Han and Phillips (2006),

(29) $${\overset{˜}{Q}}_{T}\left(\theta \left(\lambda^{0}\right);\lambda^{0}\right)=\frac{T_{1}\left(T_{1}-1\right)}{T^{2}}\;E_{1}{\left[f_{t,1}\left(\lambda^{0}\right)\right]}^{\prime }\;W_{1}\;E_{1}\left[f_{t,1}\left(\lambda^{0}\right)\right]+\frac{1}{T^{2}}\overset{T_{1}}{\underset{t=1}{\sum }}\mathrm{tr}\left\{W_{1}\;E_{1}\left[f_{t,1}\left(\lambda^{0}\right)f_{t,1}{\left(\lambda^{0}\right)}^{\prime }\right]\right\}+\frac{T_{2}\left(T_{2}-1\right)}{T^{2}}\;E_{2}{\left[f_{t,2}\left(\lambda^{0}\right)\right]}^{\prime }\;W_{2}\;E_{2}\left[f_{t,2}\left(\lambda^{0}\right)\right]+\frac{1}{T^{2}}\overset{T}{\underset{t=T_{1}+1}{\sum }}\mathrm{tr}\left\{W_{2}\;E_{2}\left[f_{t,2}\left(\lambda^{0}\right)f_{t,2}{\left(\lambda^{0}\right)}^{\prime }\right]\right\}\text{.}$$

From (29) and Assumption 1, it follows that ${\overset{˜}{Q}}_{T}\left(\theta \left(\lambda^{0}\right);\lambda^{0}\right)\to \overset{˜}{Q}\left(\theta \left(\lambda^{0}\right)\right)$, with

(30) $$\overset{˜}{Q}\left(\theta \left(\lambda^{0}\right)\right)={\left(\lambda^{0}\right)}^{2}E_{1}{\left[f_{t,1}\left(\lambda^{0}\right)\right]}^{\prime }W_{1}E_{1}\left[f_{t,1}\left(\lambda^{0}\right)\right]+{\left(1-\lambda^{0}\right)}^{2}E_{2}{\left[f_{t,2}\left(\lambda^{0}\right)\right]}^{\prime }\;W_{2}\;E_{2}\left[f_{t,2}\left(\lambda^{0}\right)\right]\text{.}$$

Substituting $\theta \left(\lambda^{0}\right)=\theta_{0}\left(\lambda^{0}\right)$ in (30), it follows that $\overset{˜}{Q}\left(\theta \left(\lambda^{0}\right)\right)=0$. *Case* 2: $\lambda <\lambda^{0}$. Set $T_{1}=\left[\lambda T\right],T_{2}=\left[\lambda^{0}T\right]$, and $T_{*}=T_{2}-T_{1}$. Since $\lambda <\lambda^{0}$, we have

(31) $$A_{1,T}\left(\lambda \right)=\frac{T_{1}\left(T_{1}-1\right)}{T^{2}}\;E_{1}{\left[f_{t,1}\left(\lambda \right)\right]}^{\prime }W_{1}E_{1}\left[f_{t,1}\left(\lambda \right)\right]+\frac{1}{T^{2}}\overset{T_{1}}{\underset{t=1}{\sum }}\mathrm{tr}\left\{W_{1}\;E_{1}\left[f_{t,1}\left(\lambda \right)f_{t,1}{\left(\lambda \right)}^{\prime }\right]\right\}\text{.}$$

From (31) and Assumption 1, it follows that $A_{1,T}\left(\lambda \right)\to \lambda^{2}E_{1}{\left[f_{t,1}\left(\lambda \right)\right]}^{\prime },W_{1}\;E_{1}\left[f_{t,1}\left(\lambda \right)\right]$. Now consider $A_{2,T}\left(\lambda \right)$. We have

(32) $$A_{2,T}=E\left[\;T^{-2}\overset{T_{2}}{\underset{t=T_{1}}{\sum }}f_{t,2}{\left(\lambda \right)}^{\prime }\;W_{2}\;\overset{T_{2}}{\underset{t=T_{1}}{\sum }}f_{t,2}\left(\lambda \right)\;\right]+E\left[\;T^{-2}\overset{T}{\underset{t=T_{2}+1}{\sum }}f_{t,2}{\left(\lambda \right)}^{\prime }\;W_{2}\;\overset{T}{\underset{t=T_{2}+1}{\sum }}f_{t,2}\left(\lambda \right)\;\right]+2E\left[\;T^{-2}\overset{T_{2}}{\underset{t=T_{1}+1}{\sum }}f_{t,2}{\left(\lambda \right)}^{\prime }W_{2}\;\overset{T}{\underset{t=T_{2}+1}{\sum }}f_{t,2}\left(\lambda \right)\right]=a_{1,T}+a_{2,T}+2a_{3,T},\text{ respectively .}$$

Under our assumptions we have

(33) $$a_{1,T}\to {\left(\lambda^{0}-\lambda \right)}^{2}E_{1}\left[f_{t,2}{\left(\lambda \right)}^{\prime }\right]\;W_{2}\;E_{1}\left[f_{t,2}\left(\lambda \right)\right]\text{,}$$

(34) $$a_{2,T}\to \;{\left(1-\lambda^{0}\right)}^{2}E_{2}\left[f_{t,2}{\left(\lambda \right)}^{\prime }\right]\;W_{2}\;E_{2}\left[f_{t,2}\left(\lambda \right)\right]\text{,}$$

(35) $$a_{3,T}\to \left(\lambda^{0}-\lambda \right)\left(1-\lambda^{0}\right)E_{1}\left[f_{t,2}{\left(\lambda \right)}^{\prime }\right]\;W_{2}\;E_{2}\left[f_{t,2}\left(\lambda \right)\right]\text{.}$$

 Combining (31)–(35), yields ${\overset{˜}{Q}}_{T}\left(\theta \left(\lambda \right);\lambda \right)\to \overset{˜}{Q}\left(\theta \left(\lambda \right);\lambda \right)$, where

$$\overset{˜}{Q}\left(\theta \left(\lambda \right);\lambda \right)=\lambda^{2}E_{1}{\left[f_{t,1}\left(\lambda \right)\right]}^{\prime }\;W_{1}\;E_{1}\left[f_{t,1}\left(\lambda \right)\right]+{\left(\lambda^{0}-\lambda \right)}^{2}E_{1}\left[f_{t,2}{\left(\lambda \right)}^{\prime }\right]\;W_{2}\;E_{1}\left[f_{t,2}\left(\lambda \right)\right]+{\left(1-\lambda^{0}\right)}^{2}E_{2}\left[f_{t,2}{\left(\lambda \right)}^{\prime }\right]\;W_{2}\;E_{2}\left[f_{t,2}\left(\lambda \right)\right]+2\left(\lambda^{0}-\lambda \right)\left(1-\lambda^{0}\right)E_{1}\left[f_{t,2}{\left(\lambda \right)}^{\prime }\right]\;W_{2}\;E_{2}\left[f_{t,2}\left(\lambda \right)\right]\text{.}$$

We now evaluate the expectations above. Since $u_{t}\left(\theta \right)=u_{t}+x_{t}^{\prime }\left(\theta_{0}-\theta \right)$, it follows that $E_{i}\left[f_{t,j}\left(\lambda \right)\right]=M_{i}\left(\theta_{0}^{\left(i\right)}-\theta_{j}\left(\lambda \right)\right)$ and so,

(36) $$\overset{˜}{Q}\left(\theta \left(\lambda \right);\lambda \right)=\lambda^{2}{\left\{\theta_{0}^{\left(1\right)}-\theta_{1}\left(\lambda \right)\right\}}^{\prime }M_{1}^{\prime }W_{1}M_{1}\left\{\theta_{0}^{\left(1\right)}-\theta_{1}\left(\lambda \right)\right\}+{\left(\lambda^{0}-\lambda \right)}^{2}{\left\{\theta_{0}^{\left(1\right)}-\theta_{2}\left(\lambda \right)\right\}}^{\prime }D_{1}^{\prime }D_{1}\left\{\theta_{0}^{\left(1\right)}-\theta_{2}\left(\lambda \right)\right\}+{\left(1-\lambda^{0}\right)}^{2}{\left\{\theta_{0}^{\left(2\right)}-\theta_{2}\left(\lambda \right)\right\}}^{\prime }D_{2}^{\prime }D_{2}\left\{\theta_{0}^{\left(2\right)}-\theta_{2}\left(\lambda \right)\right\}+2\left(\lambda^{0}-\lambda \right)\left(1-\lambda^{0}\right){\left\{\theta_{0}^{\left(1\right)}-\theta_{2}\left(\lambda \right)\right\}}^{\prime }D_{1}^{\prime }D_{2}\left\{\theta_{0}^{\left(2\right)}-\theta_{2}\left(\lambda \right)\right\}$$

where $D_{i}=C_{2}M_{i}$, and $W_{i}=C_{i}^{\prime }C_{i}$ (where nonsingular $C_{i}$ exists via Assumption 5). Now notice that for $\theta \left(\lambda \right)={\left({\theta_{0}^{\left(1\right)}}^{\prime },{\theta_{*}^{\left(2\right)}}^{\prime }\right)}^{\prime }$, we have

$$\overset{˜}{Q}\left(\theta \left(\lambda \right);\lambda \right)=\left\{\frac{{\left(\lambda^{0}-\lambda \right)}^{2}{\left(1-\lambda^{0}\right)}^{2}}{{\left(1-\lambda \right)}^{2}}\right\}\xi^{\prime }\xi$$

where $\xi =C_{2}\left(M_{1}-M_{2}\right)\left(\theta_{0}^{\left(1\right)}-\theta_{0}^{\left(2\right)}\right)$. The result then follows immediately upon noting that $C_{2}$ is pd by definition. *Case* 3: $\lambda >\lambda^{0}$. This case can be handled similarly to Case 2 and is omitted for simplicity. □

Proof of Proposition 2 Define $Z_{1}\left(\lambda \right)={\left[z_{1},z_{2},\ldots ,z_{\left[\lambda T\right]}\right]}^{\prime }$, $Z_{2}\left(\lambda \right)={\left[z_{\left[\lambda T\right]+1},z_{\left[\lambda T\right]+2},\ldots ,z_{T}\right]}^{\prime },X_{1}\left(\lambda \right)={\left[x_{1},x_{2},\ldots ,x_{\left[\lambda T\right]}\right]}^{\prime },X_{2}\left(\lambda \right)={\left[x_{\left[\lambda T\right]+1},x_{\left[\lambda T\right]+2},\ldots ,x_{T}\right]}^{\prime },y_{1}\left(\lambda \right)={\left[y_{1},y_{2},\ldots ,y_{\left[\lambda T\right]}\right]}^{\prime },y_{2}\left(\lambda \right)={\left[y_{\left[\lambda T\right]+1},y_{\left[\lambda T\right]+2},\ldots ,y_{T}\right]}^{\prime }$. Since the model is linear, it follows by similar arguments to, for example, Hall (2005) [Chapter 2.2] that

(37) $$\left[\;\begin{array}{c} {\overset{ˆ}{\theta }}_{1,T}\left(\lambda \right)\\ {\overset{ˆ}{\theta }}_{2,T}\left(\lambda \right) \end{array}\;\right]=\left[\;\begin{array}{c} H_{1,T}Z_{1}{\left(\lambda \right)}^{\prime }y_{1}\left(\lambda \right)\\ H_{2,T}\left(\lambda \right)Z_{2}{\left(\lambda \right)}^{\prime }y_{2}\left(\lambda \right) \end{array}\;\right]$$

where $H_{i,T}\left(\lambda \right)={\left[X_{i}{\left(\lambda \right)}^{\prime }Z_{i}\left(\lambda \right)W_{i,T}\left(\lambda \right)Z_{i}{\left(\lambda \right)}^{\prime }X_{i}\left(\lambda \right)\right]}^{-1}X_{i}{\left(\lambda \right)}^{\prime }Z_{i}\left(\lambda \right)W_{i,T}\left(\lambda \right)$ for i=1,2. First consider ${\overset{ˆ}{\theta }}_{1,T}\left(\lambda \right)$. From Assumption 3, it follows that, uniformly in λ:

(38) $$T^{-1}X_{1}{\left(\lambda \right)}^{\prime }Z_{1}\left(\lambda \right)\overset{p}{\to }N_{1}\left(\lambda \right)\text{,}$$

(39) $$T^{-1}Z_{1}{\left(\lambda \right)}^{\prime }y_{1}\left(\lambda \right)\overset{p}{\to }\left\{\begin{array}{c} \lambda M_{1}\theta_{0}^{\left(1\right)}\text{,}\;\text{for }\lambda \leq \lambda^{0}\\ \lambda^{0}M_{1}\theta_{0}^{\left(1\right)}+\left(\lambda -\lambda^{0}\right)M_{2}\theta_{0}^{\left(2\right)}\text{,}\;\text{for }\lambda >\lambda^{0} \end{array}\right\}\text{,}$$

(40) $$T^{-1}X_{2}{\left(\lambda \right)}^{\prime }Z_{2}\left(\lambda \right)\overset{p}{\to }N_{2}\left(\lambda \right)\text{,}$$

(41) $$T^{-1}Z_{2}{\left(\lambda \right)}^{\prime }y_{2}\left(\lambda \right)\overset{p}{\to }\left\{\begin{array}{c} \left(\lambda^{0}-\lambda \right)M_{1}\theta_{0}^{\left(1\right)}+\left(1-\lambda^{0}\right)M_{2}\theta_{0}^{\left(2\right)}\text{,}\;\text{for }\lambda \leq \lambda^{0}\\ \left(1-\lambda \right)M_{2}\theta_{0}^{\left(2\right)}\text{,}\;\text{for }\lambda >\lambda^{0} \end{array}\right\}\text{.}$$

 Therefore, (37)–(41) yield ${\overset{ˆ}{\theta }}_{T}\left(\lambda \right)\overset{p}{\to }\overset{˜}{\theta }\left(\lambda \right)={\left[{\overset{˜}{\theta }}_{1}{\left(\lambda \right)}^{\prime },{\overset{˜}{\theta }}_{2}{\left(\lambda \right)}^{\prime }\right]}^{\prime }$ uniformly in λ where

(42) $${\overset{˜}{\theta }}_{1}\left(\lambda \right)=\theta_{0}^{\left(1\right)}I_{\lambda }\left(\lambda^{0}\right)+\left\{1-I_{\lambda }\left(\lambda^{0}\right)\right\}{\overset{¯}{\theta }}_{*}^{\left(1\right)}\left(\lambda \right)$$

(43) $${\overset{˜}{\theta }}_{2}\left(\lambda \right)={\overset{¯}{\theta }}_{*}^{\left(2\right)}\left(\lambda \right)I_{\lambda }\left(\lambda^{0}\right)+\left\{1-I_{\lambda }\left(\lambda^{0}\right)\right\}\theta_{0}^{\left(2\right)}$$

 where $I_{\lambda }\left(\lambda^{0}\right)$ is the indicator function defined in the statement of Proposition 3, and

(44) $${\overset{¯}{\theta }}_{*}^{\left(1\right)}\left(\lambda \right)={\left\{N_{1}{\left(\lambda \right)}^{\prime }W_{1}N_{1}\left(\lambda \right)\right\}}^{-1}N_{1}{\left(\lambda \right)}^{\prime }W_{1}\left[\lambda^{0}M_{1}\theta_{0}^{\left(1\right)}+\left(\lambda -\lambda^{0}\right)M_{2}\theta_{0}^{\left(2\right)}\right]$$

(45) $${\overset{¯}{\theta }}_{*}^{\left(2\right)}\left(\lambda \right)={\left\{N_{2}{\left(\lambda \right)}^{\prime }W_{2}N_{2}\left(\lambda \right)\right\}}^{-1}N_{2}{\left(\lambda \right)}^{\prime }W_{2}\left[\left(\lambda^{0}-\lambda \right)M_{1}\theta_{0}^{\left(1\right)}+\left(1-\lambda^{0}\right)M_{2}\theta_{0}^{\left(2\right)}\right]\text{.}$$

 From (44)–(45), it follows that if $\theta_{0}^{\left(1\right)}-\theta_{0}^{\left(2\right)}\in N\left(M_{1}-M_{2}\right)$ then ${\overset{¯}{\theta }}_{*}^{\left(i\right)}\left(\lambda \right)=\theta_{*}^{\left(i\right)}\left(\lambda \right)$ for i=1,2.^28^ □

To prove Proposition 3, we need the following lemma, whose proof is relegated to the Supplemental Appendix. Lemma A.1 *If* Assumptions 1–5 *hold and* $\theta_{0}^{\left(1\right)}-\theta_{0}^{\left(2\right)}\in N\left(M_{1}-M_{2}\right)$*, then:*

$$\left[\;\begin{array}{c} T^{1/2}\left({\overset{ˆ}{\theta }}_{1,T}\left(\lambda \right)-\theta_{*,1}\left(\lambda \right)\right)\\ T^{1/2}\left({\overset{ˆ}{\theta }}_{2,T}\left(\lambda \right)-\theta_{*,2}\left(\lambda \right)\right) \end{array}\;\right]\Rightarrow \left[\;\begin{array}{cc} H_{1}\left(\lambda \right) & 0_{p\times p}\\ 0_{p\times p} & H_{2}\left(\lambda \right) \end{array}\;\right]\;\left[\;\begin{array}{c} \xi_{1}\left(\lambda \right)\\ \xi_{2}\left(\lambda \right) \end{array}\;\right]$$

*where* $H_{i}\left(\lambda \right)$ *and* $\xi_{i}\left(\lambda \right)$ *are defined in* Proposition 3*.*

Proof of Proposition 3 Note that $J_{T}\left(\lambda \right)=TQ_{T}\left({\overset{ˆ}{\theta }}_{T}\left(\lambda \right);\lambda \right)=T^{1/2}g_{T}{\left({\overset{ˆ}{\theta }}_{T}\left(\lambda \right);\lambda \right)}^{\prime }W_{T}\left(\lambda \right)T^{1/2}g_{T}\left({\overset{ˆ}{\theta }}_{T}\left(\lambda \right);\lambda \right)$. Also,

(46) $$g_{T}\left({\overset{ˆ}{\theta }}_{T}\left(\lambda \right);\lambda \right)=\left[\;\begin{array}{c} T^{-1/2}\overset{\left[\lambda T\right]}{\underset{t=1}{\sum }}z_{t}\left(y_{t}-x_{t}^{\prime }{\overset{ˆ}{\theta }}_{1,T}\left(\lambda \right)\right)\\ T^{-1/2}\overset{T}{\underset{t=\left[\lambda T\right]+1}{\sum }}z_{t}\left(y_{t}-x_{t}^{\prime }{\overset{ˆ}{\theta }}_{2,T}\left(\lambda \right)\right) \end{array}\;\right]=\left[\;\begin{array}{c} c_{1,T}\left(\lambda \right)\\ c_{2,T}\left(\lambda \right) \end{array}\;\right]\text{.}$$

Noting that for i=1,2,

(47) $$y_{t}-x_{t}^{\prime }{\overset{ˆ}{\theta }}_{i,T}\left(\lambda \right)=u_{t}-x_{t}^{\prime }\left({\overset{ˆ}{\theta }}_{i,T}\left(\lambda \right)-\theta_{0}^{\left(1\right)}\right)\text{,}\;\text{for }t/T\leq \lambda^{0}$$

(48) $$=u_{t}-x_{t}^{\prime }\left({\overset{ˆ}{\theta }}_{i,T}\left(\lambda \right)-\theta_{0}^{\left(2\right)}\right)\text{,}\;\text{for }t/T>\lambda^{0}\text{,}$$

 it follows that for $\lambda \leq \lambda^{0}$,

(49) $$c_{1,T}\left(\lambda \right)=T^{-1/2}\overset{\left[\lambda T\right]}{\underset{t=1}{\sum }}z_{t}u_{t}-T^{-1}\overset{\left[\lambda T\right]}{\underset{t=1}{\sum }}z_{t}x_{t}^{\prime }T^{1/2}\left({\overset{ˆ}{\theta }}_{1,T}\left(\lambda \right)-\theta_{0}^{\left(1\right)}\right)\text{,}$$

and for $\lambda >\lambda^{0},c_{1,T}\left(\lambda \right)$ is given by^29^

(50) $$T^{-1/2}\overset{\left[\lambda T\right]}{\underset{t=1}{\sum }}z_{t}u_{t}-T^{-1}\overset{\left[\lambda^{0}T\right]}{\underset{t=1}{\sum }}z_{t}x_{t}^{\prime }T^{1/2}\left({\overset{ˆ}{\theta }}_{1,T}\left(\lambda \right)-\theta_{0}^{\left(1\right)}\right)-T^{-1}\overset{\left[\lambda T\right]}{\underset{t=\left[\lambda^{0}T\right]+1}{\sum }}z_{t}x_{t}^{\prime }T^{1/2}\left({\overset{ˆ}{\theta }}_{1,T}\left(\lambda \right)-\theta_{0}^{\left(2\right)}\right)=T^{-1/2}\overset{\left[\lambda T\right]}{\underset{t=1}{\sum }}z_{t}u_{t}-N_{1}\left(\lambda \right)T^{1/2}\left({\overset{ˆ}{\theta }}_{1,T}\left(\lambda \right)-\theta_{*}^{\left(1\right)}\left(\lambda \right)\right)-T^{-1/2}\overset{\left[\lambda^{0}T\right]}{\underset{t=1}{\sum }}\left(z_{t}x_{t}^{\prime }-M_{1}\right)\left[{\overset{ˆ}{\theta }}_{1,T}\left(\lambda \right)-\theta_{0}^{\left(1\right)}\right]-T^{-1/2}\overset{\left[\lambda T\right]}{\underset{t=\left[\lambda^{0}T\right]+1}{\sum }}\left(z_{t}x_{t}^{\prime }-M_{2}\right)\left[{\overset{ˆ}{\theta }}_{1,T}\left(\lambda \right)-\theta_{0}^{\left(2\right)}\right]\text{.}$$

Now consider $c_{2,T}\left(\lambda \right)$. Using (47)–(48) it follows that for $\lambda \leq \lambda^{0},c_{2,T}\left(\lambda \right)$ is given by^30^

(51) $$T^{-1/2}\overset{T}{\underset{t=\left[\lambda T\right]+1}{\sum }}z_{t}u_{t}-T^{-1}\overset{\left[\lambda^{0}T\right]}{\underset{t=\left[\lambda T\right]+1}{\sum }}z_{t}x_{t}^{\prime }T^{1/2}\left({\overset{ˆ}{\theta }}_{2,T}\left(\lambda \right)-\theta_{0}^{\left(1\right)}\right)-T^{-1}\overset{T}{\underset{t=\left[\lambda^{0}T\right]+1}{\sum }}z_{t}x_{t}^{\prime }T^{1/2}\left({\overset{ˆ}{\theta }}_{2,T}\left(\lambda \right)-\theta_{0}^{\left(2\right)}\right)=T^{-1/2}\overset{T}{\underset{t=\left[\lambda T\right]+1}{\sum }}z_{t}u_{t}-N_{2}\left(\lambda \right)T^{1/2}\left({\overset{ˆ}{\theta }}_{2,T}\left(\lambda \right)-\theta_{*}^{\left(1\right)}\right)-T^{-1/2}\overset{T}{\underset{t=\left[\lambda^{0}T\right]+1}{\sum }}\left(z_{t}x_{t}^{\prime }-M_{1}\right)\left[{\overset{ˆ}{\theta }}_{2,T}\left(\lambda \right)-\theta_{0}^{\left(2\right)}\right]-T^{-1/2}\overset{T}{\underset{t=\left[\lambda^{0}T\right]+1}{\sum }}\left(z_{t}x_{t}^{\prime }-M_{2}\right)\left[{\overset{ˆ}{\theta }}_{2,T}\left(\lambda \right)-\theta_{0}^{\left(2\right)}\right]$$

and for $\lambda >\lambda^{0}$

(52) $$c_{2,T}\left(\lambda \right)=T^{-1/2}\overset{T}{\underset{t=\left[\lambda T\right]+1}{\sum }}z_{t}u_{t}-T^{-1}\overset{T}{\underset{t=\left[\lambda T\right]+1}{\sum }}z_{t}x_{t}^{\prime }T^{1/2}\left({\overset{ˆ}{\theta }}_{2,T}\left(\lambda \right)-\theta_{0}^{\left(2\right)}\right)\text{.}$$

The result then follows from Eqs. (46)–(52), Proposition 2, Lemma A.1 and Assumptions 3–5. □

### A.2 Results involving 2SLS

We begin with an item of terminology. We say that a matrix A, say, is a diagonal partition at $\left(T_{1},T_{2},\ldots ,T_{m}\right)$ of the T×k matrix W whose tth row is ${{\overset{ˆ}{x}}_{t}}^{\prime }$ if $A=\mathrm{diag}\left(W_{T_{1}},\ldots ,W_{T_{m+1}}\right)$ and $W_{T_{i}}={\left({\overset{ˆ}{x}}_{T_{i-1}+1},\ldots ,{\overset{ˆ}{x}}_{T_{i}}\right)}^{\prime }$.^31^ Also, we write (10) for the true partition (so that $\beta_{i}^{*}=\beta_{i}^{0}$) as

(53) $$Y={\overset{¯}{W}}^{0}\beta^{0}+\overset{˜}{U}$$

where $Y={\left(y_{1},\ldots ,y_{T}\right)}^{\prime },{\overset{¯}{W}}^{0}$ is a diagonal partition of W at $\left(T_{1}^{0},\ldots ,T_{m+1}^{0}\right),\overset{˜}{U}={\left({\overset{˜}{u}}_{1},\ldots ,{\overset{˜}{u}}_{T}\right)}^{\prime }$, and $\beta^{0}=\beta^{0}\left({\left\{T_{i}^{0}\right\}}_{i=1}^{m}\right)={\left({\beta_{1}^{0}}^{\prime },{\beta_{2}^{0}}^{\prime },\ldots ,{\beta_{m+1}^{0}}^{\prime }\right)}^{\prime }$ with $\beta_{i}^{0}={\left(\beta_{i,1}^{0},\beta_{i,2}^{0},\ldots ,\beta_{i,p}^{0}\right)}^{\prime }$. We also define ${\overset{¯}{W}}^{*}$ to be a diagonal partition of W at $\left({\overset{ˆ}{T}}_{1},\ldots ,{\overset{ˆ}{T}}_{m}\right);Z={\left(z_{1},\ldots ,z_{T}\right)}^{\prime };V={\left(v_{1},\ldots ,v_{T}\right)}^{\prime }$.

We also need certain properties of matrix norms and we state these here for convenience. Corresponding to the vector (Euclidean) norm $\left\|x\right\|={\left(\sum_{i=1}^{p}x_{i}^{2}\right)}^{1/2}$ we define the matrix (Euclidean) norm as

(54) $$\left\|A\right\|=\underset{x\neq 0}{\sup}\left\|Ax\right\|/\left\|x\right\|$$

for matrix A. Below we use the following properties of this norm.

• ‖A‖ is equal to the square root of the maximum eigenvalue of $A^{\prime }A$ and thus,
  (55) $$\left\|A\right\|\leq {\left(\mathrm{tr}A^{\prime }A\right)}^{1/2}\text{.}$$
• For a projection matrix P, we have
  (56) ‖PA‖≤‖A‖.
• Let $A:R_{1}\to R_{2}$ and $B:R_{2}\to R_{3}$ be linear operators. Then we have^32^
  (57) ‖BA‖≤‖B‖‖A‖.

Finally, for a sequence of matrices, we write $A_{T}=o_{p}\left(1\right)$ if each of its element is $o_{p}\left(1\right)$, and likewise for $O_{p}\left(1\right)$.

*To simplify the presentation, we prove all the desired results for the special case in which* $\beta_{z_{1},i}^{0}=0_{p_{2}}$ *and* $z_{1,t}$ *is omitted from the structural equation during estimation. It is easily verified that all the desired results extend to the model presented in the main text*. Proof of Lemma 1 *Part* (i): Using the definition of $d_{t}$, it follows that, for $t\in \left[{\overset{ˆ}{T}}_{j-1}+1,{\overset{ˆ}{T}}_{j}\right],{\overset{˜}{u}}_{t}d_{t}={\overset{˜}{u}}_{t}{\overset{ˆ}{x}}_{t}^{\prime }\left({\overset{ˆ}{\beta }}_{j}-\beta_{i}^{0}\right)={\overset{˜}{u}}_{t}{\overset{ˆ}{x}}_{t}^{\prime }{\overset{ˆ}{\beta }}_{j}-{\overset{˜}{u}}_{t}{\overset{ˆ}{x}}_{t}^{\prime }\beta_{i}^{0}$ and hence that

(58) $$\overset{T}{\underset{t=1}{\sum }}{\overset{˜}{u}}_{t}d_{t}=\overset{T}{\underset{t=1}{\sum }}{\overset{˜}{u}}_{t}{\overset{ˆ}{x}}_{t}^{\prime }\overset{ˆ}{\beta }\left(t,T\right)-\overset{T}{\underset{t=1}{\sum }}{\overset{˜}{u}}_{t}{\overset{ˆ}{x}}_{t}^{\prime }\beta^{0}\left(t,T\right)={\overset{˜}{U}}^{\prime }{\overset{¯}{W}}^{*}\overset{ˆ}{\beta }-{\overset{˜}{U}}^{\prime }{\overset{¯}{W}}^{0}\beta^{0}$$

where $\overset{ˆ}{\beta }\left(t,T\right)=\sum_{i=1}^{m}{\overset{ˆ}{\beta }}_{j}\;I\left\{\;t/T\in \left({\overset{ˆ}{\lambda }}_{j-1},{\overset{ˆ}{\lambda }}_{j}\right]\;\right\}$ and $\beta^{0}\left(t,T\right)=\sum_{i=1}^{m}\beta_{j}^{0}\;I\left\{\;t/T\in \left(\lambda_{j-1},\lambda_{j}\right]\;\right\}$. From (58), it follows that Lemma 1(i) is established if it can be shown that

(59) $$T^{-1}\left({\overset{˜}{U}}^{\prime }{\overset{¯}{W}}^{*}\overset{ˆ}{\beta }-{\overset{˜}{U}}^{\prime }{\overset{¯}{W}}^{0}\beta^{0}\right)=o_{p}\left(1\right)\text{.}$$

Since the 2SLS estimator based on the partition $\left({\overset{ˆ}{T}}_{1},\ldots ,{\overset{ˆ}{T}}_{m}\right)$ is $\overset{ˆ}{\beta }={\left({\overset{¯}{W}}^{{*}^{\prime }}{\overset{¯}{W}}^{*}\right)}^{-1}{\overset{¯}{W}}^{{*}^{\prime }}Y$, it follows that

(60) $${\overset{˜}{U}}^{\prime }{\overset{¯}{W}}^{*}\overset{ˆ}{\beta }-{\overset{˜}{U}}^{\prime }{\overset{¯}{W}}^{0}\beta^{0}={\overset{˜}{U}}^{\prime }P_{{\overset{¯}{W}}^{*}}{\overset{¯}{W}}^{0}\beta^{0}+{\overset{˜}{U}}^{\prime }P_{{\overset{¯}{W}}^{*}}\overset{˜}{U}-{\overset{˜}{U}}^{\prime }{\overset{¯}{W}}^{0}\beta^{0}$$

where $P_{{\overset{¯}{W}}^{*}}={\overset{¯}{W}}^{*}{\left({\overset{¯}{W}}^{{*}^{\prime }}{\overset{¯}{W}}^{*}\right)}^{-1}{\overset{¯}{W}}^{{*}^{\prime }}$. We now analyse the terms on the right hand side of (60). It is more convenient to begin by analysing $\left\|P_{{\overset{¯}{W}}^{*}}\overset{˜}{U}\right\|$. To this end, we define $\sum_{i}$ as the summation over observations $t={\overset{ˆ}{T}}_{i}+1,{\overset{ˆ}{T}}_{i}+2,\ldots ,{\overset{ˆ}{T}}_{i+1}$. First, note ${\left\|P_{{\overset{¯}{W}}^{*}}\overset{˜}{U}\right\|}^{2}={\overset{˜}{U}}^{\prime }P_{{\overset{¯}{W}}^{*}}\overset{˜}{U}$ is the sum of the m+1 terms

(61) $$n_{i,T}={\left(\underset{i}{\sum }{\overset{ˆ}{x}}_{t}{\overset{˜}{u}}_{t}\right)}^{\prime }{\left(\underset{i}{\sum }{\overset{ˆ}{x}}_{t}\overset{\prime }{\underset{t}{\overset{ˆ}{x}}}\right)}^{-1}\left(\underset{i}{\sum }{\overset{ˆ}{x}}_{t}{\overset{˜}{u}}_{t}\right)$$

for i=0,1,…,m. Using Assumptions 8 and 11, it follows that $\sum_{i}{\overset{ˆ}{x}}_{t}{\overset{˜}{u}}_{t}=O_{p}\left(T^{1/2}\right)\sum_{i}{\overset{ˆ}{x}}_{t}{\overset{ˆ}{x}}_{t}^{\prime }=O_{p}\left(T\right)$ and hence that

(62) $${\left\|P_{{\overset{¯}{W}}^{*}}\overset{˜}{U}\right\|}^{2}=O_{p}\left(1\right)\text{.}$$

Now consider the first term on the right hand side of (60). Using (57), it follows that

(63) $$\left\|{\overset{˜}{U}}^{\prime }P_{{\overset{¯}{W}}^{*}}{\overset{¯}{W}}^{0}\beta^{0}\right\|\leq \left\|{\overset{˜}{U}}^{\prime }P_{{\overset{¯}{W}}^{*}}\right\|\cdot \left\|{\overset{¯}{W}}^{0}\beta^{0}\right\|\text{.}$$

Since $W=P_{Z}X$, where X is the original design matrix and $P_{Z}=Z{\left(Z^{\prime }Z\right)}^{-1}Z^{\prime }$ is a projection matrix, it follows from (55)–(56), (8) and Assumptions 8, 9 and 11 that

(64) $$\left\|{\overset{¯}{W}}^{0}\right\|=\left\|W\right\|=\left\|P_{Z}X\right\|\;\leq \;\left\|X\right\|\;\leq \;{\left(\mathrm{tr}X^{\prime }X\right)}^{1/2}=O_{p}\left(T^{1/2}\right)$$

and hence from (62)–(64) that

(65) $$\left\|{\overset{˜}{U}}^{\prime }P_{{\overset{¯}{W}}^{*}}{\overset{¯}{W}}^{0}\beta^{0}\right\|=O_{p}\left(T^{1/2}\right)\text{.}$$

Finally, consider the third term on the right hand side of (60), ${\overset{˜}{U}}^{\prime }{\overset{¯}{W}}^{0}\beta^{0}$. Notice that ${\overset{˜}{U}}^{\prime }{\overset{¯}{W}}^{0}$ consists of m+1 terms, $\sum_{t=T_{i-1}^{0}+1}^{T_{i}^{0}}{\overset{ˆ}{x}}_{t}{\overset{˜}{u}}_{t}$. It can be shown that $\sum_{t=T_{i-1}^{0}+1}^{T_{i}^{0}}{\overset{ˆ}{x}}_{t}{\overset{˜}{u}}_{t}=O_{p}\left(T^{1/2}\right)$ and hence that

(66) $$\left\|{\overset{˜}{U}}^{\prime }{\overset{¯}{W}}^{0}\beta^{0}\right\|=O_{p}\left(T^{1/2}\right)\text{.}$$

Combining (60), (62), (65) and (66), it follows that ${\overset{˜}{U}}^{\prime }{\overset{¯}{W}}^{*}\overset{ˆ}{\beta }-{\overset{˜}{U}}^{\prime }{\overset{¯}{W}}^{0}\beta^{0}=O_{p}\left(T^{1/2}\right)$ and hence that $T^{-1}\left({\overset{˜}{U}}^{\prime }{\overset{¯}{W}}^{*}\overset{ˆ}{\beta }-{\overset{˜}{U}}^{\prime }{\overset{¯}{W}}^{0}\beta^{0}\right)=O_{p}\left(T^{-1/2}\right)=o_{p}\left(1\right)$, which is the desired result. *Part* (ii): Suppose  for some j. In this case, there exists η>0 such that no estimated breaks fall into $\left[T\left(\lambda_{j}^{0}-\eta \right),T\left(\lambda_{j}^{0}+\eta \right)\right]$ with some positive probability ϵ. Suppose further that the interval belongs to the kth estimated regime, then it follows that ${\overset{ˆ}{T}}_{k-1}<T\left(\lambda_{j}^{0}-\eta \right)$ and $T\left(\lambda_{j}^{0}+\eta \right)<\overset{ˆ}{T_{k}}$. Thus $d_{t}={\overset{ˆ}{x}}_{t}^{\prime }\left({\overset{ˆ}{\beta }}_{k}-\beta_{j}^{0}\right)$ for $t\in \left[T\left(\lambda_{j}^{0}-\eta \right),T\lambda_{j}^{0}\right]$, and $d_{t}={\overset{ˆ}{x}}_{t}^{\prime }\left({\overset{ˆ}{\beta }}_{k}-\beta_{j+1}^{0}\right)$ for $t\in \left[T\lambda_{j}^{0}+1,T\left(\lambda_{j}^{0}+\eta \right)\right]$. Using these identities, we obtain

(67) $$\overset{T}{\underset{t=1}{\sum }}d_{t}^{2}\geq \underset{1}{\sum }\overset{2}{\underset{t}{d}}+\underset{2}{\sum }\overset{2}{\underset{t}{d}}$$

where

(68) $$\underset{1}{\sum }\overset{2}{\underset{t}{d}}={\left({\overset{ˆ}{\beta }}_{k}-\beta_{j}^{0}\right)}^{\prime }\left(\underset{1}{\sum }{\overset{ˆ}{x}}_{t}{{\overset{ˆ}{x}}_{t}}^{\prime }\right)\left({\overset{ˆ}{\beta }}_{k}-\beta_{j}^{0}\right)$$

(69) $$\underset{2}{\sum }\overset{2}{\underset{t}{d}}={\left({\overset{ˆ}{\beta }}_{k}-\beta_{j+1}^{0}\right)}^{\prime }\left(\underset{2}{\sum }{\overset{ˆ}{x}}_{t}{{\overset{ˆ}{x}}_{t}}^{\prime }\right)\left({\overset{ˆ}{\beta }}_{k}-\beta_{j+1}^{0}\right)$$

 and $\sum_{1}$ extends over the set $\left\{T\left(\lambda_{j}^{0}-\eta \right)\leq t\leq T\lambda_{j}^{0}\right\}$ and $\sum_{2}$ extends over the set $\left\{T\lambda_{j}^{0}+1\leq t\leq T\left(\lambda_{j}^{0}+\eta \right)\right\}$. At this stage, define $\gamma_{1}$ and $\gamma_{2}$ to be the smallest eigenvalue of $\sum_{1}z_{t}z_{t}^{\prime }$ and $\sum_{2}z_{t}z_{t}^{\prime }$, respectively. Then, since $\sum_{i}{\overset{ˆ}{x}}_{t}{{\overset{ˆ}{x}}_{t}}^{\prime }={\overset{ˆ}{\Delta }}_{T}^{\prime }\left(\sum_{i}z_{t}z_{t}^{\prime }\right){\overset{ˆ}{\Delta }}_{T}$, it follows that^33^

(70) $$\underset{1}{\sum }{d_{t}}^{2}+\underset{2}{\sum }{d_{t}}^{2}={\left({\overset{ˆ}{\Delta }}_{T}\left({\overset{ˆ}{\beta }}_{k}-\overset{0}{\underset{j}{\beta }}\right)\right)}^{\prime }\left(\underset{1}{\sum }z_{t}\overset{\prime }{\underset{t}{z}}\right)\left({\overset{ˆ}{\Delta }}_{T}\left({\overset{ˆ}{\beta }}_{k}-\overset{0}{\underset{j}{\beta }}\right)\right)+{\left({\overset{ˆ}{\Delta }}_{T}\left({\overset{ˆ}{\beta }}_{k}-\overset{0}{\underset{j+1}{\beta }}\right)\right)}^{\prime }\left(\underset{2}{\sum }z_{t}\overset{\prime }{\underset{t}{z}}\right)\left({\overset{ˆ}{\Delta }}_{T}\left({\overset{ˆ}{\beta }}_{k}-\overset{0}{\underset{j+1}{\beta }}\right)\right)\geq \gamma_{1}{\left\|{\overset{ˆ}{\Delta }}_{T}\left({\overset{ˆ}{\beta }}_{k}-\overset{0}{\underset{j}{\beta }}\right)\right\|}^{2}+\gamma_{2}{\left\|{\overset{ˆ}{\Delta }}_{T}\left({\overset{ˆ}{\beta }}_{k}-\overset{0}{\underset{j+1}{\beta }}\right)\right\|}^{2}\geq \left(1/2\right)\cdot \min\left\{\gamma_{1},\gamma_{2}\right\}\cdot {\left\|{\overset{ˆ}{\Delta }}_{T}\left(\overset{0}{\underset{j}{\beta }}-\overset{0}{\underset{j+1}{\beta }}\right)\right\|}^{2}\text{.}$$

Now consider the right hand side of (70). We have

(71) $$\underset{1}{\sum }z_{t}\overset{\prime }{\underset{t}{z}}=\left(T\eta \right)\left(1/T\eta \right)\overset{T\lambda_{j}^{0}}{\underset{t=T\left(\lambda_{j}^{0}-\eta \right)}{\sum }}z_{t}z_{t}^{\prime }=\left(T\eta \right)A_{T}$$

where $A_{T}=\left(1/T\eta \right)\sum_{t=T\left(\lambda_{j}^{0}-\eta \right)}^{T\lambda_{j}^{0}}z_{t}z_{t}^{\prime }$. From Assumption 10, the smallest eigenvalue of $A_{T}$ is bounded away from zero in probability. Thus, the smallest eigenvalue of $\left(T\eta \right)A_{T}$ is of order Tη. Similarly, the smallest eigenvalue of $\sum_{2}z_{t}z_{t}^{\prime }$ is of order Tη. Using these two order statements in (70), it follows that

$$\overset{T}{\underset{t=1}{\sum }}{d_{t}}^{2}\geq \underset{1}{\sum }{d_{t}}^{2}+\underset{2}{\sum }{d_{t}}^{2}\geq TC\cdot {\left\|{\overset{ˆ}{\Delta }}_{T}\left(\overset{0}{\underset{j}{\beta }}-\overset{0}{\underset{j+1}{\beta }}\right)\right\|}^{2}$$

for some C>0 and hence, using ${\overset{ˆ}{\Delta }}_{T}\overset{p}{\to }\Delta_{0}$, that

(72) $$T^{-1}\overset{T}{\underset{t=1}{\sum }}d_{t}^{2}\geq C{\left\|\Delta_{0}\left(\beta_{j}^{0}-\beta_{j+1}^{0}\right)\right\|}^{2}+\xi_{T}$$

where $\xi_{T}=C\left\{\;{\left\|{\overset{ˆ}{\Delta }}_{T}\left(\beta_{j}^{0}-\beta_{j+1}^{0}\right)\right\|}^{2}-{\left\|\Delta_{0}\left(\beta_{j}^{0}-\beta_{j+1}^{0}\right)\right\|}^{2}\;\right\}=o_{p}\left(1\right)$. The desired result then follows from (72) upon recalling that the analysis is premised on an event that occurs with probability ϵ. □

Proof of Theorem 1 Suppose that  for some j in probability. In this case, it follows from (14) and Lemma 1 that

(73) $$\left(1/T\right)\overset{T}{\underset{t=1}{\sum }}{\overset{ˆ}{u}}_{t}^{2}=\left(1/T\right)\overset{T}{\underset{t=1}{\sum }}{\overset{˜}{u}}_{t}^{2}+C\cdot {\left\|\Delta_{0}\left(\beta_{j}^{0}-\beta_{j+1}^{0}\right)\right\|}^{2}+o_{p}\left(1\right)$$

with probability at least as large as $\overset{¯}{\epsilon }>0$. Assumption 9 states that $\Delta_{0}$ is full rank and so ${\left\|\Delta_{0}\left(\beta_{j}^{0}-\beta_{j+1}^{0}\right)\right\|}^{2}>0$. Therefore, (73) conflicts with (13) which must hold for all T with probability one. Therefore, it must follow ${\overset{ˆ}{\lambda }}_{j}\overset{p}{\to }\lambda_{j}^{0}$ for all j. □

Proof of Theorem 2 The general proof strategy is the same as the one employed in Bai and Perron’s (1998) proof of their Proposition 2, although the specific details are naturally different. Following Bai and Perron (1998), we assume (without loss of generality) that there are only 3 break points, that is m=3. Here we present the proof for the middle break fraction, ${\overset{ˆ}{\lambda }}_{2}$. The proof for the end break fractions, ${\overset{ˆ}{\lambda }}_{1}$ and ${\overset{ˆ}{\lambda }}_{3}$, follows along similar lines and is omitted for brevity.^34^ The desired result can be established if it can be shown that for each η>0, there exists C>0 and ϵ>0 such that for large T,

(74) $$P\left(\min\left\{\left[S_{T}\left(T_{1},T_{2},T_{3}\right)-S_{T}\left(T_{1},\overset{0}{\underset{2}{T}},T_{3}\right)\right]/\left(T_{2}^{0}-T_{2}\right)\right\}<0\right)<\eta$$

where the minimum is taken over the set $V_{\epsilon }\left(C\right)=\left\{\left(T_{1},T_{2},T_{3}\right):\left|T_{i}-T_{i}^{0}\right|\leq \epsilon T,\;i=1,2,3\text{ but }T_{2}-T_{2}^{0}<-C\right\}$. Define ${\mathrm{SSR}}_{1}=S_{T}\left(T_{1},T_{2},T_{3}\right),{\mathrm{SSR}}_{2}=S_{T}\left(T_{1},T_{2}^{0},T_{3}\right)$ and ${\mathrm{SSR}}_{3}=S_{T}\left(T_{1},T_{2},T_{2}^{0},T_{3}\right)$. Using these definitions, we have

(75) $$S_{T}\left(T_{1},T_{2},T_{3}\right)-S_{T}\left(T_{1},T_{2}^{0},T_{3}\right)=\left({\mathrm{SSR}}_{1}-{\mathrm{SSR}}_{3}\right)-\left({\mathrm{SSR}}_{2}-{\mathrm{SSR}}_{3}\right)\text{.}$$

To analyse the terms on the right hand side of (75), it is useful to define the 2SLS estimators in the four break model and emphasize the sub-samples upon which some of these estimators are based. Let $\left({\overset{ˆ}{\beta }}_{1}^{*},{\overset{ˆ}{\beta }}_{2}^{*},{\overset{ˆ}{\beta }}_{△},{\overset{ˆ}{\beta }}_{3}^{*},{\overset{ˆ}{\beta }}_{4}^{*}\right)$ denote the 2SLS estimators of the regression coefficients in the five regimes of the four break model associated with the partition $\left(T_{1},T_{2},T_{2}^{0},T_{3}\right)$. Note that ${\overset{ˆ}{\beta }}_{2}^{*}$ is based on observations $T_{1}+1,\ldots ,T_{2}$; ${\overset{ˆ}{\beta }}_{△}$ is based on observations $T_{2}+1,\ldots ,T_{2}^{0};\overset{ˆ}{\beta_{3}^{*}}$ is based on observations $T_{2}^{0}+1,\ldots ,T_{3}$. Now define $\overset{¯}{W}$ to be the diagonal partition of W at $\left(T_{1},T_{2},T_{3}\right),\overset{˜}{W}$ is the diagonal partition of W at $\left(T_{1},T_{2}^{0},T_{3}\right),W_{△}={\left(0_{p\times T_{2}},{\overset{ˆ}{x}}_{T_{2}+1},\ldots ,{\overset{ˆ}{x}}_{T_{2}^{0}},0_{p\times \left(T-T_{2}^{0}\right)}\right)}^{\prime }$ and $M_{\overset{¯}{W}}=I_{T}-\overset{¯}{W}{\left({\overset{¯}{W}}^{\prime }\overset{¯}{W}\right)}^{-1}{\overset{¯}{W}}^{\prime }$. It can be shown that^35^

(76) $${\mathrm{SSR}}_{1}-{\mathrm{SSR}}_{3}={\left(\overset{ˆ}{\beta_{3}^{*}}-{\overset{ˆ}{\beta }}_{△}\right)}^{\prime }W_{△}^{\prime }M_{\overset{¯}{W}}W_{△}\left(\overset{ˆ}{\beta_{3}^{*}}-{\overset{ˆ}{\beta }}_{△}\right)$$

(77) $${\mathrm{SSR}}_{2}-{\mathrm{SSR}}_{3}={\left(\overset{ˆ}{\beta_{2}^{*}}-{\overset{ˆ}{\beta }}_{△}\right)}^{\prime }W_{△}^{\prime }M_{\overset{˜}{W}}W_{△}\left(\overset{ˆ}{\beta_{2}^{*}}-{\overset{ˆ}{\beta }}_{△}\right)\text{.}$$

 From (76)–(77) and $W_{△}^{\prime }M_{\overset{˜}{W}}W_{△}\leq W_{△}^{\prime }W_{△}$, it follows that

(78) $${\mathrm{SSR}}_{1}-{\mathrm{SSR}}_{2}\geq {\left(\overset{ˆ}{\beta_{3}^{*}}-{\overset{ˆ}{\beta }}_{△}\right)}^{\prime }W_{△}^{\prime }M_{\overset{¯}{W}}W_{△}\left(\overset{ˆ}{\beta_{3}^{*}}-{\overset{ˆ}{\beta }}_{△}\right)-{\left(\overset{ˆ}{\beta_{2}^{*}}-{\overset{ˆ}{\beta }}_{△}\right)}^{\prime }W_{△}^{\prime }W_{△}\left(\overset{ˆ}{\beta_{2}^{*}}-{\overset{ˆ}{\beta }}_{△}\right)\text{.}$$

Substituting for $M_{\overset{¯}{W}}$ in (78) and dividing both sides by $T_{2}^{0}-T_{2}$, we obtain

(79) $$\frac{{\mathrm{SSR}}_{1}-{\mathrm{SSR}}_{2}}{T_{2}^{0}-T_{2}}\;\geq \;N_{1}-N_{2}-N_{3}$$

where

(80) $$N_{1}={\left(\overset{ˆ}{\beta_{3}^{*}}-{\overset{ˆ}{\beta }}_{△}\right)}^{\prime }\left[{\left(T_{2}^{0}-T_{2}\right)}^{-1}W_{△}^{\prime }W_{△}\right]\left(\overset{ˆ}{\beta_{3}^{*}}-{\overset{ˆ}{\beta }}_{△}\right)$$

(81) $$N_{2}={\left(\overset{ˆ}{\beta_{3}^{*}}-{\overset{ˆ}{\beta }}_{△}\right)}^{\prime }\left[{\left(T_{2}^{0}-T_{2}\right)}^{-1}W_{△}^{\prime }\overset{¯}{W}\right]{\left[T^{-1}{\overset{¯}{W}}^{\prime }\overset{¯}{W}\right]}^{-1}\left[T^{-1}{\overset{¯}{W}}^{\prime }W_{△}\right]\left(\overset{ˆ}{\beta_{3}^{*}}-{\overset{ˆ}{\beta }}_{△}\right)$$

(82) $$N_{3}={\left(\overset{ˆ}{\beta_{2}^{*}}-{\overset{ˆ}{\beta }}_{△}\right)}^{\prime }\left[{\left(T_{2}^{0}-T_{2}\right)}^{-1}W_{△}^{\prime }W_{△}\right]\left(\overset{ˆ}{\beta_{2}^{*}}-{\overset{ˆ}{\beta }}_{△}\right)\text{.}$$

 It can be shown that under our assumptions $N_{1}$ is the dominant term and, as a consequence, that $\left[\left({\mathrm{SSR}}_{1}-{\mathrm{SSR}}_{2}\right)/\left(T_{2}^{0}-T_{2}\right)\right]>0$ over $V_{\epsilon }\left(C\right)$ with large probability which proves (74). □

Proof of Theorem 3 For notational brevity, set $\overset{ˆ}{\beta }=\overset{ˆ}{\beta }\left({\left\{{\overset{ˆ}{T}}_{i}\right\}}_{i=1}^{m}\right)$. It can be shown that

(83) $$T^{1/2}\left(\overset{ˆ}{\beta }-\beta^{0}\right)={\left(T^{-1}{\overset{¯}{W}}^{{*}^{\prime }}{\overset{¯}{W}}^{*}\right)}^{-1}T^{-1/2}{\overset{¯}{W}}^{{*}^{\prime }}\left[\overset{˜}{U}+\left({\overset{¯}{W}}^{0}-{\overset{¯}{W}}^{*}\right)\beta^{0}\right]$$

Theorem 2 implies that ${\overset{ˆ}{T}}_{i}-T_{i}^{0}=O_{p}\left(1\right)$ for all i. Therefore, the summation ${\overset{¯}{W}}^{{*}^{\prime }}{\overset{¯}{W}}^{0}-{\overset{¯}{W}}^{{*}^{\prime }}{\overset{¯}{W}}^{*}$ involves a bounded number of terms with probability one, and so

(84) $$T^{1/2}\left(\overset{ˆ}{\beta }-\beta^{0}\right)={\left(T^{-1}{\overset{¯}{W}}^{{*}^{\prime }}{\overset{¯}{W}}^{*}\right)}^{-1}T^{-1/2}{\overset{¯}{W}}^{{*}^{\prime }}\overset{˜}{U}+o_{p}\left(1\right)\text{.}$$

The addition and subtraction of ${\left(T^{-1}{\overset{¯}{W}}^{0^{\prime }}{\overset{¯}{W}}^{0}\right)}^{-1}T^{-1/2}{\overset{¯}{W}}^{0^{\prime }}\overset{˜}{U}$ to the right hand side of (84) and some rearrangement yields

(85) $$T^{1/2}\left(\overset{ˆ}{\beta }-\beta^{0}\right)={\left(T^{-1}{\overset{¯}{W}}^{0^{\prime }}{\overset{¯}{W}}^{0}\right)}^{-1}T^{-1/2}{\overset{¯}{W}}^{0^{\prime }}\overset{˜}{U}+{\left(T^{-1}{\overset{¯}{W}}^{0^{\prime }}{\overset{¯}{W}}^{0}\right)}^{-1}\left(T^{-1}{\overset{¯}{W}}^{0^{\prime }}{\overset{¯}{W}}^{0}-T^{-1}{\overset{¯}{W}}^{{*}^{\prime }}{\overset{¯}{W}}^{*}\right){\left(T^{-1}{\overset{¯}{W}}^{{*}^{\prime }}{\overset{¯}{W}}^{*}\right)}^{-1}T^{-1/2}{\overset{¯}{W}}^{0^{\prime }}\overset{˜}{U}+{\left(T^{-1}{\overset{¯}{W}}^{{*}^{\prime }}{\overset{¯}{W}}^{*}\right)}^{-1}T^{-1/2}\left({\overset{¯}{W}}^{{*}^{\prime }}-{\overset{¯}{W}}^{0^{\prime }}\right)\overset{˜}{U}+o_{p}\left(1\right)\text{.}$$

Since ${\overset{ˆ}{T}}_{i}-T_{i}^{0}=O_{p}\left(1\right)$ for all i, it follows from (85) using Assumptions 9 and 11 that

(86) $$T^{1/2}\left(\overset{ˆ}{\beta }-\beta^{0}\right)={\left(T^{-1}{\overset{¯}{W}}^{0^{\prime }}{\overset{¯}{W}}^{0}\right)}^{-1}T^{-1/2}{\overset{¯}{W}}^{0^{\prime }}\overset{˜}{U}+o_{p}\left(1\right)\text{.}$$

Given the block diagonal structure of ${\overset{¯}{W}}^{0^{\prime }}{\overset{¯}{W}}^{0}$, the coefficient vector of the i-th regime can be written as

(87) $$T^{1/2}\left({\overset{ˆ}{\beta }}_{i}-\beta_{i}^{0}\right)={\left(\frac{1}{T}\underset{i_{0}}{\sum }{\overset{ˆ}{x}}_{t}\overset{\prime }{\underset{t}{\overset{ˆ}{x}}}\right)}^{-1}T^{-1/2}\underset{i_{0}}{\sum }{\overset{ˆ}{x}}_{t}{\overset{˜}{u}}_{t}+o_{p}\left(1\right)\text{,}$$

where $\sum_{i_{0}}$ implies summing over terms $t=T_{i-1}^{0}+1,\ldots ,T_{i}^{0}$. The result then follows from (87) under our assumptions. □

Proof of Theorem 4 The F-statistic can be written as

(88) $$F_{T}\left(\lambda_{1},\ldots ,\lambda_{k};p\right)=F_{T}^{*}/\left[kp\;{\left(T-\left(k+1\right)p\right)}^{-1}{\mathrm{SSR}}_{k}\right]$$

where $F_{T}^{*}={\mathrm{SSR}}_{0}-{\mathrm{SSR}}_{k}$. We first consider the limiting behaviour of $F_{T}^{*}$. To this end, we define $D^{R}\left(i,j\right)$ to be the sum of squared residuals from the restricted model using observations from i to j, that is, from $T_{i-1}+1$ to $T_{j}$, and $D^{U}\left(i,j\right)$ to be the corresponding sum of squared residuals for the unrestricted model. Using this notation, we can write $F_{T}^{*}$ as follows^36^:

(89) $$F_{T}^{*}=D^{R}\left(1,k+1\right)-\overset{k+1}{\underset{i=1}{\sum }}D^{U}\left(i,i\right)=\overset{k}{\underset{i=1}{\sum }}\left[D^{R}\left(1,i+1\right)-D^{R}\left(1,i\right)-D^{U}\left(i+1,i+1\right)\right]$$

(90) $$=\overset{k}{\underset{i=1}{\sum }}F_{T,i}\text{,}\;\text{say .}$$

 It can be shown that

(91) $$F_{T,i}={\left\|\left(I-P_{W_{1,i+1}}\right){\overset{˜}{U}}_{1,i+1}\right\|}^{2}-{\left\|\left(I-P_{W_{1,i}}\right){\overset{˜}{U}}_{1,i}\right\|}^{2}-{\left\|\left(I-P_{W_{i+1}}\right){\overset{˜}{U}}_{i+1}\right\|}^{2}=-S_{i+1}^{\prime }H_{i+1}^{-1}S_{i+1}+S_{i}^{\prime }H_{i}^{-1}S_{i}+A_{i}$$

where $S_{j}=W_{1,j}^{\prime }{\overset{˜}{U}}_{1,j},H_{j}=W_{1,j}^{\prime }W_{1,j}$ and $A_{i}={\left(S_{i+1}-S_{i}\right)}^{\prime }{\left(H_{i+1}-H_{i}\right)}^{-1}\left(S_{i+1}-S_{i}\right)$. Assumptions 8, 12 and 13 together ensure that the following version of the uniform version of the multivariate functional central limit theorem (FCLT) in Wooldridge and White (1988) holds: $T^{-1/2}\sum_{t=1}^{\left[Tr\right]}h_{t}⟹\left(\Omega^{1/2}⊗Q_{ZZ}^{1/2}\right)B_{n}\left(r\right)$ where $B_{n}\left(r\right)$ is an n×1 standard Brownian motion with $n=q\times \left(p_{1}+1\right)$. To explore the implications of this distributional result further, let $B\left(r\right)={\left[B_{1}{\left(r\right)}^{\prime },B_{2}{\left(r\right)}^{\prime },\ldots ,B_{p+1}{\left(r\right)}^{\prime }\right]}^{\prime }$ where $B_{i}{\left(r\right)}^{\prime }$ is q×1, and $\Omega^{1/2}={\left[N_{1},\;N_{2}\right]}^{\prime }$ where $N_{1}^{\prime }$ is a 1×(p+1) vector whose ith element is $N_{1,i}$, and $N_{2}^{\prime }$ is p×(p+1). Note that, since $\Omega^{1/2}$ is symmetric,

(92) $$\Omega =\left[\;\begin{array}{cc} N_{1}^{\prime }N_{1} & N_{1}^{\prime }N_{2}\\ N_{2}^{\prime }N_{1} & N_{2}^{\prime }N_{2} \end{array}\;\right]=\left[\;\begin{array}{cc} \sigma^{2} & \gamma^{\prime }\\ \gamma & \Sigma \end{array}\;\right]$$

where the second and third matrices are partitioned conformably. It follows from the FCLT above that $T^{-1/2}\sum_{t=1}^{\left[Tr\right]}z_{t}u_{t}⟹\left(N_{1}^{\prime }⊗Q_{ZZ}^{1/2}\right)B\left(r\right)=Q_{ZZ}^{1/2}{\overset{˜}{D}}^{*}\left(r\right),\text{ say}$ and $T^{-1/2}\sum_{t=1}^{\left[Tr\right]}z_{t}v_{t}^{\prime }⟹Q_{ZZ}^{1/2}B^{\mathrm{mat}}\left(r\right)N_{2}=Q_{ZZ}^{1/2}D^{*}\left(r\right),\text{ say}$ where $\mathrm{vec}\left(B^{\mathrm{mat}}\left(r\right)\right)=B\left(r\right)$. Further note ${\left({\Delta_{0}}^{\prime }Q_{ZZ}^{1/2}\right)}^{\prime }\times {\left({\Delta_{0}}^{\prime }Q_{ZZ}\Delta_{0}\right)}^{-1}\left({\Delta_{0}}^{\prime }Q_{ZZ}^{1/2}\right)=C^{\prime }\Lambda C$ where C is an orthogonal matrix and Λ is a diagonal matrix, whose first p diagonal elements are one and the remaining q−p equal to zero. Using these definitions, it can be shown that

$$S_{i+1}^{\prime }H_{i+1}^{-1}S_{i+1}⟹\lambda_{i+1}^{-1}{\left(\Lambda C{\overset{˜}{D}}^{*}\left(\lambda_{i+1}\right)+\Lambda C\left[D^{*}\left(\lambda_{i+1}\right)-\lambda_{i+1}D^{*}\left(1\right)\right]{\overset{¯}{\beta }}_{0}\right)}^{\prime }\left(\Lambda C{\overset{˜}{D}}^{*}\left(\lambda_{i+1}\right)+\Lambda C\left[D^{*}\left(\lambda_{i+1}\right)-\lambda_{i+1}D^{*}\left(1\right)\right]{\overset{¯}{\beta }}_{0}\right)$$

$$A_{i}⟹{\left(\lambda_{i+1}-\lambda_{i}\right)}^{-1}{\left[\Lambda C\left({\overset{˜}{D}}^{*}\left(\lambda_{i+1}\right)-{\overset{˜}{D}}^{*}\left(\lambda_{i}\right)\right)+\Lambda C\left(D^{*}\left(\lambda_{i+1}\right)-D^{*}\left(\lambda_{i}\right)-\lambda_{i+1}D^{*}\left(1\right)+\lambda_{i}D^{*}\left(1\right)\right){\overset{¯}{\beta }}_{0}\right]}^{\prime }\left[\Lambda C\left({\overset{˜}{D}}^{*}\left(\lambda_{i+1}\right)-{\overset{˜}{D}}^{*}\left(\lambda_{i}\right)\right)+\Lambda C\left(D^{*}\left(\lambda_{i+1}\right)-D^{*}\left(\lambda_{i}\right)-\lambda_{i+1}D^{*}\left(1\right)+\;\lambda_{i}D^{*}\left(1\right)\right){\overset{¯}{\beta }}_{0}\right]\text{.}$$

 Now define $D_{i}=\Lambda CD^{*}\left(\lambda_{i}\right),{\overset{˜}{D}}_{i}=\Lambda C{\overset{˜}{D}}^{*}\left(\lambda_{i}\right)$ and $D_{1}=\Lambda CD^{*}\left(1\right)$. Then it can be shown that

(93) $$F_{T,i}⟹{\left\{\lambda_{i}\lambda_{i+1}\left(\lambda_{i+1}-\lambda_{i}\right)\right\}}^{-1}{\left\|\left[\lambda_{i+1}{\overset{˜}{D}}_{i}-\lambda_{i}{\overset{˜}{D}}_{i+1}\right]+\left[\lambda_{i+1}D_{i}-\lambda_{i}D_{i+1}\right]{\overset{¯}{\beta }}_{0}\right\|}^{2}$$

where ${\overset{¯}{\beta }}_{0}$ is the common value of $\beta_{i}^{0}$ under the null. It can be shown that ${\left(T-\left(k+1\right)p\right)}^{-1}{\mathrm{SSR}}_{k}\overset{p}{\to }\sigma^{2}+2\gamma^{\prime }{\overset{¯}{\beta }}_{0}+{\overset{¯}{\beta }}_{0}^{\prime }\Sigma {\overset{¯}{\beta }}_{0}$. The desired result then follows after some additional manipulations. □

Proof of Theorem 5 Consider first the equation given in Box II Box II

(94) $${\overset{˜}{F}}_{T}\left(i;l\right)=\frac{\left\{{\mathrm{SSR}}_{l}\left({\overset{ˆ}{T}}_{1},\ldots ,{\overset{ˆ}{T}}_{l}\right)-\underset{\tau \in \Lambda_{i,\eta }}{\inf}{\mathrm{SSR}}_{l+1}\left({\overset{ˆ}{T}}_{1},\ldots ,{\overset{ˆ}{T}}_{i-1},\tau ,{\overset{ˆ}{T}}_{i},\ldots ,{\overset{ˆ}{T}}_{l}\right)\right\}}{{\overset{ˆ}{\sigma }}_{i}^{2}}$$

 for a given i. Defining $S_{T}\left(i,j\right)$ to be the minimized sum of squared residuals for the segment containing observations from i to j, we can write ${\overset{˜}{F}}_{T}\left(i;l\right)$ as in Box III Box III

(95) $${\overset{˜}{F}}_{T}\left(i;l\right)=\underset{\tau \in \Lambda_{i,\eta }}{\sup}\frac{\left\{S_{T}\left({\overset{ˆ}{T}}_{i-1}+1,{\overset{ˆ}{T}}_{i}\right)-S_{T}\left({\overset{ˆ}{T}}_{i-1}+1,\tau \right)-S_{T}\left(\tau +1,{\overset{ˆ}{T}}_{i}\right)\right\}}{\overset{2}{\underset{i}{\overset{ˆ}{\sigma }}}}$$

. Under our assumptions, it can be shown that ${\overset{ˆ}{\sigma }}_{i}^{2}\overset{p}{\to }\sigma_{i}^{2}+2\gamma_{i}^{\prime }\beta_{i}^{0}+\beta_{i}^{0^{\prime }}\Sigma_{i}\beta_{i}^{0}$. Using the latter and Theorem 2, it follows the equation given in Box IV Box IV

(96) $${\overset{˜}{F}}_{T}\left(i;l\right)=\underset{\tau \in \Lambda_{i,\eta }^{0}}{\sup}\left\{\;\frac{S_{T}\left(\overset{0}{\underset{i-1}{T}}+1,T_{i}^{0}\right)-S_{T}\left(T_{i-1}^{0}+1,\tau \right)-S_{T}\left(\tau +1,T_{i}^{0}\right)}{\sigma^{2}+2\gamma^{\prime }\overset{0}{\underset{i}{\beta }}+\beta_{i}^{0\prime }\Sigma \beta_{i}^{0}}\;\right\}+o_{p}\left(1\right)$$

where $\Lambda_{i,\eta }^{0}=\left\{\tau :T_{i-1}^{0}+\left(T_{i}^{0}-T_{i-1}^{0}\right)\eta \leq \tau \leq T_{i}^{0}-\left(T_{i}^{0}-T_{i-1}^{0}\right)\eta \right\}$. . After some manipulations, it can be shown that ${\overset{˜}{F}}_{T}\left(i;l\right)\;⟹\;\sup_{\eta \leq \mu <1-\eta }{\left\|W\left(\mu \right)-\mu W\left(1\right)\right\|}^{2}\;/\;\mu \left(1-\mu \right)$, and then the result follows. □

Proof of Theorem 6 We start by considering the limiting behaviour of the constituents of

$${\mathrm{Wald}}_{T}=T\overset{ˆ}{\beta }{\left({\overset{¯}{T}}_{k}\right)}^{\prime }R_{k}^{\prime }{\left[R_{k}{\overset{ˆ}{V}}_{W}\left({\overset{¯}{T}}_{k}\right)R_{k}^{\prime }\right]}^{-1}R_{k}\overset{ˆ}{\beta }\left({\overset{¯}{T}}_{k}\right)\text{.}$$

It can be shown that

(97) $$T^{1/2}\left({\overset{ˆ}{\beta }}_{i}-{\overset{¯}{\beta }}_{0}\right)={\left\{\left(\lambda_{i}-\lambda_{i-1}\right)\Delta_{0}^{\prime }Q_{ZZ}\Delta_{0}\right\}}^{-1}\Delta_{0}^{\prime }T^{-1/2}\underset{i}{\sum }z_{t}\left(u_{t}+\overset{\prime }{\underset{t}{v}}{\overset{¯}{\beta }}_{0}\right)-{\left(\Delta_{0}^{\prime }Q_{ZZ}\Delta_{0}\right)}^{-1}\Delta_{0}^{\prime }T^{-1/2}\overset{T}{\underset{t=1}{\sum }}z_{t}v_{t}^{\prime }{\overset{¯}{\beta }}_{0}+o_{p}\left(1\right)$$

where ${\overset{¯}{\beta }}_{0}$ is the common value of $\left\{\beta_{i}^{0};\;i=1,2,\ldots ,k+1\right\}$ under $H_{0}$. Assumptions 8 and 16 together ensure that the following version of the uniform version of the multivariate functional central limit theorem in Wooldridge and White (1988) holds: $T^{-1/2}\sum_{t=1}^{\left[Tr\right]}h_{t}⟹V^{1/2}B_{n}\left(r\right)$ where $B_{n}\left(r\right)$ is an n×1 standard Brownian motion with $n=q\times \left(p_{1}+1\right)$. Partition $V^{1/2}={\left[{\overset{˜}{N}}_{1},{\overset{˜}{N}}_{2}\right]}^{\prime }$ where ${\overset{˜}{N}}_{1}$ is a (p+1)×1 vector, and ${\overset{˜}{N}}_{2}^{\prime }$ is (p+1)×p. It then follows that

$$T^{1/2}\left({\overset{ˆ}{\beta }}_{i}-{\overset{¯}{\beta }}_{0}\right)\Rightarrow {\left(\Delta_{0}^{\prime }Q_{ZZ}\Delta_{0}\right)}^{-1}\left\{{\left(\lambda_{i}-\lambda_{i-1}\right)}^{-1}\Delta_{0}^{\prime }\left[{\overset{˜}{N}}_{1}^{\prime }+\left({\overset{¯}{\beta }}_{0}^{\prime }⊗I_{q}\right){\overset{˜}{N}}_{2}^{\prime }\right]\left[B_{n}\left(\lambda_{i}\right)-B_{n}\left(\lambda_{i-1}\right)\right]-\left[\Delta_{0}^{\prime }\left({\overset{¯}{\beta }}_{0}^{\prime }⊗I_{q}\right){\overset{˜}{N}}_{2}^{\prime }\right]B_{n}\left(1\right)\right\}$$

and hence,

(98) $$T^{1/2}\left({\overset{ˆ}{\beta }}_{i+1}-{\overset{ˆ}{\beta }}_{i}\right)\Rightarrow {\left(\Delta_{0}^{\prime }Q_{ZZ}\Delta_{0}\right)}^{-1}\left\{{\left(\lambda_{i+1}-\lambda_{i}\right)}^{-1}\Delta_{0}^{\prime }\left[{\overset{˜}{N}}_{1}^{\prime }+\left({\overset{¯}{\beta }}_{0}^{\prime }⊗I_{q}\right){\overset{˜}{N}}_{2}^{\prime }\right]\left[B_{n}\left(\lambda_{i+1}\right)-B_{n}\left(\lambda_{i}\right)\right]-{\left(\lambda_{i}-\lambda_{i-1}\right)}^{-1}\Delta_{0}^{\prime }\left[{\overset{˜}{N}}_{1}^{\prime }+\left({\overset{¯}{\beta }}_{0}^{\prime }⊗I_{q}\right){\overset{˜}{N}}_{2}^{\prime }\right]\left[B_{n}\left(\lambda_{i}\right)-B_{n}\left(\lambda_{i-1}\right)\right]\right\}\text{.}$$

From (98), it follows that

(99) $$T^{1/2}R_{m}\overset{ˆ}{\beta }\Rightarrow \left({\overset{˜}{R}}_{m}⊗I_{p}\right)\left\{C_{m}^{-1}⊗{\left(\Delta_{0}^{\prime }Q_{ZZ}\Delta_{0}\right)}^{-1}A\right\}\overset{¯}{B}$$

where $A=\Delta_{0}^{\prime }\left[I_{q},{\overset{¯}{\beta }}_{0}^{\prime }⊗I_{q}\right]{\left[{\overset{˜}{N}}_{1},{\overset{˜}{N}}_{2}\right]}^{\prime }$, $C_{m}=\mathrm{diag}\left[\lambda_{1},\lambda_{2}-\lambda_{1},\ldots ,\lambda_{m}-\lambda_{m-1},1-\lambda_{m}\right],\overset{¯}{B}={\left[B{\left(\lambda_{1}\right)}^{\prime },{\left\{B\left(\lambda_{2}\right)-B\left(\lambda_{1}\right)\right\}}^{\prime },\ldots ,{\left\{B\left(\lambda_{m}\right)-B\left(\lambda_{m-1}\right)\right\}}^{\prime },{\left\{B\left(1\right)-B\left(\lambda_{m}\right)\right\}}^{\prime }\right]}^{\prime }$, and B(⋅) is a $p_{1}\times 1$ vector of independent Brownian motions. Under our conditions, we have

(100) $${\overset{ˆ}{V}}_{W}\left(i\right)\overset{p}{\to }{\left(\lambda_{i}-\lambda_{i-1}\right)}^{-1}{\left(\Delta_{0}^{\prime }Q_{ZZ}\Delta_{0}\right)}^{-1}H{\left(\Delta_{0}^{\prime }Q_{ZZ}\Delta_{0}\right)}^{-1}$$

where H is the common value of $H_{i}$ under $H_{0}$, and so

(101) $${\overset{ˆ}{V}}_{W}\overset{p}{\to }\left({\overset{˜}{R}}_{m}⊗I_{p}\right)\left\{C_{m}^{-1}⊗{\left(\Delta_{0}^{\prime }Q_{ZZ}\Delta_{0}\right)}^{-1}H{\left(\Delta_{0}^{\prime }Q_{ZZ}\Delta_{0}\right)}^{-1}\right\}\left({\overset{˜}{R}}_{m}^{\prime }⊗I_{p}\right)\text{.}$$

If we write $\overset{˜}{A}=A^{\prime }{\left(\Delta_{0}^{\prime }Q_{ZZ}\Delta_{0}\right)}^{-1}$ then it follows from (99), (101) and $H=AA^{\prime }$ that

(102) $${\mathrm{Wald}}_{T}\Rightarrow {\overset{¯}{B}}^{\prime }\left\{C_{m}^{-1}{\overset{˜}{R}}_{m}^{\prime }{\left[{\overset{˜}{R}}_{m}C_{m}^{-1}{\overset{˜}{R}}_{m}^{\prime }\right]}^{-1}{\overset{˜}{R}}_{m}C_{m}^{-1}⊗\overset{˜}{A}{\left({\overset{˜}{A}}^{\prime }\overset{˜}{A}\right)}^{-1}{\overset{˜}{A}}^{\prime }\right\}\overset{¯}{B}∼{\overset{¯}{B}}^{\prime }\left\{C_{m}^{-1}{\overset{˜}{R}}_{m}^{\prime }{\left[{\overset{˜}{R}}_{m}C_{m}^{-1}{\overset{˜}{R}}_{m}^{\prime }\right]}^{-1}{\overset{˜}{R}}_{m}C_{m}^{-1}⊗I_{p}\right\}\overset{¯}{B}\text{.}$$

The result then follows from the Continuous Mapping Theorem and (102). □

Proof of Theorem 7 Define $\tau =T_{i-1}^{0}+\mu \left(T_{i}^{0}-T_{i-1}^{0}\right)$. Using similar arguments to the proof of Theorem 6 and imposing the null hypothesis, we obtain

$$T^{1/2}R_{1}\left({\overset{ˆ}{\beta }}_{1}\left(\tau ;i\right)-{\overset{ˆ}{\beta }}_{2}\left(\tau ;i\right)\right)\Rightarrow {\left[\mu \left(1-\mu \right)\right]}^{-1}{\left[\Delta_{0}^{\prime }Q_{ZZ}^{\left(i\right)}\Delta_{0}\right]}^{-1}A_{i}\left[B\left(\mu \right)-\mu B\left(1\right)\right]$$

where $A_{i}=\Delta_{0}^{\prime }\left[I_{q},{\beta_{i}^{0}}^{\prime }⊗I_{q}\right]{\left[{\overset{˜}{N}}_{1},{\overset{˜}{N}}_{2}\right]}^{\prime }$, and ${\overset{ˆ}{V}}_{W}\left(\tau ;i\right)\overset{p}{\to }{\left[\mu \left(1-\mu \right)\right]}^{-1}\;{\left[\Delta_{0}^{\prime }Q_{ZZ}^{\left(i\right)}\Delta_{0}\right]}^{-1}A_{i}A_{i}^{\prime }{\left[\Delta_{0}^{\prime }Q_{ZZ}^{\left(i\right)}\Delta_{0}\right]}^{-1}$. The result then follows by similar arguments to the proof of Theorem 5. □

Proof of Theorem 8 *Part* (i): The proof follows similar lines to Theorem 1. We first state the analogs to Lemma 1(a)–(b), the proof of which can be found in the Supplemental Appendix, and then use them to deduce the desired result. □

Lemma B.1 *Under the conditions of* Theorem 8(i)*, we have the following.*

(a) $T^{-1}\sum_{t=1}^{T}{\overset{˜}{u}}_{t}d_{t}=o_{p}\left(1\right)$*.*
(b1) *If  for some* j*, and* $\lambda_{j}^{0}\in \left(\pi_{i}^{0},\pi_{i+1}^{0}\right)$*, then*
  $$\underset{T\to \infty }{lim sup}\;P\left(T^{-1}\overset{T}{\underset{t=1}{\sum }}{d_{t}}^{2}>C{\left\|\overset{\left(i+1\right)}{\underset{0}{\Delta }}\left(\overset{0}{\underset{j}{\beta }}-\overset{0}{\underset{j+1}{\beta }}\right)+\overset{\prime }{\underset{T}{\xi }}\right\|}^{2}\right)>\overset{¯}{\epsilon }$$
  *for some* C>0 *and* $\overset{¯}{\epsilon }>0$*, where* $\xi_{T}^{\prime }=o_{p}\left(1\right)$*.*
(b2) *If  for some* j*, and* $\lambda_{j}^{0}=\pi_{i}^{0}$ *for some* i*, then*
  $$\underset{T\to \infty }{lim sup}\;P\left(T^{-1}\overset{T}{\underset{t=1}{\sum }}{d_{t}}^{2}>C\left\{{\left\|\overset{\left(i\right)}{\underset{0}{\Delta }}\left({\overset{ˆ}{\beta }}_{k}-\overset{0}{\underset{j}{\beta }}\right)\right\|}^{2}+{\left\|\overset{\left(i+1\right)}{\underset{0}{\Delta }}\left({\overset{ˆ}{\beta }}_{k}-\overset{0}{\underset{j+1}{\beta }}\right)\right\|}^{2}+\overset{\prime \prime }{\underset{T}{\xi }}\right\}\right)>\overset{¯}{\epsilon }$$
  *for some* C>0 *and* $\overset{¯}{\epsilon }>0$*, where* $\xi_{T}^{\prime \prime }=o_{p}\left(1\right)$*.*

We now use this result to prove Theorem 8(i). Suppose that  for some j. In this case it follows from (14) and Lemma A.1 that with probability $\overset{¯}{\epsilon }>0$:

• Case 1: If for some $i,\pi_{i}^{0}<\lambda_{j}^{0}<\pi_{i+1}^{0}$
  $$T^{-1}\overset{T}{\underset{t=1}{\sum }}{\overset{ˆ}{u}}_{t}^{2}\;>T^{-1}\overset{T}{\underset{t=1}{\sum }}{\overset{˜}{u}}_{t}^{2}+C{\left\|\Delta_{0}^{\left(i+1\right)}\left(\beta_{j}^{0}-\beta_{j+1}^{0}\right)\right\|}^{2}+o_{p}\left(1\right)$$
• Case 2: If $\pi_{i}^{0}=\lambda_{j}^{0}$ for some i
  $$T^{-1}\overset{T}{\underset{t=1}{\sum }}{\overset{ˆ}{u}}_{t}^{2}>T^{-1}\overset{T}{\underset{t=1}{\sum }}{\overset{˜}{u}}_{t}^{2}+C\left\{{\left\|\Delta_{0}^{\left(i\right)}\left({\overset{ˆ}{\beta }}_{k}-\beta_{j}^{0}\right)\right\|}^{2}+{\left\|\Delta_{0}^{\left(i+1\right)}\left({\overset{ˆ}{\beta }}_{k}-\beta_{j+1}^{0}\right)\right\|}^{2}\right\}+o_{p}\left(1\right)\text{.}$$

Thus, we have the following.

• Case 1: Assumption 19(ii) and $\beta_{j}^{0}\neq \beta_{j+1}^{0}$ implies ${\left\|\Delta_{0}^{\left(i+1\right)}\left(\beta_{j}^{0}-\beta_{j+1}^{0}\right)\right\|}^{2}>0$, which gives the result as in the proof of Theorem 1.
• Case 2: Now as $\beta_{j}^{0}\neq \beta_{j+1}^{0}$ and $\Delta_{0}^{\left(i\right)},\;\Delta_{0}^{\left(i+1\right)}$ are rank p from Assumption 19(ii), it must follow that ${\left\|\Delta_{0}^{\left(i\right)}\left({\overset{ˆ}{\beta }}_{k}-\beta_{j}^{0}\right)\right\|}^{2}+{\left\|\Delta_{0}^{\left(i+1\right)}\left({\overset{ˆ}{\beta }}_{k}-\beta_{j+1}^{0}\right)\right\|}^{2}>0$ with probability one, which gives the result via the same argument as in Theorem 1.

*Part* (ii): The general proof strategy is the same as that for Theorem 2. Again, we assume (without loss of generality) that there are only 3 break points, that is m=3, and present the proof for the middle break fraction, ${\overset{ˆ}{\lambda }}_{2}$.

Define $V_{\epsilon }$ and $V_{\epsilon }\left(C\right)$ as in the proof of Theorem 2. Using the same logic as the proof of Theorem 2, it suffices to consider the behaviour of $S_{T}\left(T_{1},T_{2},T_{3}\right)$ over $V_{\epsilon }$ for which $\left|T_{i}-T_{i}^{0}\right|<\epsilon T$ for all i. As before, we restrict attention to the case in which $T_{2}<T_{2}^{0}$. The desired result can be established if it can be shown that for each η>0, there exist C>0 and ϵ>0 such that for large T,

(103) $$P\left(\min\left\{\left[S_{T}\left(T_{1},T_{2},T_{3}\right)-S_{T}\left(T_{1},\overset{0}{\underset{2}{T}},T_{3}\right)\right]/\left(T_{2}^{0}-T_{2}\right)\right\}<0\right)<\eta$$

where the minimum is taken over the set $V_{\epsilon }\left(C\right)$.

It is possible to follow the same steps as in the proof of Theorem 2 to show that

(104) $$\frac{{\mathrm{SSR}}_{1}-{\mathrm{SSR}}_{2}}{T_{2}^{0}-T_{2}}\geq 2^{-1}{\left(\beta_{3}^{0}-\beta_{2}^{0}\right)}^{\prime }\;\left[W_{△}^{\prime }W_{△}/\left(T_{2}^{0}-T_{2}\right)\right]\;\left(\beta_{3}^{0}-\beta_{2}^{0}\right)-\epsilon O_{p}\left(1\right)-\rho O_{p}\left(1\right)$$

with large probability. It can be shown that the first term on the right hand side of (104) dominates, and that

(105) $${\left(T_{2}^{0}-T_{2}\right)}^{-1}W_{△}^{\prime }W_{△}\geq \min\left\{\alpha_{1}\gamma_{1},\;\alpha_{2}\gamma_{2}\right\}\left({\left\|\overset{\left(i\right)}{\underset{0}{\Delta }}\right\|}^{2}+{\left\|\overset{\left(i+1\right)}{\underset{0}{\Delta }}\right\|}^{2}\right)+o_{p}\left(1\right)$$

where $\gamma_{1}$ and $\gamma_{2}$ are the smallest eigenvalues of ${\left(T_{i}^{*}-T_{2}\right)}^{-1}\sum_{1}z_{t}z_{t}^{\prime }$ and ${\left(T_{2}^{0}-T_{i}^{*}\right)}^{-1}\sum_{2}z_{t}z_{t}^{\prime }$, respectively, and $\alpha_{1}=\left(T_{i}^{*}-T_{2}\right)/\left(T_{2}^{0}-T_{2}\right),\alpha_{2}=\left(T_{2}^{0}-T_{i}^{*}\right)/\left(T_{2}^{0}-T_{2}\right)$. From Assumption 19(ii)–(iii), it follows that the first term on the right hand side of (105) is bounded away from zero on $V_{\epsilon }\left(C\right)$ with large probability. Therefore, the first term on the right hand side of (104) dominates and is positive for large C, small ϵ and large T which in turn proves (103).

*Part* (iii): It can be shown that

$$T^{1/2}\left({\overset{ˆ}{\beta }}_{i}-\beta_{i}^{0}\right)={\left(\;T^{-1}\overset{\left[\lambda_{i}^{0}T\right]}{\underset{t=\left[\lambda_{i-1}^{0}T\right]+1}{\sum }}{\overset{ˆ}{x}}_{t}\left(\pi^{0}\right){\overset{ˆ}{x}}_{t}{\left(\pi^{0}\right)}^{\prime }\;\right)}^{-1}T^{-1/2}\overset{\left[\lambda_{i}^{0}T\right]}{\underset{t=\left[\lambda_{i-1}^{0}T\right]+1}{\sum }}{\overset{ˆ}{x}}_{t}\left(\pi^{0}\right){\overset{˜}{u}}_{t}\left(\pi^{0}\right)+o_{p}\left(1\right)$$

and

$$T^{-1}\overset{\left[\lambda_{i}^{0}T\right]}{\underset{t=\left[\lambda_{i-1}^{0}T\right]+1}{\sum }}{\overset{ˆ}{x}}_{t}\left(\pi^{0}\right){\overset{ˆ}{x}}_{t}{\left(\pi^{0}\right)}^{\prime }\overset{p}{\to }\Theta_{0}^{\prime }{\overset{˜}{Q}}_{i}\Theta_{0}$$

$$T^{-1/2}\overset{\left[\lambda_{i}^{0}T\right]}{\underset{t=\left[\lambda_{i-1}^{0}T\right]+1}{\sum }}{\overset{ˆ}{x}}_{t}\left(\pi^{0}\right){\overset{˜}{u}}_{t}\left(\pi^{0}\right)=\Theta_{0}^{\prime }{\overset{˜}{C}}_{i}T^{-1/2}\overset{\left[\lambda_{i}^{0}T\right]}{\underset{t=\left[\lambda_{i-1}^{0}T\right]+1}{\sum }}{\overset{˜}{h}}_{t}-{\overset{˜}{Q}}_{i}{\overset{˜}{Q}}_{ZZ}{\left(1\right)}^{-1}{\overset{˜}{D}}_{i}\overset{T}{\underset{t=1}{\sum }}{\overset{˜}{h}}_{t}+o_{p}\left(1\right)\text{.}$$

 The result then follows after some manipulations.

Proof of Theorem 9 Using similar arguments to the proof of Theorem 8(iii), it follows that under $H_{0}$ we have

$$T^{1/2}\left[{\overset{ˆ}{b}}_{1}\left(j\right)-b\left(j\right)\right]={\overset{¯}{A}}_{1}\left\{{\left(\pi_{j}^{0}-\nu_{0}\right)}^{-1}T^{-1/2}\underset{1}{\sum }z_{t}\left[u_{t}+\overset{\prime }{\underset{t}{v}}b_{x}\left(j\right)\right]-{\left(\pi_{j}^{0}-\pi_{j-1}^{0}\right)}^{-1}T^{-1/2}\underset{r_{0}}{\sum }z_{t}\overset{\prime }{\underset{t}{v}}b_{x}\left(j\right)\right\}+o_{p}\left(1\right)$$

$$T^{1/2}\left[{\overset{ˆ}{b}}_{2}\left(j\right)-b\left(j\right)\right]={\overset{¯}{A}}_{2}\left\{{\left(\nu_{1}-\pi_{j}^{0}\right)}^{-1}T^{-1/2}\underset{2}{\sum }z_{t}\left[u_{t}+\overset{\prime }{\underset{t}{v}}b_{x}\left(j\right)\right]-{\left(\pi_{j+1}^{0}-\pi_{j}^{0}\right)}^{-1}T^{-1/2}\underset{r_{1}}{\sum }z_{t}\overset{\prime }{\underset{t}{v}}b_{x}\left(j\right)\right\}+o_{p}\left(1\right)$$

 where $\sum_{r_{0}}$ denotes summation over $t=\left[\pi_{j-1}^{0}T\right]+1,\ldots ,\left[\pi_{j}^{0}T\right]$ and $\sum_{r_{1}}$ denotes summation over $t=\left[\pi_{j}^{0}T\right]+1,\ldots ,\left[\pi_{j+1}^{0}T\right]$. □

## Footnotes

1 See *inter alia* Andrews and Fair (1988), Ghysels and Hall (1990), Andrews (1993), Sowell (1996) and Hall and Sen (1999).

2 Bai and Perron’s (1998) paper also contributes to the literature in statistics on change point estimation in time series. See *inter alia* Picard (1985), Hawkins (1986), Bhattacharya (1987), Yao (1987) and Bai (1994).

3 A similar comment applies to the recent extensions of Bai and Perron’s (1998) framework by Perron and Qu (2006) and Qu and Perron (2007).

4 We note that a similar analysis based on the second-step GMM minimand needs to consider the properties of the long run variance matrix estimator employed. Such an analysis is complicated by the issue of centring; for example see Hall et al. (2003) for an analysis of the impact of centring in covariance matrix estimation on the overidentifying restrictions test in the presence of structural instability. We anticipate similar problems arise here. Furthermore, it seems reasonable to anticipate that such an analysis of the second-step GMM minimand requires the type of analysis of the first-step estimator presented here, and that if the first-step estimation fails to identify the true break then this will undermine estimation of the break fraction on the second step.

5 For this model, $d_{i}=\sqrt{R^{2}/\left(q-q\times R^{2}\right)}$; see Hahn and Inoue (2002).

6 For the record, we note that the distribution looks qualitatively the same at T=10,000. Results are available from the authors upon request.

7 [⋅] denotes the integer part of the quantity in the brackets.

8 See, for example, Hall (2005)[p. 44].

9 Bai et al. (2008) present an analysis of the multiple break in models with measurement error. Note that while their orthogonality condition implies stability of a corresponding reduced form, their setting is different from ours since they consider the properties of sequential break-point estimators, while we rely on a global analysis.

10 This rests on showing that under the stated conditions $\left\{h_{t},G_{-\infty }^{t}\right\}$ is a mixingale of size −1/2 with constants $c_{T,j}=n\xi_{T,j}^{-1/2}\max\left(1,{\left\|b_{t,j}\right\|}_{r}\right)$; see Wooldridge and White (1988).

11 See e.g. Hall (2005)[p. 35].

12 See Andrews (1991) for details of HAC estimators.

13 “⇒” denotes weak convergence in the space D[0,1] under the Skorohod metric.

14 *UDmax* denotes Unweighted Double maximum. Bai and Perron (1998) also consider a *WDmax* statistic in which the maximum is taken over weighted values of the $\mathrm{Sup}\text{-}F_{T}\left(k;p\right)$ statistics. Analogous *WDmax* statistics can be developed within our framework, but for brevity we do not explore them here.

15 Within this model, it can be shown that $S_{i,i}=\left(\lambda_{i}^{0}-\lambda_{i-1}^{0}\right)\left\{V_{1,1}+\left(1+\lambda_{i-1}^{0}-\lambda_{i}^{0}\right)\;\left[\left(\beta_{i}^{0^{\prime }}⊗I_{q}\right)V_{2,2}\left(\beta_{i}^{0}⊗I_{q}\right)+2V_{1,2}\left(\beta_{i}^{0}⊗I_{q}\right)\;\right]\right\}$ and $S_{\left(i,j\right)}=-\left(\lambda_{i}^{0}-\lambda_{i-1}^{0}\right)\left(\lambda_{j}^{0}-\lambda_{j-1}^{0}\right)\left[V_{1,2}\left(\beta_{j}^{0}⊗I_{q}\right)+\left(\beta_{i}^{0^{\prime }}⊗I_{q}\right)V_{2,1}+\left(\beta_{i}^{0^{\prime }}⊗I_{q}\right)\times \;V_{2,2}\left(\beta_{j}^{0}⊗I_{q}\right)\right]$ where $V=\left(\begin{array}{cc} V_{1,1} & V_{1,2}\\ V_{1,2}^{\prime } & V_{2,2} \end{array}\right)$ is thelong-run covariance of $T^{-1/2}\sum_{t=1}^{T}{\left(u_{t},v_{t}^{\prime }\right)}^{\prime }⊗z_{t},V_{1,1}$ is q×q and $V_{2,2}$ is $qp_{1}\times qp_{1}$. Consistent estimators of $S_{i,j}$ are constructed using these formulae in the obvious fashion.

16 As in the one break model, the results are the same whether the $\mathrm{Sup}\text{-}F_{T}\left(1;p\right)\;\left(\mathrm{Sup}\text{-}{\mathrm{Wald}}_{T}\left(1;p\right)\right)$ or the ${\mathrm{UDmaxF}}_{T}\left(5,1\right)\;\left({\mathrm{UDmaxWald}}_{T}\left(5,1\right)\right)$ statistic is used on the first step.

17 The consequences of the nonlinearity of such limits have been explored in the context of single break point tests by Hansen (2000).

18 In calculating the tests, the sub-sample $T_{j}$ is treated as the entire sample and so the sample size is ${\overset{ˆ}{\tau }}_{j}-{\overset{ˆ}{\tau }}_{j-1}$.

19 See the discussion following Theorem 9.

20 As Zhang et al. (2008) note, the inclusion of further lags of inflation also mitigates the issue of weak instruments, a problem commonly encountered when estimating stylized versions of NKPC—see e.g. Kleibergen and Mavroeidis (2009) and the references therein.

21 We thank an anonymous referee for pointing out this issue.

22 One interesting aspect of the study of Zhang et al. (2008) is that they employ various different inflation forecasts in their estimation. We focus here on just one of their choices for brevity.

23 The 5% level critical value for this test is 13.97; see Stock and Yogo (2005).

24 These calculations are made using the code available from http://people.bu.edu/perron/code.html. All hypotheses are tested with F-statistics which are the OLS analogs of those discussed in the text; further details can be found in Bai and Perron (1998).

25 For ease of presentation, we define the BIC criterion below for 2SLS; the appropriate modification for OLS is then obvious.

26 The results for the first two samples are omitted because these samples are quite small in relation to the number of parameters.

27 We note that with other choices of inflation forecast series, Zhang et al. (2008) find evidence of breaks at other points in the sample.
